# Site‐Selective, Modular Diversification of Polyhalogenated Aryl Fluorosulfates (ArOSO_2_F) Enabled by an Air‐Stable Pd^I^ Dimer

**DOI:** 10.1002/anie.201911465

**Published:** 2019-12-16

**Authors:** Marvin Mendel, Indrek Kalvet, Daniel Hupperich, Guillaume Magnin, Franziska Schoenebeck

**Affiliations:** ^1^ Institute of Organic Chemistry RWTH Aachen University Landoltweg 1 52074 Aachen Germany

**Keywords:** aryl fluorosulfates, catalysis, chemoselectivity, DFT calculations, dinuclear Pd^I^

## Abstract

Since 2014, the interest in aryl fluorosulfates (ArOSO_2_F) as well as their implementation in powerful applications has continuously grown. In this context, the enabling capability of ArOSO_2_F will strongly depend on the substitution pattern of the arene, which ultimately dictates its overall function as drug candidate, material, or bio‐linker. This report showcases the modular, substrate‐independent, and fully predictable, selective functionalization of polysubstituted arenes bearing C−OSO_2_F, C−Br, and C−Cl sites, which makes it possible to diversify the arene in the presence of OSO_2_F or utilize OSO_2_F as a triflate surrogate. Sequential and triply selective arylations and alkylations were realized within minutes at room temperature, using a single and air‐stable Pd^I^ dimer.

## Graphical abstract

**Diversity**: The rapid, modular, substrate‐independent and fully predictable functionalization of polysubstituted arenes bearing C−OSO_2_F, C−Br, and C−Cl sites at room temperature is showcased. In this way the arene unit can be functionalized in the presence of a fluorosulfate group or the latter can serve as a triflate surrogate.

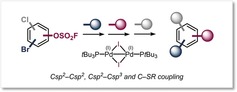

Owing to the exceptional properties and specific reactivity features of the S^VI^–F functionality^1^ as well as its vastly improved synthetic accessibility,^2^ a wide variety of powerful applications have recently been unlocked, harnessing aryl fluorosulfates as valuable handles for selective bioconjugation,^3^ polymerization,^4^ and fluorination^5^ and as pseudohalides in metal‐catalyzed cross‐coupling reactions.^6^ Aside from their enabling utility in transformative science, aryl fluorosulfates are also known for their bioactivity, finding usage as irreversible enzyme inhibitors^7^ and furthermore enabling “inverse” drug discovery strategies (see Figure 1).^8^ Consequently, a technology that is capable of selectively modifying the arene unit in a highly modular, practical, and general fashion, while tolerating the pre‐installed OSO_2_F functionality could enable the rapid generation of a library of densely functionalized aryl fluorosulfates, which in turn would accelerate the identification of novel bioactives, materials, bio‐linkers and the discovery of new functions, as this is ultimately dependent on the substitution pattern.

**Figure 1:**
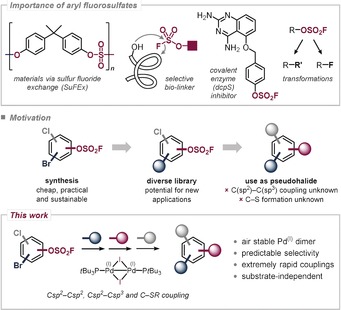
Importance of aryl fluorosulfates in materials, biochemistry, and transformations (top), motivation for site‐selective cross‐coupling in the presence of the OSO_2_F group and by using the OSO_2_F group (middle), and this work (bottom).

Conversely and in line with the ever increasing demand for sustainability and practicability, the selective functionalization with OSO_2_F would be equally powerful as a surrogate of the expensive triflate group, which is associated with poorer atom economy^9^ and the creation of hazardous, expensive fluorocarbon waste.^10^ While metal‐catalyzed aminations, arylations, and carbonylations of aryl fluorosulfates have been developed, to date, no C${}_{\mathrm{sp}{}^{2}}$ –C${}_{\mathrm{sp}{}^{3}}$ coupling (alkylation) or alternative carbon–heteroatom bond‐forming reactions exist, which would be highly attractive for the exploration of three‐dimensional chemical space. Moreover, in terms of site‐selective transformations, there is only a single documentation by Sharpless, in which the Suzuki‐based arylation of C−Br vs. C−OSO_2_F vs. C−Cl in pyridines has been showcased,6g employing relatively high catalyst loading and requiring refluxing toluene over several hours. However, our further examination of this protocol indicated that while effective for pyridines, our attempts to couple other arenes resulted in unpredictable and substrate‐dependent functionalization of C−Br and/or C−OSO_2_F (see Supporting Information Table S1).

These observations are in line with the general Pd^0^/Pd^II^ catalysis reactivity trends, which historically suffer from low predictability in the relative selectivity of C−Br vs. C−Cl vs. C−OTf, as they are strongly influenced by the catalyst, reaction conditions and, most importantly, by the steric and electronic factors of the specific substrate.^11^ Consequently, even if an identified protocol was selective for a given substrate, slight variations in sterics may change selectivity (as observed above, see the Supporting Information). By contrast, our group recently showcased that the use of the air‐stable, dinuclear [Pd^I^(μ‐I)P(*t*Bu)_3_]_2_ dimer **1** allows for extremely rapid, fully controlled, and substrate‐independent coupling of C−Br vs. C−Cl vs. C−OTf in arenes (Figure 1).^12^ As an extension of this work, we were keen to explore the potential of Pd^I^ in realizing the first general, substrate‐independent, and selective functionalization of C−OSO_2_F vs. C−Br/C−Cl in arenes (Figure 2).

**Figure 2:**
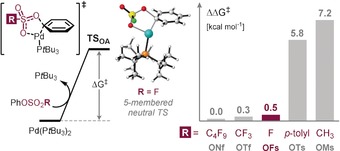
Reactivity scale for oxidative addition to Ar‐OSO_2_R derivatives, involving five‐membered neutral transition states with Pd^0^P(*t*Bu_3_). Free energy differences are given relative to the TS of PhONf in kcal mol^−1^. [OFs=OSO_2_F].

Previous reports suggested on the basis of qualitative reactivity comparisons, such as competition experiments, that aryl fluorosulfates would react similarly to aryl triflates in cross‐couplings.6d, 6g To get a more quantitative reactivity picture, we initially computationally^13^ assessed the activation free energy barrier for oxidative addition to C−OSO_2_F (commonly abbreviated as C−OFs) with Pd^0^P(*t*Bu)_3_ as a model catalyst, relative to alternative C−OR electrophiles, that is, triflates, nonaflates, mesylates, and tosylates. Calculations at SMD (toluene) M06/def2‐TZVP//ωB97XD/6‐31G(d) (SDD) level of theory suggested a five‐membered transition state (TS) arrangement to be favored for C−OSO_2_F activation and predicted its activation barriers to be virtually identical to that of triflates and nonaflates, reinforcing that OFs should be an excellent low‐cost surrogate of these groups.

We subsequently embarked on experimentally testing the OSO_2_F (=OFs) group in C−Br selective C−C coupling under Pd^I^ dimer **1** catalysis. Following our previous developments,12a–12d we added freshly prepared phenyl organozinc in excess and in one portion to a solution of 4‐bromo‐3,5‐dimethylphenyl fluorosulfate and 2.5 mol % Pd^I^ dimer **1** in dry toluene in the presence of air. This resulted in exclusive and extremely rapid functionalization of the C−Br site to give compound **2** in less than 5 minutes at room temperature and in air (see Table 1). The extreme speed and operational simplicity of the transformation suggested that it could indeed have potential in rapid aryl fluorosulfate diversification. As such, we next investigated the generality of the method. Table 1 summarizes the results. Pleasingly, we were able to perform selective arylations of the C−Br site for a range of arenes and pharmaceutically and agrochemically important heterocycles, irrespective of additional steric hindrance (**2**, **3**) or the relative positioning of C−Br vs. C−OSO_2_F and C−Cl. Even when the C−Br site was *ortho* to C−OSO_2_F (**5**, **8**) or the C−OSO_2_F site was electronically more activated (**4**, **8**), exclusive coupling at C−Br was seen. Regardless of how many equivalents of cross‐coupling partner were added, there was no overcoupling. As such, the functionalization appears to be a priori predictable for C−Br and independent of the steric or electronic bias imposed by the substrate.

**Table 1:** C−Br selective aryl‐, alkyl‐, and thiolations in the presence of the C−OSO_2_F (=OFs) functionality and other functionalities. 
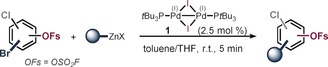

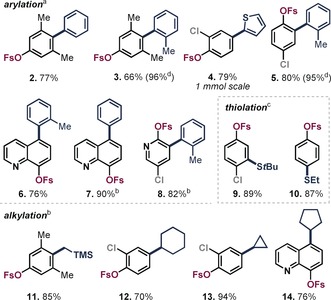

Conditions: [a] 2.0–2.5 equiv of organozinc and fast addition, in air. [b] 1.5–2.0 equiv of organozinc and slow addition over 10 min via syringe pump, argon atmosphere. [c] Conditions: **1** (5 mol %), NaSR (1.5 equiv), ZnCl_2_ (1.6 equiv), LiCl (1.6 equiv), toluene (1 mL), 40 °C, 6–8 h. [d] Yield determined by quantitative ^19^F NMR analysis.

Given the importance of alkylations, we next investigated whether the C−Br selectivity holds also for alkyl‐based organometallic nucleophiles. In Pd^0^/Pd^II^‐based alkylations of aryl halides, β‐hydride elimination from Pd^II^ intermediates as well as the inherent driving force for metal–halogen exchange from the alkyl organometallic to the aryl halide are well‐known challenges.^14^ Despite significant progress in this field,^15^ there are only two reports of general and predictable site‐selective alkylations of poly(pseudo)halogenated arenes, both based on Pd^I^ dimer catalysis developed by our group.12b, 12c Pleasingly, the alkylation with primary and secondary organozinc reagents proved to be equally powerful for aryl fluorosulfates, functionalizing only C−Br and leaving the C−OSO_2_F site (and alternative functionality) completely untouched (**11**–**14**).^16^, ^17^ Moreover, with prolonged reaction times and slightly elevated temperature, C−Br selective thiolations were also possible (**9**, **10**).^18^ These are the first examples of selective carbon–heteroatom bond formation and alkylations in the presence of C−OSO_2_F.

Aside from functionalization in the presence of C−OSO_2_F, its further conversion as a triflate or nonaflate surrogate in cross‐coupling reactions would also be of interest. Moving to the polar solvent NMP allowed the C${}_{\mathrm{sp}{}^{2}}$ ‐C${}_{\mathrm{sp}{}^{2}}$ as well as C${}_{\mathrm{sp}{}^{2}}$ ‐C${}_{\mathrm{sp}{}^{3}}$ coupling of the aryl fluorosulfates (see Table 2).12c We speculate that the Pd^I^ dimer converts under these conditions to a bisligated “ate” or NMP‐coordinated complex through rapid activation.^19^, ^20^ Notably, the reaction was complete within 10 min at room temperature and fully selective, tolerating the presence of C−Cl.^16^ Once again, the site selectivity was found to be independent of the electronic and steric influence of the substrate and the corresponding products were isolated in high yield. Aromatic and heterocyclic motifs (**16**, **17**, **18**) as well as *ortho* substitution (**21**, **22**) were well tolerated.^21^ As such, the first alkylation of the OSO_2_F group is presented, which is characterized by high practicability, rapidness, mildness, and chemoselectivity.

**Table 2:** C−OFs (=C−OSO_2_F) selective arylations and alkylations. 
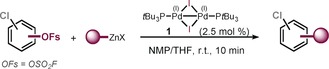

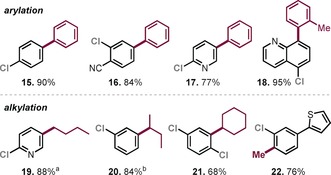

Conditions: slow addition of organozinc (1.2–2.0 equiv) over 10 min via syringe pump, argon atmosphere. [a] Around 5–10 % biscoupling detected by GCMS reaction control. [b] Contains 10 % of the linear isomer.

Owing to the ubiquitous abundance of polyfunctionalized arenes as key motifs in pharmaceuticals, materials, and natural products, a straightforward, rapid, and modular approach towards polysubstituted arenes is in high demand. Given the exquisite selectivity observed above, we next focused on performing sequential couplings with minimal disruptions to the reaction setup. To this end, we initially undertook C−Br functionalization by slow addition of the cross‐coupling partner to a solution of substrate and Pd^I^ dimer in toluene. After an additional 15 minutes of stirring, a mixture of the next organometallic cross‐coupling partner in NMP was added to the mixture, which resulted in selective OFs functionalization. The products arising from this one‐pot, sequential double functionalization were subsequently isolated in good to excellent yields (**23**–**26**, Table 3), requiring a total of 35 minutes at room temperature for their generation without intermediate workup or change of catalyst. In analogy to our previous report,12c we also executed the final step of C−Cl functionalization at 80 °C within 10 min and using the same Pd^I^ dimer **1** to generate the trisubstituted aromatic compounds in excellent yield over three coupling steps (**27**, **28**, Table 3).

**Table 3:** One‐pot doubly and add‐on triply selective sequential functionalizations. 
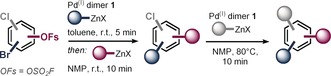

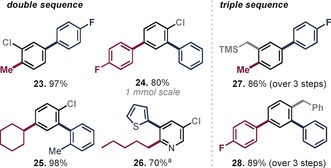

Conditions: **1** (2.5 mol %), Ar‐OFs (0.2 mmol), organozinc (1.2–2.0 equiv), C−Br coupling in toluene/THF, C−OFs in toluene/NMP/THF, C−Cl in NMP/THF at 80 °C, argon atmosphere. [a] Additional aqueous workup was performed. For detailed experimental procedure see the Supporting Information.

In conclusion, the rapid, operationally simple, air‐tolerant, and chemoselective C${}_{\mathrm{sp}{}^{2}}$ –C${}_{\mathrm{sp}{}^{2}}$ , C${}_{\mathrm{sp}{}^{2}}$ –C${}_{\mathrm{sp}{}^{3}}$ , and C−SR coupling of poly(pseudo)halogenated arenes bearing C−Br, C−OSO_2_F, and C−Cl sites has been showcased, allowing for diversification in the presence of the valuable C−OSO_2_F functionality, as well as selective functionalization of C−OSO_2_F as a low‐cost and more atom‐economic surrogate for triflates. Key to this exquisite reactivity was the employment of the air‐stable Pd^I^ dimer **1**, which facilitated selective couplings within minutes at room temperature. The modular triply selective functionalization in the sequence C−Br, then C−OFs, then C−Cl was also demonstrated. Given the generality, selectivity, robustness, and high speed, we anticipate widespread application of this methodology in academic and industrial research.

## Conflict of interest

The authors declare no conflict of interest.

## Supporting information

As a service to our authors and readers, this journal provides supporting information supplied by the authors. Such materials are peer reviewed and may be re‐organized for online delivery, but are not copy‐edited or typeset. Technical support issues arising from supporting information (other than missing files) should be addressed to the authors.

Supplementary
