# Supplementary material for: Site‐Selective, Modular Diversification of Polyhalogenated Aryl Fluorosulfates (ArOSO2F) Enabled by an Air‐Stable PdI Dimer
Source: Angew Chem Int Ed Engl. 2019 Dec 16;59(5):2115–9. doi: 10.1002/anie.201911465 (PMC7003813; doi:10.1002/anie.201911465)

Angewandte Chemie
Eine Zeitschrift der Gesellschaft Deutscher Chemiker

Supporting Information

Site-Selective, Modular Diversification of Polyhalogenated Aryl Fluorosulfates ($ArOSO_2F$) Enabled by an Air-Stable Pd^I Dimer

*Marvin Mendel, Indrek Kalvet, Daniel Hupperich, Guillaume Magnin, and Franziska Schoenebeck**

anie_201911465_sm_miscellaneous_information.pdf

Contents

General Experimental Details 2

Investigation on a general Br vs. OFs vs. Cl selective strategy 3

General Experimental Procedures 4

Synthesis of Aryl Fluorosulfates Starting Material 4

General Procedure A (C–Br Site-selective Arylation on Air) 4

General Procedure B (C–Br Site-selective Aryl-/Alkylation) 5

General Procedure C (C–OFs Site-selective Aryl-/Alkylation) 5

General Procedure D (C–Br Site-selective Thiolation) 5

General Procedure E (Double/Triple Sequential Coupling) 6

Preparation of Organozinc Reagent 6

Synthesis of Sodium Aryl and Alkyl thiolates 7

Compound Characterization Data 7

Aryl Fluorosulfates 7

C–Br Site-selective Aryl- and Alkylation Products 10

C–Br Site-selective Thiolation Products 13

C–OFs Site-selective Aryl- and Alkylation Products 13

Modular Sequential Coupling Products 15

Computational Studies 17

Cartesian coordinates and energies of computed structures 17

References 24

NMR spectra 25

Aryl fluorosulfates 25

Coupling products 36

General Experimental Details

Reagents and Starting Material

All reagents and starting materials were commercially available and used as received. $Pd^{(I)}$-iodo-dimer **1** was prepared according to the literature procedure.[1] Anhydrous solvents were dried using an Innovative Technology PS-MD-5 solvent purification system. Solvents used in work up and purification were received in technical grade and distilled prior to use. Thin layer chromatography (TLC) was performed on Merck Kieselgel 60 F_{254} aluminium plates with unmodified silica and visualized under UV light. Flash column chromatography was performed with Merck silica gel 60 (35 – 70 mesh).

Experimental Techniques

The work-up of all reactions and the isolation of products were carried out in a fume hood using standard techniques. Whether a reaction was performed under an argon or air atmosphere is specified in the experimental procedure.

Characterization

All ^{1}H, ^{13}C and ^{19}F NMR spectra were recorded on Varian VNMRS 600, Varian VNMRS 400 or Varian Mercury 300 spectrometers at ambient temperature. Chemical shifts (δ) are reported in parts per million (ppm) and were referenced to residual solvent peak. Coupling constants (*J*) are given in Hertz (Hz) and coupling patterns are described as s = singlet, d = doublet, t = triplet, q = quartet, m = multiplet.

Gas chromatography coupled with mass spectrometry (GC-MS) was performed on an Agilent Technologies 5975 series MSD mass spectrometer under electrospray ionization (EI) mode coupled with an Agilent Technologies 7820A gas chromatograph employing an Agilent 19091s-433 HP-5MS column (30 m x 0.250 µm x 0.250 µm). High-resolution mass spectrometry (HRMS) was performed using a Thermo Scientific LTQ Orbitrap XL spectrometer. Low-resolution masses of known compounds were extracted from their GC-MS chromatograms. Melting points were measured with a Coesfeld melting point meter (MPM-H2) with visual detection and temperature increase of 1 °C/min.

Investigation on a general Br vs. OFs vs. Cl selective strategy

Table S1: Application of previously reported strategies for the site-selective transformation of aryl fluorosulfates.

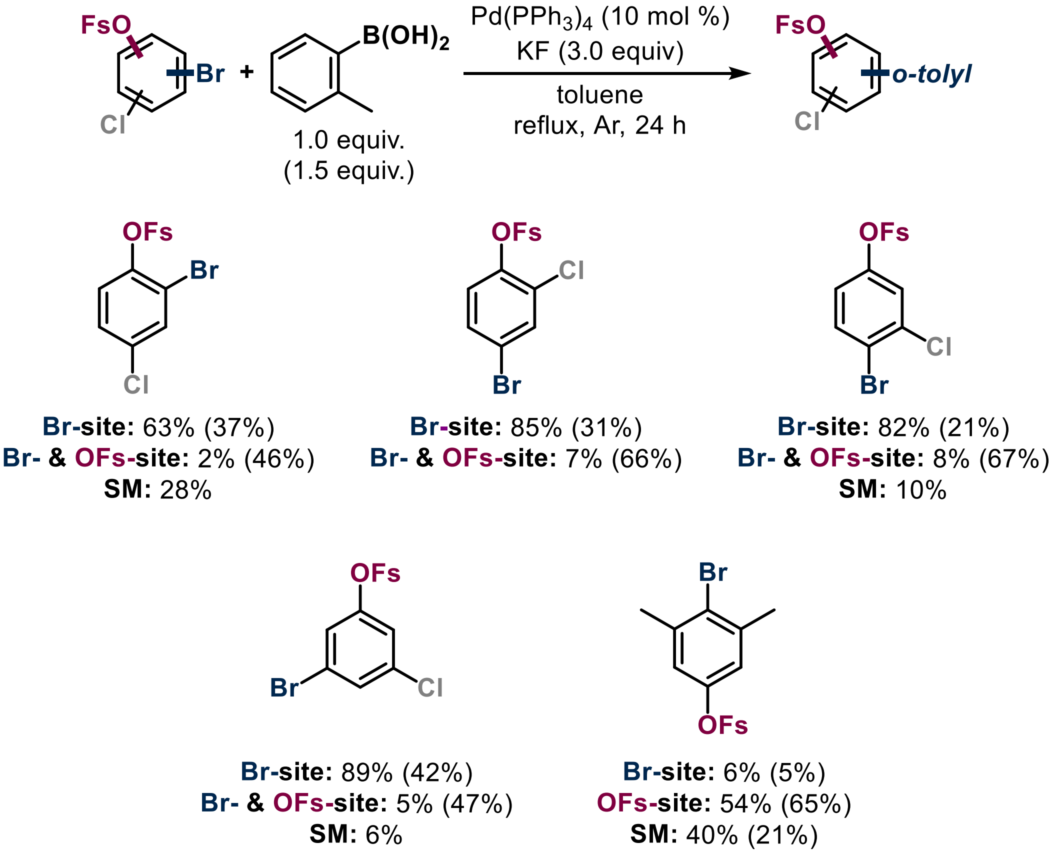

Reactions were performed in accordance with the procedure reported by *Sharpless and co-workers.*[2]

Reaction Conditions: Under inert atmosphere the aryl fluorosulfate (0.1 mmol) was placed together with the boronic acid (1.5 equiv, 0.15 mmol, 20.4 mg), $Pd(PPh_3)_4$ (10 mol %, 11.6 mg) and KF (3.0 equiv, 0.3 mmol, 17.4 mg) to a 4 mL vial equipped with a magnetic stirring bar. Subsequently, toluene (1 mL) was added and the reaction mixture was heated to reflux for 24 hours. The solvent was then removed *in vacuo*, the internal standard 4-(trifluoromethoxy)-anisole (0.5 equiv, 0.05 mmol, 9.6 mg) and $CDCl_3$ were added and the mixture was filtered through a short plug of celite (3-4 cm in a Pasteur pipette). The reaction mixture was then analyzed using GC-MS and ^{1}H (and ^{19}F) NMR spectroscopy used to determine the extent of conversion to the products.

General Experimental Procedures

Synthesis of Aryl Fluorosulfates Starting Material

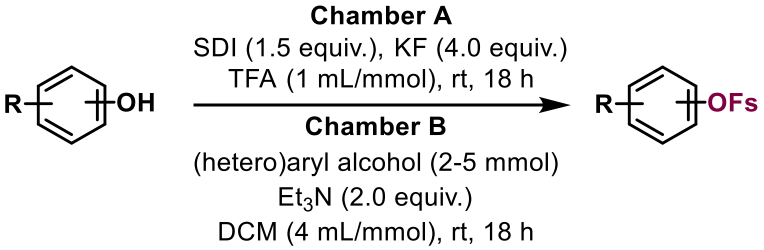

According to *Veryser et al.*[3] chamber A of a flame-dried two-chamber reactor was filled with 1,1'-sulfonyldiimidazole (1.49 g, 7.5 mmol, 1.5 equiv.) and potassium fluoride (KF, 1.16 g, 20.0 mmol, 4.0 equiv.). Next, chamber B was charged with the appropriate (hetero)aryl alcohol (5.0 mmol) solubilized in dichloromethane (DCM, 20 mL, 0.25 M) and treated with triethylamine (1.39 mL, 10.0 mmol, 2.0 equiv.). Finally, trifluoroacetic acid (5 mL, 3.26 M) was added by injection through the septum in chamber A (Caution! Instant gas formation!). After 18 hours of stirring at room temperature, one of the caps was carefully removed inside a well ventilated fume hood to release the residual pressure. The reaction was stirred for another 15 min to ensure that all sulfuryl fluoride could evaporate. Chamber A was neutralized with aqueous $NaHCO_3$ (sat.). Next, the content of chamber B was transferred to a 100 mL round-bottomed flask. Chamber B was rinsed five times with 2 mL of dichloromethane. The organic layers were combined and the solvent was removed under reduced pressure. The crude product was purified by solid-phase flash column chromatography on silica gel.

General Procedure A (C–Br Site-selective Arylation on Air)

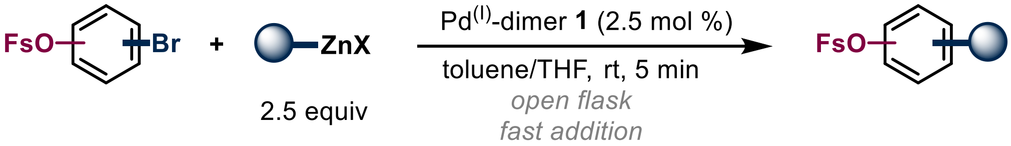

The appropriate aryl fluorosulfate (0.2 mmol, 1.0 equiv.) and the Pd(I)-iodo-dimer **1** catalyst (4.4 mg, 0.005 mmol, 0.025 equiv.) were placed into a vial and solubilized in toluene (2 mL). No efforts were made to exclude air. Then, a freshly prepared solution of appropriate organozinc was added fast to the reaction mixture. It was stirred for 5 min, before it was quenched with pentane. Ammonium pyrrolidine-1-dithiocarboxylic acid was added to precipitate palladium and the mixture was filtered through a plug of silica.[4] The filtrate was concentrated under reduced pressure and the crude material was purified by column chromatography on silica gel.

General Procedure B (C–Br Site-selective Aryl-/Alkylation)

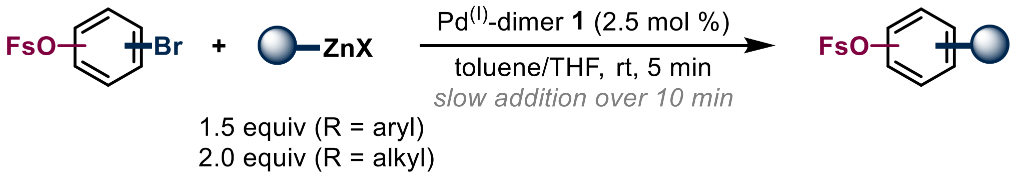

Aryl fluorosulfate (0.2 mmol, 1.0 equiv.) and Pd(I)-iodo-dimer **1** catalyst (4.4 mg, 0.005 mmol, 0.025 equiv.) were placed into a vial. It was flushed with argon and solubilized in toluene (2 mL). Then, a freshly prepared solution of appropriate organozinc was added slowly to the reaction mixture via syringe pump (over 10 min). The reaction mixture was stirred for additional 5 min, before it was quenched with pentane. Ammonium pyrrolidine-1-dithiocarboxylic acid was added to precipitate palladium and the mixture was filtered through a plug of silica.[4] The filtrate was concentrated under reduced pressure and the crude material was purified by silica gel column chromatography.

General Procedure C (C–OFs Site-selective Aryl-/Alkylation)

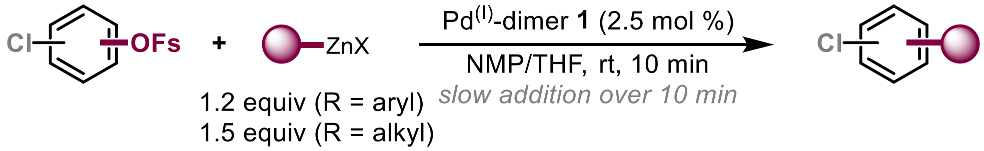

Aryl fluorosulfate (0.2 mmol, 1.0 equiv.) and Pd(I)-iodo-dimer **1** catalyst (4.4 mg, 0.005 mmol, 0.025 equiv.) were placed to a vial. It was flushed with argon and solubilized in in NMP (2 mL). Then, a freshly prepared solution of appropriate organozinc was added slowly to the reaction mixture via syringe pump (over 10 min). The reaction mixture was stirred for extra 10 min, before it was quenched with pentane. Ammonium pyrrolidine-1-dithiocarboxylic acid was added to precipitate palladium and the mixture was filtered through a plug of silica.[4] The filtrate was concentrated under reduced pressure and the crude material was purified by silica gel column chromatography.

General Procedure D (C–Br Site-selective Thiolation)

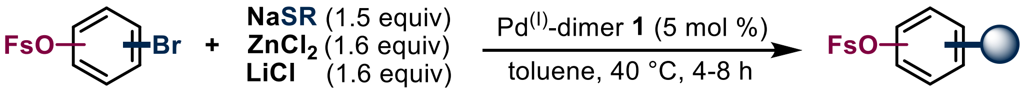

To an oven dried 8 mL vial equipped with a stirrer bar, the bromoaryl fluorosulfate (0.2 mmol, 1.0 equiv) and Pd(I)-iodo-dimer **1** catalyst (8.7 mg, 0.01 mmol, 0.05 equiv.) were placed. Then, the reaction vessel was introduced to the glovebox and the appropriate sodium thiolate (1.5 equiv) was added. The mixture was suspended in toluene (1 mL). Subsequently, $ZnCl_2$ (1.6 equiv, 1M in THF) and LiCl (1.6 equiv, 0.5M in THF) were added. The vial was closed and sealed with teflon tape. In addition, it was stirred for 4–8 hours at 40 °C. After the indicated time the reaction mixture was diluted with 2 mL pentane and ammonium pyrrolidine-1-dithiocarboxylic acid was added to precipitate palladium.[4] The mixture was filtered through a plug of silica and solvent was concentrated under reduced pressure. The crude was then purified by column chromatography with indicated solvent.

General Procedure E (Double/Triple Sequential Coupling)

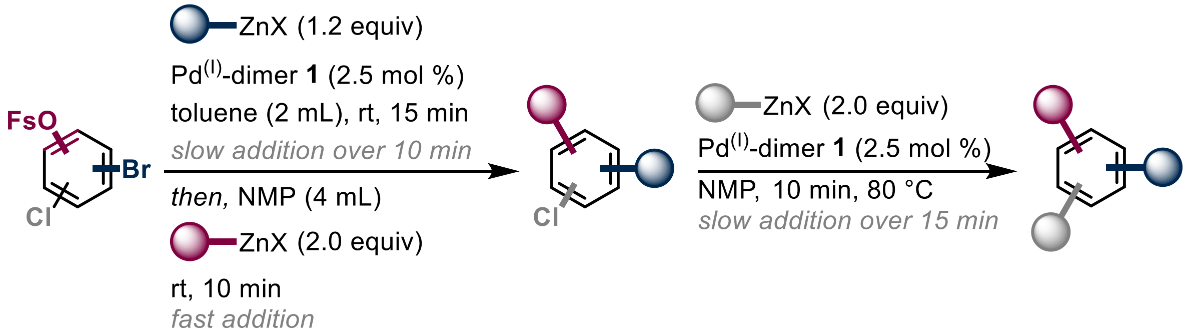

The appropriate aryl fluorosulfate (0.2 mmol, 1.0 equiv.) and Pd(I)-iodo-dimer **1** catalyst (4.4 mg, 0.005 mmol, 0.025 equiv.) were placed into a vial. It was flushed with argon and solubilized in toluene (2 mL). Then, a freshly prepared solution of appropriate organozinc was added slowly to the reaction mixture via syringe pump (over 10 min). The reaction mixture was stirred for additional 15 min to ensure full consumption of the organometallic reagent. During this time another organozinc reagent was prepared. Next, NMP (2 mL) was added to both the organozinc reagent and to the reaction mixture. Immediately, the diluted organozinc reagent was added fast to the reaction mixture and it was stirred for 10 min. The reaction was quenched with pentane and ammonium pyrrolidine-1-dithiocarboxylic acid was added to precipitate palladium.[4] The mixture was filtered through a plug of silica, the filtrate was concentrated under reduced pressure and the crude material was purified by silica gel column chromatography giving the double sequential coupling products.*

In accord with our group's previous work[5] the resulting aryl chloride was then placed together with Pd(I)-iodo-dimer **1** catalyst (4.4 mg, 0.005 mmol, 0.025 equiv.) into a vial and flushed with argon. It was solubilized in NMP (2 mL) and placed in a sand bath at 80 °C. The appropriate organozinc reagent was added by syringe pump over 15 min. In addition, it was stirred for an extra 10 min. The mixture was allowed to cool to room temperature and was then quenched with pentane and ammonium pyrrolidine-1-dithiocarboxylic acid was added to precipitate palladium.[4] After filtration through a plug of silica, the filtrate was concentrated under reduced pressure and the crude material was purified by silica gel column chromatography.

**1 mmol scale*: Additional aqueous work up was performed: Therefore, the reaction mixture was filtered through a plug of silica and transferred to a separating funnel using EtOAc (2x10 mL) to wash the reaction vial. To remove NMP the organic layer was extracted with water (5x50 mL). Then, it was dried over Na_2SO_4, filtered through cotton and the filtrate was concentrated under reduced pressure. The crude material was purified by silica gel column chromatography with pentane.

Preparation of Organozinc Reagent

A solution of the appropriate aryl magnesium halide (in either THF, 2-MeTHF or Et_2O, 1.0 equiv.), $ZnCl_2$ (1M in THF, 1.1 equiv.) and LiCl (0.5M in THF, 1.1 equiv.) were added to a dry 16 mL vial under argon atmosphere and stirred for 10 min.

Preparation of the $ZnCl_2$ solution:[6] To an oven dried Schlenk tube equipped with a stir-bar was added anhydrous $ZnCl_2$ (1.36 g, 10 mmol) under argon atmosphere. Upon melting under high vacuum using a

Bunsen burner, the tube was allowed to cool to room temperature and refilled with argon. Subsequently, anhydrous THF (10 mL) was added and it was stirred vigorously until a clear solution resulted.

Synthesis of Sodium Aryl and Alkyl thiolates

Under argon atmosphere, NaH (0.98 equiv, 12.50 mmol, 300 mg) was placed into a round bottom flask and was suspended in dry THF (30 mL). Subsequently, a solution of thiol (1.0 equiv, 12.71 mmol) in THF (3 mL) was added slowly (Caution! H_2 formation!). The mixture was stirred for 1 hour at room temperature. Then, the sodium thiolate was precipitated by addition of dry hexane (50 mL). The solid was collected by filtration and dried under vacuum. The corresponding thiolate was obtained in quantitative yield and was used without further purification.

Compound Characterization Data

Aryl Fluorosulfates

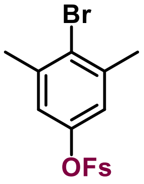

4-bromo-3,5-dimethylphenyl fluorosulfate: General procedure was followed using 4-bromo-3,5-dimethylphenol (1.02 g, 5.05 mmol). The title product was obtained after purification by column chromatography (Pentane) as a white solid. 1.31 g (91%). **R$_f$** = 0.53 (Pentane). **M.p.** = 57–58 °C. **^{1}H NMR** (600 MHz, $CDCl_3$) δ 7.08 (s, 2H), 2.46 (s, 6H). **^{13}C NMR** (151 MHz, $CDCl_3$) δ 148.2, 141.0, 127.3, 120.1, 24.1. **^{19}F NMR** (564 MHz, $CDCl_3$) δ 37.71. **MS** (70 eV, EI): *m/z* (%): 284 (82) [^{81}Br-M$^+$], 282 (78) [^{79}Br-M$^+$], 201 (69), 199 (69), 173 (97), 171 (100), 92 (45), 91 (88). **HRMS** (ESI): *m/z* [M]$^+$ calculated for $C_8H_8{}^{79}BrFO_3{}^{32}S$: 281.93561, found: 281.93633.

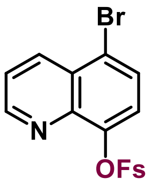

5-bromoquinolin-8-yl fluorosulfate: General procedure was followed using 5-bromo-quinolin-8-ol (1.12 g, 5 mmol). The title product was obtained after purification by column chromatography (20:1 Pentane/EtOAc) as a white solid. 1.12 g (73%). **R$_f$** = 0.32 (20:1 Pentane/EtOAc). **M.p.** = 117–118 °C. **^{1}H NMR** (400 MHz, $CDCl_3$) δ 9.09 (m, 1H), 8.59 (m, 1H), 7.89 (d, *J* = 8.3 Hz, 1H), 7.67–7.63 (m, 2H). **^{13}C NMR** (101 MHz, $CDCl_3$) δ 152.4, 145.4, 141.0, 135.8, 129.4, 129.2, 123.8, 122.3, 121.7. **^{19}F NMR** (376 MHz, $CDCl_3$) δ 41.05. **MS** (70 eV, EI): *m/z* (%): 307 (45) [^{81}Br-M$^+$], 305 (40) [^{79}Br-M$^+$], 224 (60), 222 (61), 196 (98), 194 (100), 116 (12), 115 (78), 114 (30), 89 (8), 88 (30), 87 (19), 83 (14). **HRMS** (ESI): *m/z* [M+H]$^+$ calculated for $C_9H_6{}^{79}BrFNO_3{}^{32}S$: 305.92303, found: 305.92303.

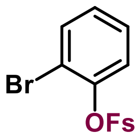

2-bromophenyl fluorosulfate: General procedure was followed using 2-bromophenol (500 mg, 2.89 mmol). The title product was obtained after purification by column chromatography (20:1 Pentane/EtOAc) as a colorless liquid. 606 mg (82%). **R$_f$** = 0.70 (10:1 Pentane/EtOAc). **^{1}H NMR** (600 MHz, $CDCl_3$) δ 7.70 (d, *J* = 7.9 Hz, 1H), 7.45–7.42 (m, 2H), 7.31–7.28 (m, 1H). **^{13}C NMR** (151 MHz, $CDCl_3$) δ 147.2, 134.6, 129.8, 129.2, 122.6, 115.5. **^{19}F NMR** (564 MHz, $CDCl_3$) δ 41.45. **MS** (70 eV, EI): *m/z* (%): 256 (83) [^{81}Br-M$^+$], 254 (76) [^{79}Br-M$^+$], 173 (79), 171 (73), 146 (100), 143 (94), 65 (6), 64 (26), 65 (45), 62 (14), 61 (6). **HRMS** (ESI): *m/z* [M]$^+$ calculated for $C_6H_4{}^{79}BrFNO_3{}^{32}S$: 253.90418, found: 253.90431. The data are in agreement with those previously reported in the literature.[3]

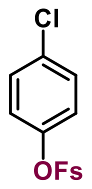

4-chlorophenyl fluorosulfate: General procedure was followed using 4-chlorophenol (321 mg, 2.5 mmol). The title product was obtained after purification by column chromatography (30:1 Pentane/EtOAc) as a colorless liquid. 444 mg (84%). **R_f** = 0.88 (10:1 Pentane/EtOAc). **^{1}H NMR** (600 MHz, $CDCl_3$) δ 7.46 (m, 2H), 7.30 (m, 2H). ^{13}C NMR (151 MHz, $CDCl_3$) δ 148.3, 134.6, 130.5, 122.3. **^{19}F NMR** (564 MHz, $CDCl_3$) δ 37.66. **MS** (70 eV, EI): *m/z* (%): 212 (24) [^{37}Cl-M$^+$], 210 (63) [^{35}Cl-M$^+$], 129 (33), 127 (100), 101 (27), 99 (81), 75 (13), 73 (21). **HRMS** (ESI): *m/z* [M]$^+$ calculated for $C_6H_4{}^{35}ClFNO_3{}^{32}S$: 209.95482, found 209.95386. The data are in agreement with those previously reported in the literature.[3]

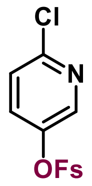

6-chloropyridin-3-yl fluorosulfate: General procedure was followed using 2-chloro-pyridin-5-ol (324 mg, 2.5 mmol). The title product was obtained after purification by column chromatography (50:1 Pentane/Et_2O) as a colorless liquid. 350 mg (66%). **R_f** = 0.57 (10:1 Pentane/Et_2O). **^{1}H NMR** (600 MHz, $CDCl_3$) δ 8.47 (d, *J* = 3.0 Hz, 1H), 7.69 (dd, *J* = 8.7, 3.0 Hz, 1H), 7.48 (d, *J* = 8.7 Hz, 1H). **^{13}C NMR** (151 MHz, $CDCl_3$) δ 151.2, 145.9, 142.5, 131.5, 125.8. **^{19}F NMR** (564 MHz, $CDCl_3$) δ 38.65. **MS** (70 eV, EI): *m/z* (%): 213 (22) [^{37}Cl-M$^+$], 211 (57) [^{35}Cl-M$^+$], 130 (10), 128 (29), 102 (33), 100 (100), 75 (14), 73 (39). **HRMS** (ESI): *m/z* [M]$^+$ calculated for $C_5H_3{}^{35}ClFNO_3{}^{32}S$: 210.95009 [M]$^+$, found 210.95007. The data are in agreement with those previously reported in the literature.[2]

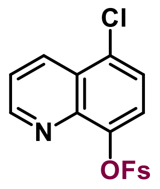

5-chloroquinolin-8-yl fluorosulfate: General procedure was followed using 5-chloroquinolin-8-ol (898 mg, 5 mmol). The title product was obtained after purification by column chromatography (30:1 → 20:1 → 10:1 Pentane/Et_2O) as a white solid. 1.15 g (87%). **R_f** = 0.54 (10:1 Pentane/Et_2O). **M.p.** = 102–103 °C. **^{1}H NMR** (400 MHz, $CDCl_3$) δ 9.10 (m, 1H), 8.63–8.58 (m, 1H), 7.71–7.64 (m, 3H). **^{13}C NMR** (101 MHz, $CDCl_3$) δ 152.3, 144.7, 140.9, 133.2, 132.0, 127.8, 125.7, 123.4, 121.2. **^{19}F NMR** (376 MHz, $CDCl_3$) δ 40.98. **MS** (70 eV, EI): *m/z* (%): 263 (16) [^{37}Cl-M$^+$], 261 (43) [^{35}Cl-M$^+$], 180 (19), 178 (59), 152 (32), 150 (100), 123 (17), 115 (13), 114 (12). **HRMS** (ESI): *m/z* [M+H]$^+$ calculated for $C_9H_6{}^{35}ClFNO_3{}^{32}S$: 261.97355, found 261.97433. The data are in agreement with those previously reported in the literature.[7]

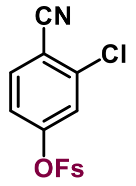

3-chloro-4-cyanophenyl fluorosulfate: General procedure was followed using 5-chloroquinolin-8-ol (768 mg, 5 mmol). The title product was obtained after purification by column chromatography (10:1 → 5:1 → 1:1 Pentane/Et_2O) as a white solid. 291 mg (25%). **R_f** = 0.30 (10:1 Pentane/Et_2O). **M.p.** = 42–43 °C. **^{1}H NMR** (600 MHz, $CDCl_3$) δ 7.84 (d, *J* = 8.7 Hz, 1H), 7.57 (d, *J* = 2.0 Hz, 1H), 7.42 (dd, *J* = 8.5, 1.8 Hz, 1H). **^{13}C NMR** (151 MHz, $CDCl_3$) δ 151.9, 139.2, 135.7, 123.2, 120.2, 114.4, 114.2. **^{19}F NMR** (564 MHz, $CDCl_3$) δ 40.06. **MS** (70 eV, EI): *m/z* (%): 237 (22) [^{37}Cl-M$^+$], 235 (60) [^{35}Cl-M$^+$], 154 (25), 152 (77), 126 (33), 125 (7), 124 (100), 64 (11), 63 (18). **HRMS** (ESI): *m/z* [M]$^+$ calculated for $C_7H_3{}^{35}ClFNO_3{}^{32}S$: 234.95007, found 234.95048.

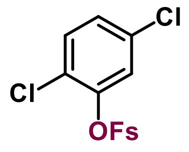

2,5-dichlorophenyl fluorosulfate: General procedure was followed using 2,5-dichlorophenol (494 mg, 2.5 mmol). The title product was obtained after purification by column chromatography (30:1 Pentane/Et_2O) as a colorless liquid. 494 mg (81%). **R_f** = 0.75 (10:1 Pentane/Et_2O). **^{1}H NMR** (600 MHz, $CDCl_3$) δ 7.48 (d, *J* = 8.7 Hz, 1H), 7.47 (m, 1H), 7.36 (dd, *J* = 8.7, 2.3 Hz, 1H). **^{13}C NMR** (151 MHz, $CDCl_3$) δ 145.7, 133.8, 132.0, 129.9, 125.6, 123.3. **^{19}F NMR** (376 MHz, $CDCl_3$) δ 41.54. **MS** (70 eV, EI): *m/z* (%): 246 (31) [^{37}Cl^{35}Cl-M$^+$], 244 (45) [^{35}Cl^{35}Cl-M$^+$], 163 (37), 161

(57), 137 (11), 135 (65), 133 (100), 82 (17), 73 (18), 63 (24), 62 (13). **HRMS** (ESI): *m/z* $[M]^+$ calculated for $C_6H_3{}^{35}Cl_2FO_3{}^{32}S$: 243.91585, found 243.91668.

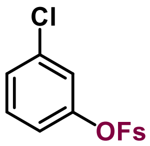

3-chlorophenyl fluorosulfate: General procedure was followed using 3-chlorophenol (643 mg, 5 mmol). The title product was obtained after purification by column chromatography (30:1 Pentane/Et_2O) as a colorless liquid. 874 mg (83%). R_f = 0.72 (10:1 Pentane/Et_2O). **^{1}H NMR** (400 MHz, $CDCl_3$) δ 7.43 (m, 2H), 7.38 (m, 1H), 7.27 (m, 1H). **^{13}C NMR** (101 MHz, $CDCl_3$) δ 149.9, 135.8, 131.1, 129.1, 121.7, 119.2. **^{19}F NMR** (376 MHz, $CDCl_3$) δ 38.18. **MS** (70 eV, EI): *m/z* (%): 211 (15) [^{37}Cl-M$^+$], 210 (51) [^{35}Cl-M$^+$], 129 (19), 127 (58), 101 (35), 99 (100), 65 (3), 64 (15), 63 (39), 62 (18), 61 (4). **HRMS** (ESI): m/z $[M]^+$ calculated for $C_6H_4{}^{35}ClFO_3{}^{32}S$: 209.95482, found 209.95527. The data are in agreement with those previously reported in the literature.[8]

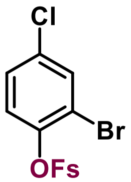

2-bromo-4-chlorophenyl fluorosulfate: General procedure was followed using 2-bromo-4-chlorophenol (1.04 g, 5 mmol). The title product was obtained after purification by column chromatography (50:1 Pentane/Et_2O) as a colorless liquid. 1.13 g (78%). R_f = 0.68 (10:1 Pentane/Et_2O). **^{1}H NMR** (600 MHz, $CDCl_3$) δ 7.71 (d, *J* = 2.2 Hz, 1H), 7.41 (dd, *J* = 8.8, 2.2 Hz, 1H), 7.38 (d, *J* = 8.8 Hz, 1H). **^{13}C NMR** (151 MHz, $CDCl_3$) δ 145.8, 135.2, 134.2, 129.3, 123.4, 116.4. **^{19}F NMR** (564 MHz, $CDCl_3$) δ 41.58. **MS** (70 eV, EI): *m/z* (%): 292 (15), 291 (4), 290 (49) [^{81}Br-M$^+$], 289 (2), 288 (36) [^{79}Br-M$^+$], 209 (22), 208 (7), 207 (100), 205 (78), 181 (19), 179 (78), 177 (59). **HRMS** (ESI): *m/z* $[M]^+$ calculated for $C_6H_3{}^{79}Br^{35}ClFO_3{}^{32}S$: 287.86533, found: 287.86557.

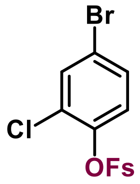

4-bromo-2-chlorophenyl fluorosulfate: General procedure was followed using 4-bromo-2-chlorophenol (1.04 g, 5 mmol). The title product was obtained after purification by column chromatography (30:1 Pentane/EtOAc) as a colorless liquid. 1.14 g (79%). R_f = 0.71 (10:1 Pentane/EtOAc). **^{1}H NMR** (600MHz, $CDCl_3$) δ 7.71 (d, *J* = 2.2 Hz, 1H), 7.51 (dd, *J* = 8.8, 2.2 Hz, 1H), 7.32 (d, *J* = 8.8 Hz, 1H). **^{13}C NMR** (151 MHz, $CDCl_3$) δ 144.9, 134.1, 131.6, 128.2, 123.9, 122.5. **^{19}F NMR** (564 MHz, $CDCl_3$) δ 41.18. **MS** (70 eV, EI): *m/z* (%): 293 (1), 292 (13), 291 (3), 290 (46) [^{81}Br-M$^+$], 289 (2), 288 (35) [^{79}Br-M$^+$], 209 (24), 208 (7), 207 (100), 205 (76), 181 (17), 180 (4), 179 (71), 178 (3), 177 (54). **HRMS** (ESI): *m/z* $[M]^+$ calculated for $C_6H_3{}^{79}Br^{35}ClFO_3{}^{32}S$: 287.86533, found: 287.86526.

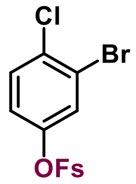

3-bromo-4-chlorophenyl fluorosulfate: General procedure was followed using 3-bromo-4-chlorophenol (1.04 g, 5 mmol). The title product was obtained after purification by column chromatography (Pentane) as a colorless liquid. 1.29 g (89%). R_f = 0.63 (Pentane). **^{1}H NMR** (600 MHz, $CDCl_3$) δ 7.64 (d, *J* = 2.4 Hz, 1H), 7.51 (d, *J* = 8.9, 1H), 7.29 (dd, *J* = 8.8 Hz, 2.6 Hz, 1H). **^{13}C NMR** (151 MHz, $CDCl_3$) δ 147.7, 135.4, 131.5, 126.4, 123.6, 121.1. **^{19}F NMR** (564 MHz, $CDCl_3$) δ 38.34. **MS** (70 eV, EI): m/z (%): 290 (62) [^{81}Br-M$^+$], 288 (45) [^{79}Br-M$^+$], 209 (24), 207 (93), 205 (70), 181 (23), 179 (100), 177 (77), 64 (9), 63 (86), 62 (42), 61 (21), 60 (4). **HRMS** (ESI): *m/z* $[M]^+$ calculated for $C_6H_3{}^{79}Br^{35}ClFO_3{}^{32}S$: 287.86533, found: 287.86499.

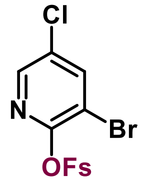

3-bromo-5-chloropyridin-2-yl fluorosulfate: General reaction procedure was followed using 3-bromo-5-chloropyridin-2-ol (1.04 g, 5 mmol). The reaction chamber was washed with DCM (3 x 5 mL) and the combined organic layers were washed with $NaHCO_3$ (2 x 20 mL) and with

sat. NaCl solution (1 x 20 mL). The organic layer was dried over Na_2SO_4 and solvent was evaporated under reduced pressure. The title product was obtained after purification by column chromatography (50:1 Pentane/Et_2O) as a colorless solid. 564 mg (38%). **R$_f$** = 0.48 (50:1 Pentane/EtOAc). **M.p.** = 29–30 °C. **^{1}H NMR** (400 MHz, $CDCl_3$) δ 8.28 (d, *J* = 2.3 Hz, 1H), 8.09 (d, *J* = 2.3 Hz, 1H). **^{13}C NMR** (101 MHz, $CDCl_3$) δ 151.3, 145.3, 143.6, 132.4, 110.2 (d, *J* = 3.4 Hz). **^{19}F NMR** (376 MHz, $CDCl_3$) δ 47.01. **MS** (70 eV, EI): *m/z* (%): 294 (1), 293 (20), 292 (5), 291 (72) [^{81}Br-M$^+$], 290 (4), 289 (53) [^{79}Br-M$^+$], 210 (10), 208 (32), 206 (25), 182 (24), 180 (100), 178 (77), 153 (23), 151 (18). The data are in agreement with those previously reported in the literature.[2]

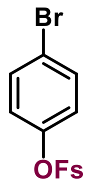

3-bromo-5-chlorophenyl fluorosulfate: General procedure was followed using 4-bromophenol (1.04 g, 5 mmol). The title product was obtained after purification by column chromatography (Pentane) as a colorless liquid. 990 mg (78%). **R$_f$** = 0.3 (Pentane). **^{1}H NMR** (600 MHz, $CDCl_3$) δ 7.61 (d, *J* = 9.0 Hz, 2H), 7.24 (d, *J* = 8.4 Hz, 2H). **^{13}C NMR** (151 MHz, $CDCl_3$) δ 148.9, 133.5, 122.6, 122.3. **^{19}F NMR** (564 MHz, $CDCl_3$) δ 37.77. **MS** (70 eV, EI): *m/z* (%): 256 (63) [^{81}Br-M$^+$], 254 (62) [^{79}Br-M$^+$], 173 (97), 171 (100), 145 (83), 143 (86), 64 (52), 63 (82), 62 (27), 61 (12). The data are in agreement with those previously reported in the literature.[9]

C–Br Site-selective Aryl- and Alkylation Products

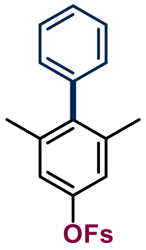

2,6-dimethyl-[1,1'-biphenyl]-4-yl fluorosulfate (2): General procedure A. The title product was obtained after purification by column chromatography (Pentane) as a colorless oil. 38 mg (77 %). **R$_f$** = 0.43 (Pentane). **^{1}H NMR** (600 MHz, $CDCl_3$) δ 7.45 (dd, *J* = 7.5, 7.5 Hz, 2H), 7.38 (dd, *J* = 7.4, 7.4 Hz, 1H), 7.11 (d, *J* = 7.1 Hz, 2H), 7.08 (s, 2H), 2.06 (s, 6H). **^{13}C NMR** (151 MHz, $CDCl_3$) δ 148.7, 142.4, 139.3, 139.0, 128.73, 128.68, 127.3, 119.1, 21.0. **^{19}F NMR** (564 MHz, $CDCl_3$) δ 37.63. **MS** (70 eV, EI): *m/z* (%): 282 (6), 281 (16), 280 (100) [M$^+$], 198 (11), 197 (70), 169 (41), 165 (17), 154 (32), 153 (25), 152 (22). **HRMS** (ESI): *m/z* [M]$^+$ calculated for $C_{14}H_{13}O_3F^{32}S$: 280.05640, found 280.05570.

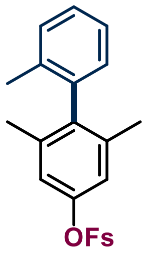

2,2',6-trimethyl-[1,1'-biphenyl]-4-yl fluorosulfate (3): General procedure A. The title product was obtained after purification by column chromatography (Pentane) as a white solid. 50 mg (66 %). **R$_f$** = 0.59 (Pentane). **M.p.** = 67–68 °C. **^{1}H NMR** (600 MHz, $CDCl_3$) δ 7.30–7.27 (m, 3H), 7.09 (s, 2H), 6.97 (d, *J* = 6.9 Hz, 1H), 1.99 (s, 6H), 1.97 (s, 3H). **^{13}C NMR** (151 MHz, $CDCl_3$) δ 148.7, 141.6, 138.9, 138.6, 135.4, 130.3, 128.5, 127.7, 126.4, 119.1, 20.5, 19.3. **^{19}F NMR** (564 MHz, $CDCl_3$) δ 37.57. **MS** (70 eV, EI): *m/z* (%) 296 (6), 295 (17), 294 (100) [M$^+$], 280 (4), 279 (23), 212 (6), 211 (36), 183 (19), 179 (36), 178 (12), 168 (20), 167 (15), 165 (27). **HRMS** (ESI) *m/z* [M]$^+$ calculated for $C_{15}H_{15}O_3F^{32}S$: 294.07205, found 294.07135.

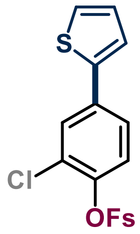

2-chloro-4-(thiophen-2-yl)phenyl fluorosulfate (4): General procedure A. The title product was obtained after purification by column chromatography (Pentane→50:1 Pentane/EtOAc) as a yellow solid. 462 mg (79%). **R$_f$** = 0.27 (Pentane). **M.p.** = 56–57 °C. **^{1}H NMR** (600 MHz, $CDCl_3$) δ 7.74 (d, *J* = 2.2 Hz, 1H), 7.56 (dd, *J* = 8.6, 2.2 Hz, 1H), 7.43 (dd, *J* = 8.6, 1.1 Hz, 1H), 7.38 (dd, *J* = 5.1, 1.1 Hz, 1H), 7.34 (dd, *J* = 3.7, 1.1 Hz, 1H), 7.12 (dd, *J* = 5.0, 3.7 Hz, 1H). **^{13}C NMR** (151 MHz, $CDCl_3$) δ 144.6, 140.6, 136.3, 128.5, 128.2, 127.4, 126.8, 125.5, 125.0, 123.1. **^{19}F NMR** (564 MHz,

$CDCl_3$) δ 40.96. **MS** (70 eV, EI): *m/z* (%): 294 (18) [^{37}Cl-M^+], 292 (42) [^{35}Cl-M^+], 211 (37), 209 (100). 183 (11), 181 (32), 148 (1), 147 (2), 145 (11). **HRMS** (ESI): *m/z* $[M]^+$ calculated for $C_{10}H_6O_3{}^{35}ClF^{32}S_2$: 291.94255, found 291.94256.

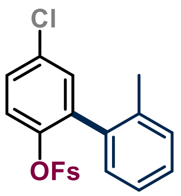

5-chloro-2-(o-tolyl)phenyl fluorosulfate (5): General procedure A. The title product was obtained after purification by column chromatography (Pentane) as a colorless oil. 48.2 mg (80%). **R$_f$** = 0.55 (Pentane). **^{1}H NMR** (600 MHz, $CDCl_3$) δ 7.45 (dd, *J* = 8.8, 2.5 Hz, 1H), 7.40 (d, *J* = 8.8 Hz, 1H), 7.37 (d, *J* = 2.5 Hz, 1H), 7.35 (dd, *J* = 7.5, 7.5 Hz, 1H), 7.30 (d, *J* = 7.5 Hz, 1H), 7.27 (dd, *J* = 7.4, 7.4 Hz, 1H), 7.18 (d, *J* = 7.6 Hz, 1H)) 2.17 (s, 3H). **^{13}C NMR** (151 MHz, $CDCl_3$) δ 145.9, 136.8, 136.1, 134.2, 133.6, 132.2, 130.3, 129.7, 129.2, 129.0, 125.9, 122.6, 19.8. **^{19}F NMR** (564 MHz, $CDCl_3$) δ 40.33. **MS** (70 eV, EI): *m/z* (%): 303 (4), 302 (26) [^{37}Cl-M^+],, 301 (11), 300 (69) [^{35}Cl-M^+], 220 (1), 219 (14), 217 (41), 202 (18), 183 (15), 182 (100), 181 (31), 153 (14), 152 (23). **HRMS** (ESI): *m/z* $[M+Na]^+$ calculated for $C_{13}H_{10}O_3{}^{35}ClF^{32}SNa$: 322.99154, found 322.99136.

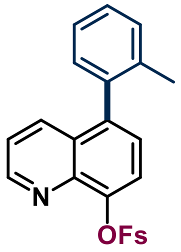

5-(o-tolyl)quinolin-8-yl fluorosulfate (6): General procedure A. The title product was obtained after purification by column chromatography (100:5 Pentane/Et_3N) as a colorless oil. 43 mg (67%). **R$_f$** = 0.4 (100:5 Pentane/Et_3N) **^{1}H NMR** (400 MHz, $CDCl_3$) δ 9.05 (dd, *J* = 4.1, 1.6 Hz, 1H), 7.84 (dd, *J* = 8.6, 1.6 Hz, 1H), 7.80 (d, *J* = 7.9 Hz, 1H), 7.47–7.43 (m, 2H), 7.41–7.39 (m, 1H), 7.37–7.35 (m, 1H), 7.33 (dd, *J* = 7.5, 7.5 Hz, 1H), 7.21 (d, *J* = 7.5 Hz, 1H), 2.01 (s, 3H). **^{13}C NMR** (151 MHz, $CDCl_3$) δ 151.5, 145.0, 141.3, 140.3, 137.2, 136.5, 134.6, 130.3, 130.2, 128.8, 128.6, 126.3, 125.9, 122.6, 120.7, 20.0. **^{19}F NMR** (376 MHz, $CDCl_3$) δ 40.74. **MS** (70 eV, EI): *m/z* (%): 318 (15), 317 (73) [M^+], 235 (18), 234 (100), 207 (21), 206 (70), 205 (28), 204 (62), 178 (16). **HRMS** (ESI): *m/z* $[M]^+$ calculated for $C_{16}H_{12}O_3NF^{32}S$: 317.05165, found 317.05188.

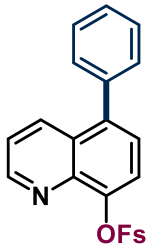

5-phenyl-quinolin-8-yl fluorosulfate (7): General procedure B. The title product was obtained after purification by column chromatography (100% Pentane) as a white solid. 48.2 mg (80 %). **R$_f$** = 0.32 (20:1 Pentane/EtOAc). **M.p.** = 114–115 °C. **^{1}H NMR** (600 MHz, $CDCl_3$) δ 9.07 (dd, *J* = 4.1, 1.5 Hz, 1H), 8.27 (dd, *J* = 8.6, 1.5 Hz, 1H), 7.79 (d, *J* = 7.9 Hz, 1H), 7.54 (m, 3H), 7.50 (m, 2H), 7.44 (m, 2H). **^{13}C NMR** (151 MHz, $CDCl_3$) δ 151.5, 145.1, 141.7, 140.5, 137.9, 134.7, 129.9, 128.7, 128.3, 126.3, 122.5, 120.7. **^{19}F NMR** (564 MHz, $CDCl_3$) δ 40.84. **MS** (70 eV, EI): *m/z* (%): 304 (9), 303 (50) [M^+], 221 (13), 220 (68), 193 (15), 192 (100), 191 (27), 190 (12). **HRMS** (ESI): *m/z* $[M]^+$ calculated for $C_{15}H_{10}O_3NF^{32}S$: 303.03599, found 303.03587.

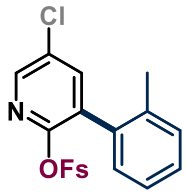

5-chloro-3-(o-tolyl)pyridin-2-yl fluorosulfate (8): General procedure B. The title product was obtained after purification by column chromatography (Pentane→10:1 Pentane/EtOAc) as a colorless oil. 49 mg (82%). **R$_f$** = 0.27 (50:1 Pentane/EtOAc). **^{1}H NMR** (600 MHz, $CDCl_3$) δ 8.35 (d, *J* = 2.6 Hz, 1H), 7.77 (d, *J* = 2.6 Hz, 1H), 7.38 (dd, *J* = 7.4, 7.4 Hz, 1H), 7.33–7.28 (m, 2H), 7.16 (d, *J* = 7.6 Hz, 1H), 2.18 (s, 3H). **^{13}C NMR** (151 MHz, $CDCl_3$) δ 151.8, 145.7, 141.8, 136.0, 131.9, 131.7, 130.6, 129.6, 129.5, 126.2, 19.7. **^{19}F NMR** (564 MHz, $CDCl_3$) δ 46.57. **MS** (70 eV, EI): *m/z* (%): 303 (26) [^{37}Cl-M^+], 301 (65) [^{35}Cl-M^+], 220 (21), 218 (60), 207 (9), 205 (6), 204 (10), 203 (22), 202 (27), 201 (14), 200 (11), 167 (15), 166 (100), 155 (34) 154 (48), 140

(11), 139 (14), 128 (27), 127 (35), 126 (13). **HRMS** (ESI): *m/z* $[M+Na]^+$ calculated for $C_{12}H_9O_3N^{35}ClF^{32}SNa$: 323.98679, found 323.98700.

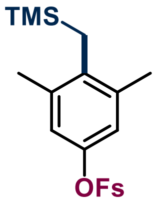

3,5-dimethyl-4-((trimethylsilyl)methyl)phenyl fluorosulfate (11): General procedure B. The title product was obtained after purification by column chromatography (Pentane) as a colorless oil. 50 mg (86 %). $\mathbf{R_f}$ = 0.59 (Pentane). $\mathbf{^1H}$ **NMR** (600 MHz, $CDCl_3$) δ 6.95 (s, 2H), 2.26 (s, 6H), 2.15 (s, 2H), 0.04 (s, 9H). $\mathbf{^{13}C}$ **NMR** (151 MHz, $CDCl_3$) δ 146.2, 139.3, 137.0, 119.4, 21.3, 20.2, -0.1. $\mathbf{^{19}F}$ **NMR** (564 MHz, $CDCl_3$) δ 36.97. **MS** (70 eV, EI): *m/z* (%): 291 (1), 290 (6) $[M^+]$, 276 (3), 275 (16), 218 (1), 217 (12), 208 (2), 207 (15), 134 (49), 118 (70), 117 (39), 106 (60), 91 (50), 73 (100) $[Me_3Si^+]$. **HRMS** (ESI): *m/z* $[M]^+$ calculated for $C_{18}H_{13}O_3SiF^{32}S$: 290.07958, found 290.07970.

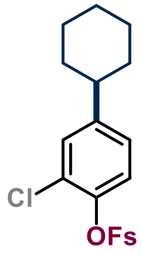

2-chloro-4-cyclohexylphenyl fluorosulfate (12): General procedure B. The title product was obtained after purification by column chromatography (Pentane) as a colorless oil. 41.0 mg (70%). $\mathbf{R_f}$ = 0.6 (Pentane). $\mathbf{^1H}$ **NMR** (600 MHz, $CDCl_3$) δ 7.35 (s, 1H), 7.33 (d, *J* = 8.5 Hz, 1H), 7.18 (dd, *J* = 8.6, 2.1 Hz, 1H), 2.52 (m, 1H), 1.86 (m, 4H), 1.76 (m, 1H), 1.42–1.36 (m, 4H), 1.25 (m, 1H). $\mathbf{^{13}C}$ **NMR** (151 MHz, $CDCl_3$) δ 150.2, 143.7, 129.6, 126.8, 126.4, 122.3, 43.9, 34.1, 26.5, 25.8. $\mathbf{^{19}F}$ **NMR** (564 MHz, $CDCl_3$) δ 40.48. **MS** (70 eV, EI): *m/z* (%): 295 (4), 294 (24) $[^{37}Cl\text{-}M^+]$, 293 (9), 292 (63) $[^{35}Cl\text{-}M^+]$, 238 (38), 237 (10), 236 (100), 223 (18), 189 (10), 155 (23), 153 (70), 149 (10), 141 (11), 127 (23), 125 (23), 115 (21), 103 (21), 99 (16) 89 (20). **HRMS** (ESI): *m/z* $[M\text{-}^{35}Cl]^+$ calculated for $C_{12}H_{14}O_3F^{32}S$: 257.06422, found 257.06470.

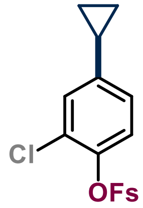

2-chloro-4-cyclopropylphenyl fluorosulfate (13): General procedure B. The title product was obtained after purification by column chromatography (Pentane) as colorless oil. 46.9 mg (94%). $\mathbf{R_f}$ = 0.46 (Pentane). $\mathbf{^1H}$ **NMR** (600 MHz, $CDCl_3$) δ 7.29 (d, *J* = 8.6 Hz, 1H), 7.19 (d, *J* = 2.2 Hz, 1H), 7.03 (dd, *J* = 8.6, 2.2 Hz, 1H), 1.91 (m, 1H), 1.05 (m, 2H), 0.72 (m, 2H). $\mathbf{^{13}C}$ **NMR** (151 MHz, $CDCl_3$) δ 146.7, 143.4, 128.3, 126.5, 125.5, 122.3, 15.0, 10.0. $\mathbf{^{19}F}$ **NMR** (564 MHz, $CDCl_3$) δ 40.40. **MS** (70 eV, EI): *m/z* (%): 253 (2), 252 (14) $[^{37}Cl\text{-}M^+]$, 251 (4), 250 (35) $[^{35}Cl\text{-}M^+]$, 169 (30), 168 (10), 167 (100), 165 (1), 105 (2), 104 (19), 103 (80), 102 (10), 78 (10), 77 (41). **HRMS** (ESI): *m/z* $[M]^+$calculated for $C_9H_8O3^{35}ClF^{32}S$: 249.98612, found 249.98638.

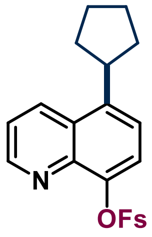

5-cyclopentyl-quinolin-8-yl fluorosulfate (14): General procedure B (Organozinc 1.5 equiv.). The title product was obtained after purification by column chromatography (Pentane → 80:1 → 50:1 → 20:1) as a colorless oil. 46 mg (78%). $\mathbf{R_f}$ = 0.1 (50:1 Pentane/EtOAc). $\mathbf{^1H}$ **NMR** (600 MHz, $CDCl_3$) δ 9.03 (dd, *J* = 4.0, 1.4 Hz, 1H), 8.52 (d, *J* = 8.7, 1.3 Hz, 1H), 7.67 (d, *J* = 8.0 Hz, 1H), 7.54 (dd, *J* = 8.6, 4.0 Hz, 1H) 7.49 (d, *J* = 8.1 Hz, 1H), 3.71 (m, 1H), 2.19 (m, 2H), .1.89 (m, 2H), 1.82–1.73 (m, 4H). $\mathbf{^{13}C}$ **NMR** (151 MHz, $CDCl_3$) δ 151.0, 144.3, 144.1, 140.6, 132.5, 128.7, 122.0, 121.7, 120.8, 40.8, 33.7, 25.2. $\mathbf{^{19}F}$ **NMR** (564 MHz, $CDCl_3$) δ 40.48. **MS** (70 eV, EI): *m/z* (%): 297 (6), 296 (18), 295 (100) $[M^+]$, 214 (1), 213 (14), 212 (79), 196 (19), 184 (42), 183 (13), 170 (42), 167 (12), 156 (11), 155 (11), 154 (32), 146 (27), 145 (10), 144 (89), 142 (37), 141 (22), 117 (12), 116 (82), 115 (20). **HRMS** (ESI): *m/z* $[M+H]^+$ calculated for $C_{14}H_{14}O_3N\ ^{35}ClF^{32}S$: 295.06730, found 295.06717.

C–Br Site-selective Thiolation Products

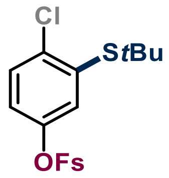

3-(tert-butylthio)-4-chlorophenyl fluorosulfate (9): General procedure D was followed with stirring for 6 hours. The title product was obtained after purification by column chromatography (Pentane) as a colorless oil. 53 mg (89%). $\mathbf{R_f}$ = 0.3 (Pentane). **^{1}H NMR** (400 MHz, $CDCl_3$) δ 7.64 (d, *J* = 2.9 Hz, 1H), 7.59 (d, *J* = 8.8 Hz, 1H), 7.28 (dd, *J* = 8.8, 2.9 Hz, 1H), 1.37 (s, 9H). **^{13}C NMR** (151 MHz, $CDCl_3$) δ 147.3, 141.1, 135.4, 131.4, 131.1, 122.2, 49.5, 31.0. **^{19}F NMR** (564 MHz, $CDCl_3$) δ 37.86. **MS** (70 eV, EI): *m/z* (%): 300 (2) [^{37}Cl-M$^+$], 298 (5) [^{35}Cl-M$^+$], 285 (1), 283 (3), 244 (9), 242 (22), 95 (14), 57 (100). **HRMS** (ESI): *m/z* [M]$^+$ calculated for $C_{10}H_{12}O_3{}^{35}ClFS_2$: 297.98950, found 297.98963.

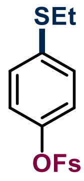

4-(ethylthio)-phenyl fluorosulfate (10): General procedure D was followed with stirring for 8 hours. The title product was obtained after purification by column chromatography (100:1 Pentane/EtOAc) as a yellow oil. 41 mg (87%). $\mathbf{R_f}$ = 0.2 (100:1 Pentane/EtOAc). **^{1}H NMR** (600 MHz, $CDCl_3$) δ 7.36 (d, *J* = 8.9 Hz, 2H), 7.25 (d, *J* = 9.0 Hz, 2H), 2.97 (q, *J* = 7.3 Hz, 2H), 1.34 (t, *J* = 7.4 Hz, 3H). **^{13}C NMR** (151 MHz, $CDCl_3$) δ 147.7, 138.5, 129.7, 121.3, 27.4, 14.1. **^{19}F NMR** (564 MHz, $CDCl_3$) δ 37.36. **MS** (70 eV, EI): *m/z* (%): 236 (54) [M$^+$], 154 (10), 152 (100), 125 (54), 124 (13), 97 (22). **HRMS** (ESI): *m/z* [M]$^+$ calculated for $C_8H_9O_3FS_2$: 235.99717, found 235.99786.

C–OFs Site-selective Aryl- and Alkylation Products

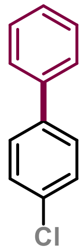

4-chloro-1,1'-biphenyl (15): General procedure C. The title product was obtained after purification by column chromatography (Pentane) as a white solid. 34 mg (90%). **M.p.** = 73–75 °C. $\mathbf{R_f}$ = 0.82 (Pentane). **^{1}H NMR** (600 MHz, $CDCl_3$) δ 7.56 (d, *J* = 7.3 Hz, 2H), 7.52 (d, *J* = 8.4 Hz, 2H), 7.45 (dd, *J* = 7.6, 7.6 Hz, 2H), 7.41 (d, *J* = 8.4 Hz, 2H), 7.37 (dd, *J* = 7.4, 7.4 Hz, 1H). **^{13}C NMR** (151 MHz, $CDCl_3$) δ 140.0, 139.6, 133.3, 128.88, 128.86, 128.4, 127.6, 127.0. **MS** (70 eV, EI): *m/z* (%): 191 (4), 190 (33) [^{37}Cl-M$^+$], 189 (13), 188 (100) [^{35}Cl-M$^+$], 153 (16), 152 (47), 151(14), 76 (11), 75 (5). The data are in agreement with those previously reported in the literature.[5]

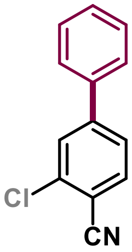

3-chloro-[1,1'-biphenyl]-4-carbonitrile (16): General procedure C (Organozinc 1.2 equiv.). The title product was obtained after purification by column chromatography (20:1 Pentane/EtOAc) as a white solid. 36 mg (84%). **M.p.** = 97–98 °C. $\mathbf{R_f}$ = 0.45 (20:1 Pentane/EtOAc). **^{1}H NMR** (600 MHz, $CDCl_3$) δ 7.74–7.72 (m, 2H), 7.59–7.56 (m, 3H), 7.51–7.45 (m, 3H). **^{13}C NMR** (151 MHz, $CDCl_3$) δ 147.1, 137.9, 137.2, 134.2, 129.2, 128.4, 127.2, 125.7, 116.1, 111.6. **MS** (70 eV, EI): *m/z* (%): 216 (4), 215 (31) [^{37}Cl-M$^+$], 214 (15), 213 (100) [^{35}Cl-M$^+$], 212 (1), 179 (2), 178 (13), 177 (25), 176 (6), 152 (5), 151 (21), 150 (12), 149 (4), 148 (1). The data are in agreement with those previously reported in the literature.[5]

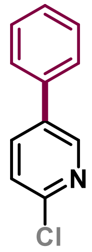

2-chloro-5-phenylpyridine (17): General procedure C (Organozinc 1.2 equiv.). The title product was obtained after purification by column chromatography (Pentane→50:1 Pentane/EtOAc) as a white solid. 29 mg (77%). $\mathbf{R_f}$ = 0.13 (Pentane). **M.p.** = 49–50 °C. **^{1}H NMR** (600 MHz, $CDCl_3$) δ 8.60 (s, 1H), 7.84 (dd, *J* = 8.3, 2.5 Hz, 1H), 7.55 (d, *J* = 7.3 Hz, 2H), 7.48 (dd, *J* = 7.5, 7.5 Hz, 2H), 7.43–7.39 (m, 2H). **^{13}C NMR** (151 MHz, $CDCl_3$) δ 150.3, 147.9, 137.1, 136.4, 135.6, 129.2, 128.4, 127.0, 124.2. **MS**

(70 eV, EI): *m/z* (%): 192 (4), 191 (33) [^{37}Cl-M$^+$], 190 (15), 189 (100) [^{35}Cl-M$^+$], 188 (8), 155 (4), 154 (30), 153 (10), 152 (6), 151 (1), 129 (1), 128 (12), 127 (28), 126 (15), 125 (3). The data are in agreement with those previously reported in the literature.[5]

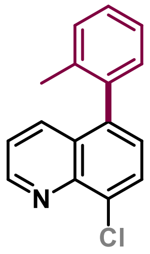

5-chloro-8-(o-tolyl)quinoline (18): General procedure C. The title product was obtained after purification by column chromatography (Pentane) as a yellow oil. 45 mg (88%). **R_f** = 0.42 (100:1 Pentane/EtOAc). **^{1}H NMR** (600 MHz, $CDCl_3$) δ 8.96 (dd, *J* = 4.0, 1.6 Hz, 1H), 8.65 (dd, *J* = 8.6, 1.6 Hz, 1H), 7.70 (d, *J* = 7.8 Hz, 1H), 7.53 (d, *J* = 7.7 Hz, 1H), 7.51 (dd, *J* = 8.6, 4.1 Hz, 1H), 7.38–7.27 (m, 4H), 2.05 (s, 3H). **^{13}C NMR** (151 MHz, $CDCl_3$) δ 150.9, 147.1, 140.8, 139.1, 136.9, 132.9, 130.6, 130.2, 130.1, 129.8, 128.0, 126.4, 126.3, 125.5, 121.7, 20.5. **MS** (70 eV, EI): *m/z* (%): 256 (2), 255 (12), 254 (21) [^{37}Cl-M$^+$], 253 (37), 252 (49) [^{35}Cl-M$^+$], 251 (7), 250 (2), 241 (5), 240 (33), 239 (18), 238 (100), 219 (5), 218 (27), 217 (19), 216 (17), 215 (8), 214 (4). The data are in agreement with those previously reported in the literature.[5]

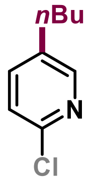

5-butyl-2-chloropyridine (19): General procedure C (Organozinc 1.5 equiv.). The title product was obtained after purification by column chromatography (Pentane→50:1 Pentane/EtOAc) as a colorless oil. 30 mg (88%). **R_f** = 0.57 (50:1 Pentane/EtOAc). **^{1}H NMR** (400 MHz, $CDCl_3$) δ 8.20 (d, *J* = 2.0 Hz, 1H), 7.45 (dd, *J* = 8.1, 2.5 Hz, 1H), 7.22 (d, *J* = 8.2 Hz, 1H), 2.58 (t, *J* = 7.6 Hz, 2H), 1.58 (m, 2H), 1.35 (m, 2H), 0.93 (t, *J* = 7.3 Hz, 3H). **^{13}C NMR** (151 MHz, $CDCl_3$) δ 142.5, 141.8, 131.7, 129.8, 116.8, 26.1, 24.9, 15.1, 6.8. **MS** (70 eV, EI): *m/z* (%): 172 (1), 171 (13) [^{37}Cl-M$^+$], 170 (4), 169 (39) [^{35}Cl-M$^+$], 130 (1), 129 (15), 128 (35), 127 (44), 126 (100). The data are in agreement with those previously reported in the literature.[5]

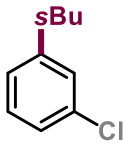

1-(sec-butyl)-3-chlorobenzene (20): General procedure C. Organozinc was prepared from commercial *sec*-butylmagnesium LiCl complex. The title product was obtained after purification by column chromatography (Pentane) as a colorless oil. 28 mg (84 %). **R_f** = 0.82 (Pentane). **^{1}H NMR** (600 MHz, $CDCl_3$) δ 7.21 (dd, *J* = 7.6, 7.6 Hz, 1H), 7.16 (d, *J* = 8.8 Hz, 2H), 7.06 (d, *J* = 7.6 Hz, 1H), 2.58 (m, 1H), 1.59 (n, 2H), 1.23 (d, *J* =7.0 Hz, 3H), 0.82 (t, *J* = 7.4 Hz, 3H). **^{13}C NMR** (151 MHz, $CDCl_3$) δ 149.7, 134.0, 129.5, 127.2, 125.9, 125.3, 41.5, 31.0, 21.7, 12.2. **MS** (70 eV, EI): *m/z* (%): 170 (8) [^{37}Cl-M$^+$], 169 (3), 168 (25) [^{35}Cl-M$^+$], 142 (3), 141 (33), 140 (10), 139 (100), 127 (5), 126 (1), 125 (15), 105 (2), 104 (8), 103 (48), 102 (6), 101 (3). The data are in agreement with those previously reported in the literature.[5]

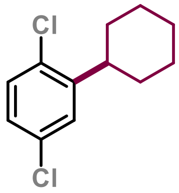

1,4-dichloro-2-cyclohexylbenzene (21): General procedure C. The title product was obtained after purification by column chromatography (Pentane) as a colorless oil. 31 mg (69%). **R_f** = 0.93 (Pentane). **^{1}H NMR** (600 MHz, $CDCl_3$) δ 7.26 (d, *J* = 8.4 Hz, 1H), 7.23 (dd, *J* = 2.4 Hz, 1H), 7.08 (dd, *J* = 8.4, 2.5 Hz, 1H), 2.96 (m, 1H), 1.86 (m, 4H), 1.47–1.23 (m, 6H). **^{13}C NMR** (151 MHz, $CDCl_3$) δ 146.5, 132.7, 131.7, 130.4, 127.4, 126.8, 40.6, 32.9, 26.7, 26.1. **MS** (70 eV, EI): *m/z* (%): 233 (1), 232 (5) [^{37}Cl^{37}Cl-M$^+$], 231 (4), 230 (51) [^{37}Cl^{35}Cl-M$^+$], 229 (6), 228 (45) [^{35}Cl^{35}Cl-M$^+$], 177 (1), 175 (11), 175 (7), 174 (65), 173 (10), 172 (100), 171 (1), 162 (6), 161 (18), 160 (7), 159 (27). The data are in agreement with those previously reported in the literature.[5]

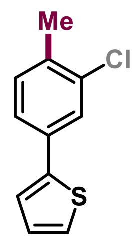

2-(3-chloro-4-methylphenyl)thiophene (22): General procedure C (Organozinc 1.5 equiv.). The title product was obtained after purification by column chromatography (Pentane) as a white solid. 32 mg (76%). **R_f** = 0.74 (Pentane). **^{1}H NMR** (600 MHz, $CDCl_3$) δ 7.60 (s, 1H), 7.39 (d, *J* = 7.9 Hz, 1H), 7.28 (d, *J* = 4.4 Hz, 2H), 7.22 (d, *J* = 7.9 Hz, 1H), 7.07 (dd, *J* = 4.3 Hz, 1H), 2.38 (s, 3H). **^{13}C NMR** (151 MHz, $CDCl_3$) δ 143.0, 135.2, 134.9, 133.8, 131.4, 128.2, 126.4, 125.2, 124.3, 123.4, 19.9. **MS** (70 eV, EI): *m/z* (%): 212 (2), 211 (5), 210 (38) [^{37}Cl-M$^+$], 209 (20), 208 (100) [^{35}Cl-M$^+$], 207 (21), 176 (1), 175 (5), 174 (9), 173 (68), 172 (10), 171 (26), 170 (2), 130 (1), 129 (9), 128 (13), 127 (10), 126 (4), 125 (1). The data are in agreement with those previously reported in the literature.[5]

Modular Sequential Coupling Products

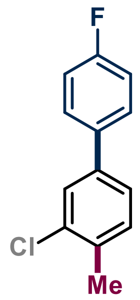

3-chloro-4'-fluoro-4-methyl-1,1'-biphenyl (23): General procedure E. The title product was obtained after purification by column chromatography (Pentane) as a colorless oil. 43 mg (97%). **R_f** = 0.74 (Pentane). **^{1}H NMR** (600 MHz, $CDCl_3$) δ 7.51 (m, 3H), 7.33 (dd, *J* = 7.8, 2.0 Hz, 1H), 7.28 (d, *J* = 7.8 Hz, 1H), 7.12 (m, 2H), 2.41 (s, 3H). **^{13}C NMR** (151 MHz, $CDCl_3$) δ 162.5 (d, *J* = 246.7 Hz), 139.4, 135.9 (d, *J* = 3.8 Hz), 134.9, 134.8, 131.3, 128.5 (d, *J* = 8.2 Hz), 127.4, 125.1, 115.7 (d, *J* = 21.5 Hz), 19.7. **^{19}F NMR** (564 MHz, $CDCl_3$) δ -115.24 (m, 1F). **MS** (70 eV, EI): *m/z* (%): 223 (4), 222 (34) [^{37}Cl-M$^+$], 221(18), 220 (100) [^{35}Cl-M$^+$], 219 (13), 216 (1), 186 (13), 185 (91), 184 (19), 183 (68), 182 (8), 181 (7), 180 (1), 179 (15), 165 (30). **HRMS** (ESI): *m/z* [M]$^+$ calculated for $C_{12}H_{14}O_3{}^{35}ClFS$: 286.99395, found 286.99262.

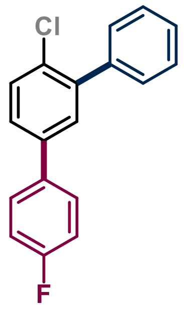

4'-chloro-4-fluoro-1,1':3',1''-terphenyl (24): General procedure E. The title product was obtained after purification by column chromatography (Pentane) as a colorless oil. 53 mg (94%). On 1 mmol scale: 226 mg, (80%). **R_f** = 0.53 (Pentane). **^{1}H NMR** (600 MHz, $CDCl_3$) δ 7.42 (d, *J* = 2.2 Hz, 1H), 7.38 (dd, *J* = 8.2, 2.2 Hz, 1H), 7.33 (d, *J* = 8.2 Hz, 1H), 7.24 (m, 3H), 7.10 (m, 2H), 7.06 (m, 2H), 6.91 (m, 2H). **^{13}C NMR** (151 MHz, $CDCl_3$) δ 161.8 (d, *J* = 246.7 Hz), 142.1, 140.0, 137.9, 136.3 (d, *J* = 3.1 Hz), 133.3, 131.7, 131.3 (d, *J* = 7.9 Hz), 130.4, 129.6, 128.1, 127.5, 127.1, 114.9 (d, *J* = 21.5 Hz). **^{19}F NMR** (564 MHz, CDCl3) δ -115.69 (m, 1F). **MS** (70 eV, E I): *m/z* (%): 285 (6), 284 (33) [^{37}Cl-M$^+$], 283 (22), 282 (100) [^{35}Cl-M$^+$], 281 (9), 280 (3), 249 (4), 248 (11), 247 (66), 246 (85), 245 (26), 244 (46), 243 (5), 242 (6), 228 (7), 227 (28), 226 (25), 224 (6), 220 (16), 219 (5), 218 (8), 123 (16), 122 (19). **HRMS** (ESI): *m/z* [M]$^+$ calculated for $C_{18}H_{12}{}^{35}ClF$: 282.06061, found 282.06054.

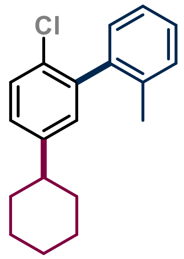

2-chloro-5-cyclohexyl-2'-methyl-1,1'-biphenyl (25): General procedure E. The title product was obtained after purification by column chromatography (Pentane) as a colorless oil. 56 mg (98%). **R_f** = 0.55 (Pentane). **^{1}H NMR** (600 MHz, $CDCl_3$) 7.39 (d, *J* = 8.3 Hz, 1H), 7.33–7.25 (m, 3H), 7.18 (d, *J* = 7.3 Hz, 1H), 7.16 (dd, *J* = 8.3, 2.3 Hz, 1H), 7.10 (d, *J* = 2.3 Hz, 1H), 2.52 (m, 1H), 2.16 (s, 3H), 1.93–1.86 (m, 4H), 1.77 (m, 1H), 1.46–1.37 (m, 4H), 1.25 (m, 1H). **^{13}C NMR** (151 MHz, $CDCl_3$) δ 139.6, 133.2, 132.7, 129.2, 123.3, 122.7, 122.5, 122.4, 122.0, 120.7, 120.0, 118.4, 36.9, 27.5, 27.4, 19.8, 19.0, 12.9. **MS** (70 eV, E I): *m/z* (%): 287 (7), 286 (34) [^{37}Cl-M$^+$], 285 (21), 284 (100) [^{35}Cl-M$^+$], 249 (7), 243 (8), 241 (23), 230 (14), 228 (41), 217 (7), 215 (24), 213 (13), 207 (15), 206 (39), 205 (12), 204 (8), 203 (12), 202 (18), 201 (7), 194 (4), 193 (18), 192

(16), 191 (29), 190 (16), 189 (22), 181 (29), 180 (8), 179 (40), 178 (73), 167 (14), 166 (24), 165 (73), 164 (9). **HRMS** (ESI): *m/z* $[M]^+$ calculated for $C_{19}H_{21}{}^{35}Cl$: 284.13263, found 284.13266.

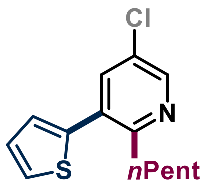

5-chloro-2-pentyl-3-(thiophen-2-yl)pyridine (26): General procedure E. The title product was obtained after purification by column chromatography (Pentane) as a yellowish oil. 37.4 mg (70%). R_f = 0.54 (50:1 Pentane/EtOAc). **^{1}H NMR** (600 MHz, $CDCl_3$) δ 8.48 (d, *J* = 2.4 Hz, 1H), 7.64 (d, *J* = 2.4 Hz, 1H), 7.42 (d, *J* = 5.1, 1.2 Hz, 1H), 7.11 (d, *J* = 5.1, 3.5 Hz, 1H), 7.08 (d, *J* = 3.5, 1.2 Hz, 1H), 2.87 (m, 2H), 1.67 (m, 2H), 1.29 (m, 4H), 0.85 (m, 3H). **^{13}C NMR** (151 MHz, $CDCl_3$) δ 158.7, 147.1, 139.2, 137.6, 130.7, 128.7, 127.5, 127.4, 126.6, 35.3, 31.7, 29.5, 22.4, 14.0. **MS** (70 eV, EI): *m/z* (%): 267 (2) $[M^{+}\text{-}^{37}Cl]$, 265 (5) $[M^{+}\text{-}^{35}Cl]$, 264 (4), 238 (4), 236 (11), 223 (10), 222 (28), 212 (6), 211 (37), 210 (43), 209 (100), 208 (84), 207 (6), 173 (19), 172 (11). **HRMS** (ESI): *m/z* $[M+H]^+$ calculated for $C_{14}H_{27}{}^{35}ClNS$: 266.07647, found 266.07648.

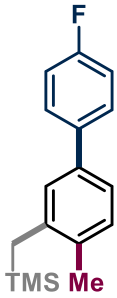

((4'-fluoro-4-methyl-[1,1'-biphenyl]-3-yl)methyl)trimethylsilane (27): General procedure E. The title product was obtained after purification by column chromatography (Pentane) as a colorless oil. 47 mg (86% over 3 steps). R_f = 0.48 (Pentane). **^{1}H NMR** (600 MHz, $CDCl_3$) 7.54–7.51 (m, 2H), 7.20–7.18 (m, 2H), 7.16 (s, 1H), 7.14–7.10 (m, 2H), 2.29 (s, 3H), 2.18 (s, 2H), 0.07 (s, 9H). **^{13}C NMR** (151 MHz, $CDCl_3$) δ 162.2 (d, *J* = 245.4 Hz), 139.5, 137.6, 137.6, 133.8, 130.6, 128.4 (d, *J* = 8.0 Hz), 127.3, 122.7, 115.5 (d, *J* = 21.2 Hz), 23.9, 20.0, -1.3. **^{19}F NMR** (564 MHz, $CDCl_3$) δ -116.60 (m, 1F). **MS** (70 eV, EI): *m/z* (%): 274 (1), 273 (5), 272 (21) $[M^+]$, 258 (1), 257 (5), 200 (2), 199 (14), 198 (3), 197 (5), 196 (9), 195 (2), 194 (1), 185 (2), 184 (13), 183 (26), 182 (1), 181 (9), 180 (55), 179 (8), 178 (7), 177 (3), 176 (3), 175 (1), 169 (4), 165 (10), 74 (8), 73 (100) $[TMS]^+$. The data are in agreement with those previously reported in the literature.[5]

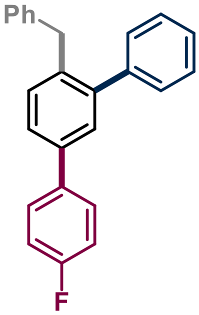

4'-benzyl-4-fluoro-1,1':3',1''-terphenyl (28): General procedure E. The title product was obtained after purification by column chromatography (Pentane) as a colorless oil. 64 mg (89% over 3 steps). R_f = 0.2 (Pentane). **^{1}H NMR** (600 MHz, $CDCl_3$) 7.34–7.30 (m, 3H), 7.29–7.26 (m, 3H), 7.24–7.18 (m, 5H), 7.12–7.09 (m, 2H), 7.08–7.05 (m, 2H), 6.90–6.86 (m, 2H), 4.06 (s, 2H). **^{13}C NMR** (151 MHz, $CDCl_3$) δ 162.8, 140.6, 137.3, 137.2, 131.3 (d, *J* = 7.9 Hz), 131.1, 130.6, 129.8, 129.0, 128.5, 128.0, 127.9, 126.5, 126.2, 114.7 (d, *J* = 21.3 Hz), 41.6. **^{19}F NMR** (564 MHz, $CDCl_3$) δ -116.58k (m, 1F). **MS** (70 eV, E I): *m/z* (%): 340 (3), 339 (26), 338 (100) $[M^+]$, 337 (5), 262 (2), 261 (11), 260 (17), 259 (47), 258 (5), 257 (15), 247 (6), 246 (8), 244 (8), 241 (5), 239 (13), 166 (3), 165 (16), 162 (4). The data are in agreement with those previously reported in the literature.[5]

Computational Studies

DFT calculations were performed using the Gaussian 16 software, Revision A.03.[10] Structural optimizations were performed in the gas phase at the ωB97XD/6-31G(d) level of theory using SDD as an ECP on Pd. Frequency calculations were performed at the same level of theory to confirm whether a structure is a minimum or a transition state and IRC calculations were performed to confirm whether the located transition states connect the correct minima. Single point energies were calculated at the M06/def2-TZVP level of theory, with solvent effects included using the SMD solvation model. In the case of the anionic "ate" complexes that are believed to form in polar solvents, NMP, which was used in the corresponding experiments, was not available in Gaussian's solvent database. Therefore, solvent effects of DMF, which exhibits a similar dielectric constant and similar structure as NMP, were included instead. Images were generated using the CYLview software.[11]

Cartesian coordinates and energies of computed structures

PhOFs

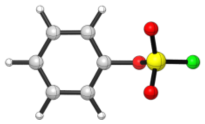

C	-0.669648000000	0.569324000000	0.000000000000
C	-0.668313000000	1.232502000000	1.217214000000
C	-0.668313000000	1.232502000000	-1.217214000000
C	-0.668313000000	2.624035000000	1.208071000000
C	-0.668313000000	2.624035000000	-1.208071000000
C	-0.668916000000	3.317529000000	0.000000000000
H	-0.664052000000	0.665359000000	2.141157000000
H	-0.664052000000	0.665359000000	-2.141157000000
H	-0.667213000000	3.165285000000	2.148596000000
H	-0.667213000000	3.165285000000	-2.148596000000
H	-0.669287000000	4.402859000000	0.000000000000
O	-0.729869000000	-0.841885000000	0.000000000000
S	0.693090000000	-1.581493000000	0.000000000000
O	1.358436000000	-1.402621000000	-1.256404000000
O	1.358436000000	-1.402621000000	1.256404000000
F	0.046361000000	-3.020311000000	0.000000000000

Zero-point correction = 0.109267 (Hartree/Particle)
Thermal correction to Energy = 0.118410
Thermal correction to Enthalpy = 0.119354
Thermal correction to Gibbs Free Energy = 0.072527
Sum of electronic and zero-point Energies = -954.970933
Sum of electronic and thermal Energies = -954.961790
Sum of electronic and thermal Enthalpies = -954.960846
Sum of electronic and thermal Free Energies = -955.007672
E(RM06) (Toluene) = -955.257687213
E(RM06) (DMF) = -955.25955166

PhOTf

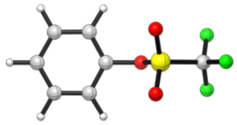

C	-0.403515000000	1.461185000000	0.000000000000
C	-0.300488000000	2.118320000000	1.216795000000
C	-0.300488000000	2.118320000000	-1.216795000000
C	-0.091331000000	3.493995000000	1.207828000000
C	-0.091331000000	3.493995000000	-1.207828000000
C	0.011982000000	4.180029000000	0.000000000000
H	-0.380230000000	1.556694000000	2.140613000000
H	-0.380230000000	1.556694000000	-2.140613000000
H	-0.007898000000	4.028777000000	2.148417000000
H	-0.007898000000	4.028777000000	-2.148417000000
H	0.174830000000	5.253120000000	0.000000000000
O	-0.671374000000	0.077872000000	0.000000000000
S	0.626062000000	-0.893385000000	0.000000000000
O	1.323895000000	-0.799531000000	-1.260234000000
O	1.323895000000	-0.799531000000	1.260234000000
C	-0.371316000000	-2.457098000000	0.000000000000
F	-1.128140000000	-2.510657000000	-1.083132000000
F	0.484283000000	-3.469005000000	0.000000000000
F	-1.128140000000	-2.510657000000	1.083132000000

Zero-point correction = 0.121470 (Hartree/Particle)
Thermal correction to Energy = 0.133700
Thermal correction to Enthalpy = 0.134644
Thermal correction to Gibbs Free Energy = 0.079438
Sum of electronic and zero-point Energies = -1192.675230
Sum of electronic and thermal Energies = -1192.663000
Sum of electronic and thermal Enthalpies = -1192.662056
Sum of electronic and thermal Free Energies = -1192.717262
E(RM06) (Toluene) = -1193.04870932

PhONf

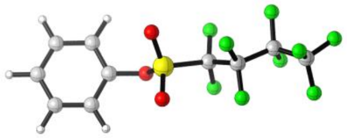

C	-3.604025000000	0.113758000000	-0.392102000000
C	-4.283367000000	-1.075427000000	-0.611935000000
C	-4.229843000000	1.256165000000	0.082784000000
C	-5.649298000000	-1.113213000000	-0.347966000000
C	-5.596242000000	1.201947000000	0.339151000000
C	-6.304197000000	0.021515000000	0.124535000000
H	-3.746890000000	-1.942699000000	-0.980196000000

H	-3.651375000000	2.158080000000	0.247404000000
H	-6.200805000000	-2.033064000000	-0.513561000000
H	-6.106570000000	2.084699000000	0.710663000000
H	-7.369874000000	-0.014207000000	0.327258000000
O	-2.232692000000	0.178879000000	-0.707560000000
S	-1.216785000000	-0.303266000000	0.461669000000
O	-1.417774000000	0.494725000000	1.646985000000
O	-1.161214000000	-1.745369000000	0.513789000000
C	0.303404000000	0.307419000000	-0.437932000000
F	0.304381000000	1.641924000000	-0.364250000000
F	0.225409000000	-0.080050000000	-1.710668000000
C	1.599770000000	-0.267343000000	0.196423000000
C	2.873053000000	0.524103000000	-0.209233000000
C	4.201975000000	-0.221589000000	0.080958000000
F	1.480899000000	-0.226572000000	1.534455000000
F	1.730157000000	-1.541969000000	-0.201310000000
F	2.891204000000	1.681556000000	0.469051000000
F	2.824439000000	0.781832000000	-1.530001000000
F	4.212279000000	-0.673832000000	1.333786000000
F	4.357298000000	-1.242870000000	-0.755205000000
F	5.215061000000	0.625486000000	-0.081643000000

Zero-point correction = 0.159044 (Hartree/Particle)
Thermal correction to Energy = 0.179797
Thermal correction to Enthalpy = 0.180742
Thermal correction to Gibbs Free Energy = 0.105499
Sum of electronic and zero-point Energies = -1905.797526
Sum of electronic and thermal Energies = -1905.776773
Sum of electronic and thermal Enthalpies = -1905.775829
Sum of electronic and thermal Free Energies = -1905.851071
E(RM06) (Toluene) = -1906.45069185

PhOTs

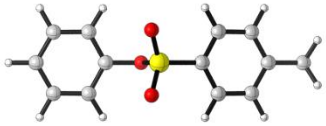

C	-2.241793000000	-0.001029000000	-0.521643000000
C	-2.914428000000	-1.215551000000	-0.540096000000
C	-2.915050000000	1.213136000000	-0.541257000000
C	-4.304913000000	-1.208520000000	-0.588212000000
C	-4.305537000000	1.205299000000	-0.589309000000
C	-5.000080000000	-0.001801000000	-0.614265000000
H	-2.348486000000	-2.139691000000	-0.510844000000
H	-2.349627000000	2.137617000000	-0.512880000000
H	-4.845403000000	-2.149814000000	-0.603051000000
H	-4.846522000000	2.146294000000	-0.605015000000
H	-6.085021000000	-0.002098000000	-0.651952000000
O	-0.842407000000	-0.000862000000	-0.533540000000
S	-0.116892000000	0.002329000000	0.942579000000
O	-0.414406000000	1.262646000000	1.594204000000
O	-0.414650000000	-1.255064000000	1.599689000000
C	1.553917000000	0.000926000000	0.366292000000
C	2.198116000000	1.213988000000	0.146698000000
C	2.198016000000	-1.213222000000	0.152498000000
C	3.510785000000	1.202138000000	-0.306191000000
C	3.510688000000	-1.203650000000	-0.300434000000
C	4.182651000000	-0.001350000000	-0.540209000000
H	1.680325000000	2.146913000000	0.341491000000
H	1.680144000000	-2.145158000000	0.351759000000
H	4.024922000000	2.144201000000	-0.475396000000
H	4.024748000000	-2.146554000000	-0.465126000000
C	5.596762000000	-0.002650000000	-1.060566000000
H	6.144755000000	-0.888181000000	-0.725829000000
H	5.602526000000	-0.005303000000	-2.156965000000
H	6.144810000000	0.884456000000	-0.730117000000

Zero-point correction = 0.226532 (Hartree/Particle)
Thermal correction to Energy = 0.241565
Thermal correction to Enthalpy = 0.242509
Thermal correction to Gibbs Free Energy = 0.180488
Sum of electronic and zero-point Energies = -1125.925345
Sum of electronic and thermal Energies = -1125.910312
Sum of electronic and thermal Enthalpies = -1125.909368
Sum of electronic and thermal Free Energies = -1125.971390
E(RM06) (Toluene) = -1126.24894541

PhOMs

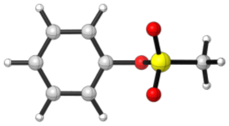

C	-0.162561000000	0.886001000000	0.000000000000
C	0.259953000000	1.408192000000	1.214831000000
C	0.259953000000	1.408192000000	-1.214831000000
C	1.126298000000	2.496881000000	1.207068000000
C	1.126298000000	2.496881000000	-1.207068000000
C	1.557819000000	3.041678000000	0.000000000000
H	-0.084882000000	0.958990000000	2.139245000000
H	-0.084882000000	0.958990000000	-2.139245000000
H	1.466387000000	2.917623000000	2.148090000000
H	1.466387000000	2.917623000000	-2.148090000000
H	2.233621000000	3.891191000000	0.000000000000
O	-1.079367000000	-0.173092000000	0.000000000000
S	-0.427897000000	-1.680095000000	0.000000000000
O	0.259953000000	-1.896323000000	-1.256430000000
O	0.259953000000	-1.896323000000	1.256430000000
C	-1.979132000000	-2.548412000000	0.000000000000
H	-1.741223000000	-3.613547000000	0.000000000000
H	-2.532568000000	-2.279962000000	0.899838000000
H	-2.532568000000	-2.279962000000	-0.899838000000

Zero-point correction = 0.145065 (Hartree/Particle)
Thermal correction to Energy = 0.155121
Thermal correction to Enthalpy = 0.156065
Thermal correction to Gibbs Free Energy = 0.107436
Sum of electronic and zero-point Energies = -895.032722
Sum of electronic and thermal Energies = -895.022665
Sum of electronic and thermal Enthalpies = -895.021721
Sum of electronic and thermal Free Energies = -895.070351
E(RM06) (Toluene) = -895.297031554

PhCl

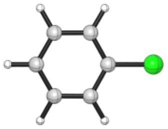

C	0.000000000000	0.000000000000	0.504250000000
C	0.000000000000	1.212597000000	-0.176517000000
C	0.000000000000	-1.212597000000	-0.176517000000
C	0.000000000000	1.204326000000	-1.568422000000
C	0.000000000000	-1.204326000000	-1.568422000000
C	0.000000000000	0.000000000000	-2.266570000000
H	0.000000000000	2.145807000000	0.375952000000
H	0.000000000000	-2.145807000000	0.375952000000
H	0.000000000000	2.147417000000	-2.106361000000
H	0.000000000000	-2.147417000000	-2.106361000000
H	0.000000000000	0.000000000000	-3.351965000000
Cl	0.000000000000	0.000000000000	2.254469000000

Zero-point correction = 0.092346 (Hartree/Particle)
Thermal correction to Energy = 0.097771
Thermal correction to Enthalpy = 0.098716
Thermal correction to Gibbs Free Energy = 0.063271
Sum of electronic and zero-point Energies = -691.653910
Sum of electronic and thermal Energies = -691.648484
Sum of electronic and thermal Enthalpies = -691.647540
Sum of electronic and thermal Free Energies = -691.682984
E(RM06) (DMF) = -691.743781231

TS_OA_Neutral_5-membered_PhOFs

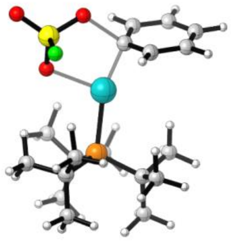

C	2.795479000000	3.680252000000	-0.140279000000
C	2.326572000000	1.544822000000	-1.183433000000
C	2.416874000000	0.969686000000	0.092734000000
C	2.797391000000	1.680736000000	1.238209000000
H	2.940451000000	1.170074000000	2.183777000000
Pd	0.641780000000	0.214311000000	0.142105000000
H	2.217020000000	0.928065000000	-2.070395000000
C	2.947702000000	3.055021000000	1.105151000000
C	2.496916000000	2.935138000000	-1.273060000000
H	3.192863000000	3.647734000000	1.981587000000
H	2.428813000000	3.416119000000	-2.244422000000
O	3.531510000000	-0.690287000000	0.071091000000
S	2.806014000000	-1.996044000000	0.094530000000
O	3.587771000000	-3.123895000000	0.527329000000
O	1.439773000000	-1.879188000000	0.644790000000
H	-0.702534000000	2.727635000000	-0.366758000000
C	-1.787662000000	2.787989000000	-0.224382000000
C	-2.499868000000	1.543269000000	-0.798386000000
H	-2.148206000000	3.674198000000	-0.762273000000
H	-1.980153000000	2.952644000000	0.835013000000
C	-4.018460000000	1.711292000000	-0.634596000000
P	-1.748392000000	-0.019936000000	0.012668000000
C	-2.163109000000	1.546312000000	-2.301152000000
H	-4.578316000000	0.851635000000	-1.010722000000
H	-4.307467000000	1.876046000000	0.406376000000
H	-4.345007000000	2.592132000000	-1.202162000000
C	-2.184974000000	-1.550878000000	-1.047235000000
C	-2.490171000000	-0.234061000000	1.761025000000
H	-2.439311000000	2.523253000000	-2.716591000000
H	-1.089803000000	1.407786000000	-2.472306000000
H	-2.712339000000	0.790741000000	-2.866202000000
C	-1.208332000000	-1.602097000000	-2.243108000000
C	-1.896315000000	-2.833103000000	-0.242688000000
C	-3.630679000000	-1.609475000000	-1.564035000000
C	-2.441333000000	1.111956000000	2.507682000000
C	-3.929841000000	-0.768625000000	1.812274000000
C	-1.563599000000	-1.175198000000	2.561141000000
H	-1.291085000000	-0.749778000000	-2.916911000000
H	-0.170232000000	-1.672381000000	-1.902149000000
H	-1.424461000000	-2.504149000000	-2.829540000000
H	-2.613031000000	-3.002667000000	0.563714000000
H	-1.975420000000	-3.688138000000	-0.925301000000
H	-0.881958000000	-2.835164000000	0.168030000000
H	-3.783446000000	-2.563224000000	-2.084871000000
H	-4.366098000000	-1.555063000000	-0.757136000000
H	-3.852120000000	-0.814512000000	-2.280669000000
H	-1.442146000000	1.559605000000	2.468856000000
H	-3.169126000000	1.835911000000	2.134967000000
H	-2.679926000000	0.930594000000	3.562828000000
H	-4.007335000000	-1.792115000000	1.438184000000
H	-4.268890000000	-0.780990000000	2.856062000000
H	-4.628381000000	-0.146178000000	1.247212000000
H	-1.505224000000	-2.184570000000	2.157761000000
H	-0.543231000000	-0.780251000000	2.609705000000
H	-1.945356000000	-1.250872000000	3.587346000000
H	2.940236000000	4.752650000000	-0.222154000000
F	2.518558000000	-2.238953000000	-1.479008000000

Zero-point correction = 0.484280 (Hartree/Particle)
Thermal correction to Energy = 0.514206
Thermal correction to Enthalpy = 0.515150
Thermal correction to Gibbs Free Energy = 0.423136
Sum of electronic and zero-point Energies = -1897.277404
Sum of electronic and thermal Energies = -1897.247478
Sum of electronic and thermal Enthalpies = -1897.246534
Sum of electronic and thermal Free Energies = -1897.338548
E(RM06) (Toluene) = -1897.90243638

TS_OA_Neutral_5-membered_PhOTf

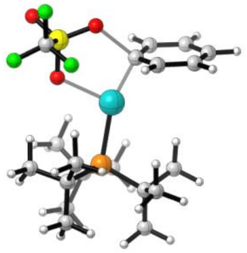

C	1.297027000000	4.469269000000	-0.477984000000
C	1.609657000000	2.180569000000	-1.207704000000
C	1.680356000000	1.818533000000	0.145927000000
C	1.675636000000	2.749739000000	1.193466000000
H	1.830400000000	2.429801000000	2.217804000000
Pd	0.201762000000	0.580164000000	0.188829000000
H	1.795368000000	1.452275000000	-1.990620000000
C	1.445410000000	4.078891000000	0.858888000000
C	1.385874000000	3.532999000000	-1.500905000000
H	1.389090000000	4.821561000000	1.649369000000
H	1.316223000000	3.846290000000	-2.538505000000
O	3.202704000000	0.584555000000	0.458945000000
S	2.933336000000	-0.846858000000	0.835652000000
O	3.788086000000	-1.382776000000	1.875275000000
O	1.476008000000	-1.118407000000	0.973621000000
H	-1.735469000000	2.561564000000	-0.599206000000
C	-2.801272000000	2.331730000000	-0.484706000000
C	-3.122616000000	0.900029000000	-0.967870000000
H	-3.366018000000	3.042468000000	-1.101658000000
H	-3.075301000000	2.515544000000	0.553380000000
C	-4.636273000000	0.658233000000	-0.859748000000
P	-2.015762000000	-0.330599000000	-0.004705000000
C	-2.728000000000	0.881843000000	-2.456455000000
H	-4.923213000000	-0.347761000000	-1.175608000000
H	-5.010688000000	0.815563000000	0.154759000000
H	-5.160525000000	1.370431000000	-1.509846000000
C	-1.964037000000	-1.997008000000	-0.941773000000
C	-2.762337000000	-0.611933000000	1.733083000000
H	-3.236015000000	1.713627000000	-2.959861000000
H	-1.650107000000	1.026779000000	-2.589012000000
H	-3.028500000000	-0.034159000000	-2.968983000000
C	-0.958159000000	-1.863299000000	-2.105714000000
C	-1.368735000000	-3.081494000000	-0.022850000000
C	-3.310670000000	-2.496869000000	-1.486836000000
C	-3.121270000000	0.747391000000	2.361844000000
C	-4.002370000000	-1.517746000000	1.785494000000

C	-1.660239000000	-1.199951000000	2.640989000000
H	-1.253071000000	-1.136380000000	-2.861834000000
H	0.035716000000	-1.590704000000	-1.737577000000
H	-0.872135000000	-2.836822000000	-2.605019000000
H	-2.049737000000	-3.386774000000	0.774387000000
H	-1.170215000000	-3.972695000000	-0.631009000000
H	-0.417581000000	-2.765221000000	0.416803000000
H	-3.168457000000	-3.491022000000	-1.929393000000
H	-4.069736000000	-2.590965000000	-0.706065000000
H	-3.709259000000	-1.850573000000	-2.273032000000
H	-2.280235000000	1.448076000000	2.316862000000
H	-3.996443000000	1.214288000000	1.904951000000
H	-3.358811000000	0.586276000000	3.420479000000
H	-3.775419000000	-2.548453000000	1.502917000000
H	-4.382448000000	-1.542938000000	2.814818000000
H	-4.812035000000	-1.159276000000	1.144697000000
H	-1.313263000000	-2.184534000000	2.332567000000
H	-0.785504000000	-0.543036000000	2.683553000000
H	-2.061790000000	-1.295271000000	3.657976000000
H	1.138655000000	5.516106000000	-0.716378000000
C	3.362140000000	-1.743417000000	-0.719761000000
F	2.660991000000	-1.230138000000	-1.741021000000
F	4.654149000000	-1.623171000000	-0.991032000000
F	3.054129000000	-3.030572000000	-0.604027000000

Zero-point correction = 0.496533 (Hartree/Particle)
Thermal correction to Energy = 0.529474
Thermal correction to Enthalpy = 0.530418
Thermal correction to Gibbs Free Energy = 0.431237
Sum of electronic and zero-point Energies = -2134.982358
Sum of electronic and thermal Energies = -2134.949416
Sum of electronic and thermal Enthalpies = -2134.948472
Sum of electronic and thermal Free Energies = -2135.047653
E(RM06) (Toluene) = -2135.69492830

TS_OA_Neutral_5-membered_PhONf

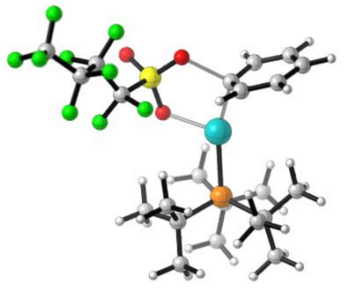

C	1.532272000000	4.612749000000	1.339048000000
C	0.340088000000	2.504052000000	1.330277000000
C	0.475310000000	2.499968000000	-0.066159000000
C	1.021660000000	3.566741000000	-0.792639000000
H	1.022606000000	3.553786000000	-1.876674000000
Pd	1.419324000000	0.819333000000	-0.187132000000
H	-0.259987000000	1.755427000000	1.837409000000
C	1.582250000000	4.605777000000	-0.062387000000
C	0.911568000000	3.580233000000	2.027945000000
H	2.060951000000	5.425317000000	-0.590398000000
H	0.829156000000	3.613791000000	3.110384000000
O	-1.234653000000	2.017485000000	-0.950846000000
S	-1.332851000000	0.713049000000	-1.691955000000
O	-2.101922000000	0.755276000000	-2.919154000000
O	-0.021544000000	0.015180000000	-1.757442000000
H	3.607308000000	1.655138000000	1.509921000000
C	4.521764000000	1.050851000000	1.522800000000
C	4.196608000000	-0.457089000000	1.602860000000
H	5.097175000000	1.330936000000	2.414532000000
H	5.118674000000	1.324487000000	0.653694000000
C	5.507989000000	-1.252382000000	1.698832000000
P	3.056070000000	-0.913154000000	0.133988000000
C	3.420457000000	-0.630497000000	2.921586000000
H	5.341479000000	-2.331541000000	1.741092000000
H	6.181649000000	-1.045077000000	0.863852000000
H	6.035798000000	-0.963616000000	2.616834000000
C	2.194403000000	-2.580734000000	0.501834000000
C	4.118297000000	-1.093681000000	-1.446284000000
H	4.012794000000	-0.190088000000	3.733065000000
H	2.458797000000	-0.106188000000	2.894518000000
H	3.247589000000	-1.676484000000	3.182768000000
C	1.014425000000	-2.315324000000	1.461447000000
C	1.545608000000	-3.114594000000	-0.790122000000
C	3.092270000000	-3.677215000000	1.095046000000
C	5.074595000000	0.108071000000	-1.562673000000
C	4.943891000000	-2.385547000000	-1.548621000000
C	3.182927000000	-0.985737000000	-2.670221000000
H	1.317207000000	-1.950757000000	2.442459000000
H	0.310087000000	-1.597479000000	1.031821000000
H	0.473427000000	-3.257498000000	1.616253000000
H	2.275852000000	-3.478186000000	-1.516106000000
H	0.907292000000	-3.966388000000	-0.524810000000
H	0.908000000000	-2.362883000000	-1.266117000000
H	2.509706000000	-4.602780000000	1.186486000000
H	3.959164000000	-3.896151000000	0.466689000000
H	3.452195000000	-3.425445000000	2.096022000000
H	4.542596000000	1.059882000000	-1.456780000000
H	5.893726000000	0.077203000000	-0.840956000000
H	5.526286000000	0.093696000000	-2.562125000000
H	4.315057000000	-3.274502000000	-1.638323000000
H	5.566476000000	-2.340031000000	-2.451330000000
H	5.612252000000	-2.525587000000	-0.695221000000
H	2.439882000000	-1.778911000000	-2.730742000000
H	2.644560000000	-0.032635000000	-2.677513000000
H	3.794595000000	-1.035246000000	-3.580207000000
H	1.958535000000	5.447676000000	1.885793000000
C	-2.276086000000	-0.368826000000	-0.507295000000
F	-1.757025000000	-0.178123000000	0.727985000000
F	-2.072927000000	-1.643868000000	-0.861049000000
C	-3.799518000000	-0.094733000000	-0.458142000000
C	-4.483011000000	-0.684417000000	0.806152000000
C	-6.025291000000	-0.792078000000	0.699857000000
F	-4.363622000000	-0.643569000000	-1.546063000000
F	-4.014440000000	1.230115000000	-0.462164000000
F	-4.199765000000	0.102240000000	1.857577000000
F	-4.005406000000	-1.922109000000	1.038989000000
F	-6.371726000000	-1.759765000000	-0.142768000000
F	-6.520542000000	-1.076752000000	1.903653000000
F	-6.545117000000	0.360976000000	0.283165000000

Zero-point correction = 0.534250 (Hartree/Particle)
Thermal correction to Energy = 0.575673
Thermal correction to Enthalpy = 0.576617
Thermal correction to Gibbs Free Energy = 0.458256
Sum of electronic and zero-point Energies = -2848.101483
Sum of electronic and thermal Energies = -2848.060060
Sum of electronic and thermal Enthalpies = -2848.059116
Sum of electronic and thermal Free Energies = -2848.177477
E(RM06) (Toluene) = -2849.09831846

TS_OA_Neutral_5-membered_PhOTs

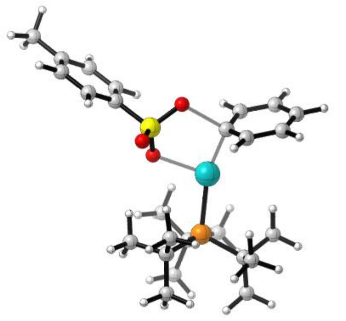

C	-0.735880000000	4.897090000000	0.350342000000
C	-0.221769000000	3.016024000000	-1.085809000000
C	0.405480000000	2.410534000000	0.013466000000
C	0.567025000000	3.057097000000	1.246278000000
H	1.134408000000	2.586773000000	2.041850000000
Pd	-0.571965000000	0.747469000000	0.075182000000
H	-0.178867000000	2.558772000000	-2.069911000000
C	-0.042805000000	4.294960000000	1.407969000000
C	-0.818650000000	4.268111000000	-0.885995000000
H	0.026904000000	4.800914000000	2.366790000000
H	-1.317632000000	4.758076000000	-1.717259000000
O	2.235778000000	1.688025000000	-0.487649000000
S	2.250017000000	0.218938000000	-0.836710000000
O	1.269895000000	-0.517697000000	0.037877000000
H	-3.199048000000	1.929404000000	0.369144000000
C	-4.053600000000	1.285263000000	0.606942000000
C	-4.016727000000	-0.021445000000	-0.215886000000
H	-4.969012000000	1.833933000000	0.350323000000
H	-4.064331000000	1.123798000000	1.684199000000
C	-5.268264000000	-0.853466000000	0.104508000000
P	-2.337564000000	-0.888765000000	0.094683000000
C	-4.089919000000	0.430082000000	-1.686570000000
H	-5.292348000000	-1.800588000000	-0.439923000000
H	-5.359134000000	-1.072311000000	1.171430000000
H	-6.161249000000	-0.283883000000	-0.183814000000
C	-2.025743000000	-2.167293000000	-1.293647000000
C	-2.409098000000	-1.795032000000	1.776657000000
H	-4.968350000000	1.076063000000	-1.807789000000
H	-3.208956000000	1.015959000000	-1.971201000000
H	-4.202697000000	-0.400545000000	-2.385896000000
C	-1.494314000000	-1.413113000000	-2.533166000000
C	-0.876920000000	-3.107569000000	-0.879853000000
C	-3.240185000000	-3.019927000000	-1.692763000000
C	-2.988105000000	-0.849838000000	2.845551000000
C	-3.214305000000	-3.103477000000	1.795628000000
C	-0.959564000000	-2.077584000000	2.227292000000
H	-2.195134000000	-0.684576000000	-2.940150000000
H	-0.550587000000	-0.902664000000	-2.314436000000
H	-1.292445000000	-2.147737000000	-3.323266000000
H	-1.163028000000	-3.813764000000	-0.096936000000
H	-0.592713000000	-3.701129000000	-1.757657000000
H	0.007488000000	-2.546963000000	-0.562242000000
H	-2.921813000000	-3.765063000000	-2.432967000000
H	-3.669304000000	-3.561862000000	-0.845718000000
H	-4.034213000000	-2.428400000000	-2.155758000000
H	-2.475093000000	0.118149000000	2.849115000000
H	-4.061481000000	-0.681766000000	2.733431000000
H	-2.835760000000	-1.307189000000	3.831044000000
H	-2.761862000000	-3.878278000000	1.172417000000
H	-3.242582000000	-3.491004000000	2.822328000000
H	-4.247793000000	-2.964133000000	1.468199000000
H	-0.417753000000	-2.752612000000	1.567216000000
H	-0.379427000000	-1.151757000000	2.295566000000
H	-0.987976000000	-2.537724000000	3.223658000000
H	-1.188835000000	5.873334000000	0.491837000000
O	2.106630000000	-0.061276000000	-2.262139000000
C	3.845186000000	-0.346725000000	-0.296296000000
C	4.171405000000	-0.304284000000	1.058140000000
C	4.750235000000	-0.819077000000	-1.235639000000
C	5.422370000000	-0.742507000000	1.464604000000
C	6.002738000000	-1.255843000000	-0.809847000000
C	6.357407000000	-1.222621000000	0.538255000000
H	3.448947000000	0.060969000000	1.780481000000
H	4.469256000000	-0.846326000000	-2.282867000000
H	5.681860000000	-0.715073000000	2.520014000000
H	6.713425000000	-1.629036000000	-1.542472000000
C	7.718356000000	-1.678800000000	0.999917000000
H	8.267159000000	-2.178053000000	0.196214000000
H	8.321148000000	-0.827880000000	1.338283000000
H	7.637880000000	-2.376725000000	1.840274000000

Zero-point correction = 0.601305 (Hartree/Particle)
Thermal correction to Energy = 0.637253
Thermal correction to Enthalpy = 0.638198
Thermal correction to Gibbs Free Energy = 0.529537
Sum of electronic and zero-point Energies = -2068.220968
Sum of electronic and thermal Energies = -2068.185020
Sum of electronic and thermal Enthalpies = -2068.184076
Sum of electronic and thermal Free Energies = -2068.292736
E(RM06) (Toluene) = -2068.88363126

TS_OA_Neutral_5-membered_PhOMs

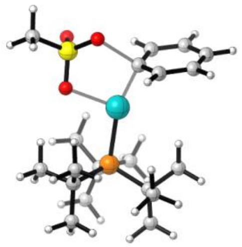

C	2.878611000000	3.694837000000	0.106573000000
C	2.253737000000	1.812831000000	-1.282408000000
C	2.381675000000	0.998850000000	-0.148856000000
C	2.855910000000	1.472309000000	1.082996000000
H	3.035325000000	0.782279000000	1.900663000000
Pd	0.616253000000	0.215573000000	-0.110620000000
H	2.047802000000	1.381555000000	-2.257284000000
C	3.067146000000	2.840278000000	1.200752000000
C	2.484618000000	3.186811000000	-1.124840000000
H	3.393427000000	3.246154000000	2.154090000000
H	2.383335000000	3.845742000000	-1.982573000000
O	3.432540000000	-0.696443000000	-0.619399000000
S	2.804925000000	-1.876756000000	0.080595000000
O	3.154449000000	-1.989035000000	1.493410000000
O	1.323928000000	-1.912072000000	-0.190449000000
H	-0.770223000000	2.746293000000	-0.188619000000
C	-1.824556000000	2.782643000000	0.108792000000
C	-2.621007000000	1.606167000000	-0.497592000000
H	-2.248089000000	3.722054000000	-0.269459000000
H	-1.865176000000	2.824168000000	1.196450000000
C	-4.100314000000	1.746677000000	-0.106548000000
P	-1.774629000000	-0.033185000000	0.017826000000
C	-2.495574000000	1.782228000000	-2.022460000000
H	-4.716191000000	0.931811000000	-0.494729000000
H	-4.239688000000	1.791657000000	0.976471000000
H	-4.493597000000	2.684187000000	-0.520532000000
C	-2.374263000000	-1.435052000000	-1.137295000000
C	-2.271031000000	-0.450984000000	1.816697000000
H	-2.816412000000	2.798556000000	-2.282562000000

H	-1.457742000000	1.669675000000	-2.355158000000
H	-3.125298000000	1.092564000000	-2.588027000000
C	-1.567420000000	-1.345353000000	-2.451362000000
C	-1.994260000000	-2.798991000000	-0.529020000000
C	-3.877126000000	-1.441481000000	-1.456168000000
C	-2.103810000000	0.801471000000	2.697220000000
C	-3.696911000000	-0.991768000000	2.005546000000
C	-1.256311000000	-1.475566000000	2.370180000000
H	-1.742115000000	-0.427811000000	-3.012910000000
H	-0.493900000000	-1.430738000000	-2.253314000000
H	-1.858897000000	-2.183972000000	-3.096946000000
H	-2.599408000000	-3.061139000000	0.341492000000
H	-2.174698000000	-3.571505000000	-1.287257000000
H	-0.934488000000	-2.839486000000	-0.259986000000
H	-4.108987000000	-2.328606000000	-2.059539000000
H	-4.495035000000	-1.487004000000	-0.555466000000
H	-4.186926000000	-0.569207000000	-2.037370000000
H	-1.113907000000	1.254056000000	2.573201000000
H	-2.867283000000	1.560505000000	2.512423000000
H	-2.197916000000	0.499989000000	3.747660000000
H	-3.837522000000	-1.967403000000	1.534330000000
H	-3.889728000000	-1.122133000000	3.078230000000
H	-4.458747000000	-0.312370000000	1.614747000000
H	-1.277403000000	-2.435369000000	1.857053000000
H	-0.230565000000	-1.096929000000	2.313272000000
H	-1.490875000000	-1.660399000000	3.426446000000
H	3.068971000000	4.757853000000	0.216679000000
C	3.451223000000	-3.289104000000	-0.797327000000
H	4.534480000000	-3.304450000000	-0.671480000000
H	3.004886000000	-4.186154000000	-0.365332000000
H	3.186228000000	-3.195322000000	-1.850811000000

Zero-point correction = 0.520345 (Hartree/Particle)
Thermal correction to Energy = 0.551027
Thermal correction to Enthalpy = 0.551971
Thermal correction to Gibbs Free Energy = 0.459115
Sum of electronic and zero-point Energies = -1837.331642
Sum of electronic and thermal Energies = -1837.300960
Sum of electronic and thermal Enthalpies = -1837.300016
Sum of electronic and thermal Free Energies = -1837.392872
E(RM06) (Toluene) = -1837.93215515

TS_OA_Anionic_PhOFs

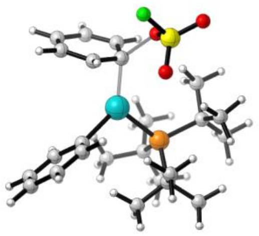

C	-2.324522000000	2.391256000000	2.876956000000
C	-2.194030000000	0.008944000000	2.379008000000
C	-1.970208000000	0.313042000000	1.000826000000
C	-2.036846000000	1.683815000000	0.561416000000
H	-2.271229000000	1.920864000000	-0.473898000000
H	-2.212632000000	-1.031040000000	2.692147000000
C	-2.230244000000	2.700748000000	1.546338000000
C	-2.301624000000	1.027693000000	3.287358000000
H	-2.327540000000	3.730973000000	1.211917000000
H	-2.365421000000	0.789745000000	4.347518000000
O	-3.181379000000	-0.776552000000	0.178126000000
S	-3.432889000000	-0.745906000000	-1.308887000000
O	-2.302428000000	-0.319932000000	-2.108278000000
H	2.206306000000	-4.209563000000	-0.306131000000
C	1.272723000000	-4.166288000000	0.257462000000
C	0.409688000000	-2.935548000000	-0.073059000000
H	1.514413000000	-4.223049000000	1.322747000000
H	0.698639000000	-5.070508000000	0.012153000000
P	1.267802000000	-1.213660000000	0.085019000000
C	-0.833159000000	-2.981899000000	0.836264000000
C	-0.141246000000	-3.085340000000	-1.504461000000
C	2.526429000000	-1.096080000000	-1.370652000000
C	2.280115000000	-1.252970000000	1.721486000000
H	-1.546988000000	-2.204037000000	0.570602000000
H	-1.325661000000	-3.953634000000	0.694189000000
H	-0.602892000000	-2.886915000000	1.897042000000
H	-0.829152000000	-3.940582000000	-1.525051000000
H	-0.709149000000	-2.202055000000	-1.813559000000
H	0.639370000000	-3.284356000000	-2.242940000000
C	1.742661000000	-0.647211000000	-2.621245000000
C	3.544298000000	0.027246000000	-1.095473000000
C	3.315806000000	-2.369180000000	-1.717967000000
C	1.388303000000	-1.746426000000	2.876201000000
C	3.564029000000	-2.098741000000	1.712189000000
C	2.641561000000	0.197911000000	2.092882000000
H	1.024015000000	-1.386586000000	-2.973361000000
H	1.204335000000	0.280993000000	-2.425674000000
H	2.457255000000	-0.462633000000	-3.435404000000
H	4.262784000000	-0.230527000000	-0.312923000000
H	4.119463000000	0.200315000000	-2.014970000000
H	3.048213000000	0.964763000000	-0.835470000000
H	4.034070000000	-2.126678000000	-2.513396000000
H	3.886711000000	-2.758850000000	-0.870858000000
H	2.676735000000	-3.169023000000	-2.099508000000
H	0.430363000000	-1.216258000000	2.895704000000
H	1.202553000000	-2.822653000000	2.842343000000
H	1.904128000000	-1.537624000000	3.823438000000
H	4.322409000000	-1.694882000000	1.037577000000
H	3.996987000000	-2.094930000000	2.722451000000
H	3.383972000000	-3.140388000000	1.436957000000
H	3.217203000000	0.718195000000	1.328618000000
H	1.731480000000	0.779362000000	2.266732000000
H	3.233586000000	0.188047000000	3.019498000000
Pd	-0.167753000000	0.664041000000	0.182920000000
H	-2.451273000000	3.173708000000	3.620034000000
C	1.038221000000	2.298378000000	-0.433072000000
C	0.922143000000	2.784761000000	-1.753458000000
C	1.826273000000	3.100938000000	0.416032000000
C	1.565527000000	3.938369000000	-2.203410000000
C	2.479934000000	4.257099000000	-0.014293000000
C	2.361899000000	4.681829000000	-1.335345000000
H	0.295432000000	2.246228000000	-2.465212000000
H	1.938829000000	2.820624000000	1.462386000000
H	1.440228000000	4.259818000000	-3.236804000000
H	3.082095000000	4.831764000000	0.689036000000
H	2.871512000000	5.579745000000	-1.678810000000
O	-4.199439000000	-1.895131000000	-1.737312000000
F	-4.463969000000	0.499332000000	-1.414306000000

Zero-point correction = 0.574568 (Hartree/Particle)
Thermal correction to Energy = 0.609594
Thermal correction to Enthalpy = 0.610538
Thermal correction to Gibbs Free Energy = 0.508967
Sum of electronic and zero-point Energies = -2128.797624
Sum of electronic and thermal Energies = -2128.762597
Sum of electronic and thermal Enthalpies = -2128.761653
Sum of electronic and thermal Free Energies = -2128.863224
E(RM06) (DMF) = -2129.55283351

TS_OA_Anionic_PhCl

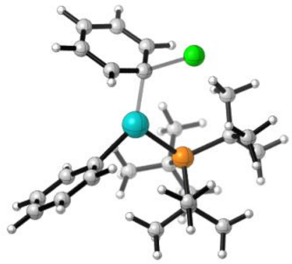

C	-3.374051000000	-3.044251000000	1.433113000000
C	-0.996812000000	-3.155664000000	0.918054000000
C	-1.170658000000	-2.180322000000	-0.085715000000
C	-2.481370000000	-1.727967000000	-0.393985000000
H	-2.684782000000	-1.239878000000	-1.346394000000
Pd	-0.810137000000	-0.221953000000	-0.131385000000
Cl	-0.401009000000	-3.114629000000	-2.302399000000
H	-0.008940000000	-3.556151000000	1.120819000000
C	-3.572176000000	-2.159427000000	0.392619000000
C	-2.080037000000	-3.545881000000	1.681910000000
H	-4.576078000000	-1.830910000000	0.132779000000
H	-1.925504000000	-4.237976000000	2.508142000000
C	4.177699000000	-0.896866000000	0.003770000000
C	2.731361000000	-1.055084000000	-0.501365000000
H	4.261336000000	-1.106821000000	1.074211000000
H	4.803404000000	-1.636246000000	-0.514278000000
H	4.610990000000	0.086301000000	-0.186125000000
P	1.473715000000	0.276033000000	0.104640000000
C	2.248489000000	-2.459704000000	-0.092858000000
C	2.735497000000	-1.078450000000	-2.042278000000
C	1.941555000000	1.916645000000	-0.797549000000
C	1.808327000000	0.538148000000	1.982723000000
H	1.309956000000	-2.706900000000	-0.592484000000
H	3.000492000000	-3.184743000000	-0.434075000000
H	2.139046000000	-2.589481000000	0.983103000000
H	3.296387000000	-1.964110000000	-2.367140000000
H	1.720592000000	-1.189171000000	-2.438801000000
H	3.223257000000	-0.206645000000	-2.485838000000
C	1.286265000000	1.876260000000	-2.193144000000
C	1.316119000000	3.127872000000	-0.080370000000
C	3.442297000000	2.207090000000	-0.970529000000
C	1.840611000000	-0.822074000000	2.704569000000
C	3.092883000000	1.303246000000	2.343380000000
C	0.605273000000	1.283489000000	2.589365000000
H	1.640471000000	1.056512000000	-2.817283000000
H	0.203144000000	1.790495000000	-2.102607000000
H	1.511164000000	2.817681000000	-2.713728000000
H	1.806753000000	3.361429000000	0.868259000000
H	1.435146000000	4.006599000000	-0.728496000000
H	0.247323000000	2.987852000000	0.091153000000
H	3.550870000000	3.195994000000	-1.436905000000
H	3.980729000000	2.235346000000	-0.019492000000
H	3.938164000000	1.488570000000	-1.626745000000
H	0.976333000000	-1.438515000000	2.436327000000
H	1.797343000000	-0.638336000000	3.786833000000
H	3.062177000000	2.344615000000	2.014941000000
H	3.199913000000	1.313850000000	3.437137000000
H	3.992878000000	0.842626000000	1.931206000000
H	0.407820000000	2.249075000000	2.125867000000
H	-0.301717000000	0.680289000000	2.490012000000
H	0.797314000000	1.451067000000	3.658913000000
H	2.755535000000	-1.386229000000	2.509757000000
C	-1.773597000000	1.671111000000	-0.129437000000
C	-2.248637000000	2.356413000000	1.006669000000
C	-2.216580000000	2.203924000000	-1.359570000000
C	-3.055749000000	3.492966000000	0.931568000000
C	-3.025520000000	3.336748000000	-1.456871000000
C	-3.444945000000	3.999210000000	-0.305920000000
H	-1.984871000000	1.994480000000	1.998895000000
H	-1.927183000000	1.712393000000	-2.289295000000
H	-3.385749000000	3.983177000000	1.846888000000
H	-3.331336000000	3.701115000000	-2.436790000000
H	-4.070833000000	4.886663000000	-0.371801000000
H	-4.211295000000	-3.380568000000	2.039033000000

Zero-point correction = 0.558843 (Hartree/Particle)
Thermal correction to Energy = 0.590078
Thermal correction to Enthalpy = 0.591022
Thermal correction to Gibbs Free Energy = 0.498911
Sum of electronic and zero-point Energies = -1865.454696
Sum of electronic and thermal Energies = -1865.423462
Sum of electronic and thermal Enthalpies = -1865.422517
Sum of electronic and thermal Free Energies = -1865.514628
E(RM06) (DMF) = -1866.02623051

References

[1] M. Aufiero, T. Scattolin, F. Proutière, F. Schoenebeck, *Organometallics* **2015**, *34*, 5191-5195.

[2] E. Zhang, J. Tang, S. Li, P. Wu, J. E. Moses, K. B. Sharpless, *Chem. Eur. J.* **2016**, *22*, 5692-5697.

[3] C. Veryser, J. Demaerel, V. Bieliūnas, P. Gilles, W. M. De Borggraeve, *Org. Lett.* **2017**, *19*, 5244-5247.

[4] W. P. Gallagher, A. Vo, *Organic Process Research & Development* **2015**, *19*, 1369-1373.

[5] S. T. Keaveney, G. Kundu, F. Schoenebeck, *Angew. Chem. Int. Ed.* **2018**, *57*, 12573-12577.

[6] F. M. Piller, A. Metzger, M. A. Schade, B. A. Haag, A. Gavryushin, P. Knochel, *Chem. Eur. J.* **2009**, *15*, 7192-7202.

[7] H. Zhou, P. Mukherjee, R. Liu, E. Evrard, D. Wang, J. M. Humphrey, T. W. Butler, L. R. Hoth, J. B. Sperry, S. K. Sakata, C. J. Helal, C. W. am Ende, *Org. Lett.* **2018**, *20*, 812-815.

[8] P. S. Hanley, M. S. Ober, A. L. Krasovskiy, G. T. Whiteker, W. J. Kruper, *ACS Catal.* **2015**, *5*, 5041-5046.

[9] P. Gilles, C. Veryser, S. Vangrunderbeeck, S. Ceusters, L. Van Meervelt, W. M. De Borggraeve, *J. Org. Chem.* **2019**, *84*, 1070-1078.

[10] M. J. Frisch, G. W. Trucks, H. B. Schlegel, G. E. Scuseria, M. A. Robb, J. R. Cheeseman, G. Scalmani, V. Barone, G. A. Petersson, H. Nakatsuji, X. Li, M. Caricato, A. V. Marenich, J. Bloino, B. G. Janesko, R. Gomperts, B. Mennucci, H. P. Hratchian, J. V. Ortiz, A. F. Izmaylov, J. L. Sonnenberg, Williams, F. Ding, F. Lipparini, F. Egidi, J. Goings, B. Peng, A. Petrone, T. Henderson, D. Ranasinghe, V. G. Zakrzewski, J. Gao, N. Rega, G. Zheng, W. Liang, M. Hada, M. Ehara, K. Toyota, R. Fukuda, J. Hasegawa, M. Ishida, T. Nakajima, Y. Honda, O. Kitao, H. Nakai, T. Vreven, K. Throssell, J. A. Montgomery Jr., J. E. Peralta, F. Ogliaro, M. J. Bearpark, J. J. Heyd, E. N. Brothers, K. N. Kudin, V. N. Staroverov, T. A. Keith, R. Kobayashi, J. Normand, K. Raghavachari, A. P. Rendell, J. C. Burant, S. S. Iyengar, J. Tomasi, M. Cossi, J. M. Millam, M. Klene, C. Adamo, R. Cammi, J. W. Ochterski, R. L. Martin, K. Morokuma, O. Farkas, J. B. Foresman, D. J. Fox, *Gaussian 16*, *Revision A.03*, **2016**, Gaussian, Inc., Wallingford, CT.

[11] C. Y. Legault, *CYLview*, Version 1.0b, **2009**, Université de Sherbrooke, (http://www.cylview.org).

NMR spectra

Aryl fluorosulfates

1H NMR
(599.86 MHz, CDCl3)

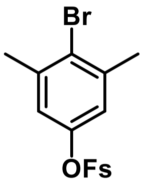

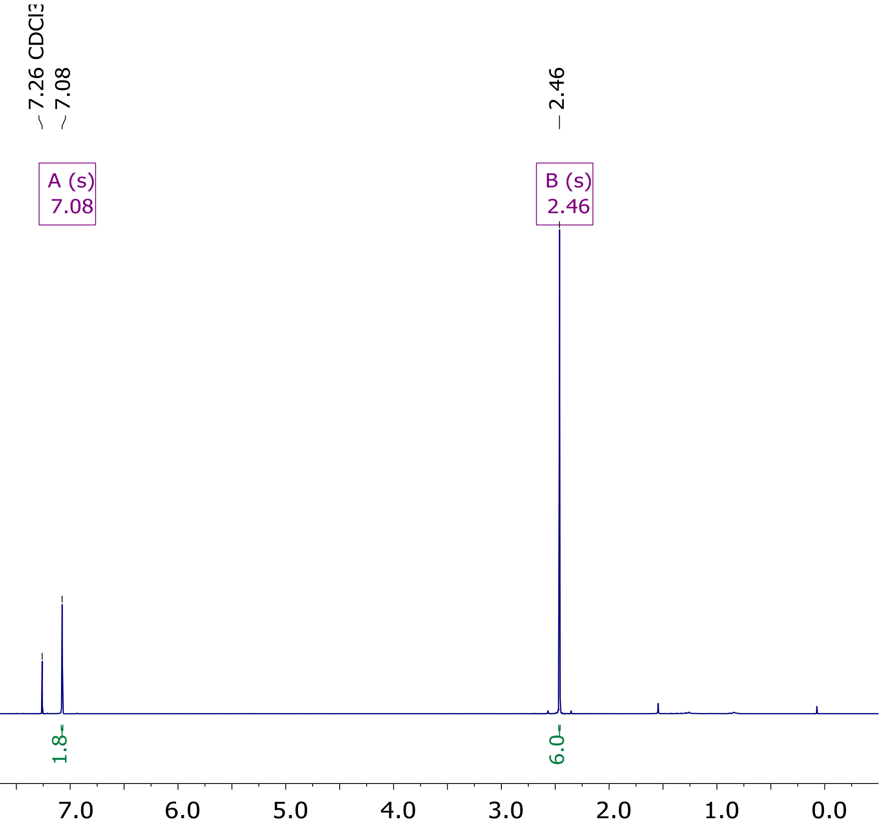

13C NMR
(150.85 MHz, CDCl3)

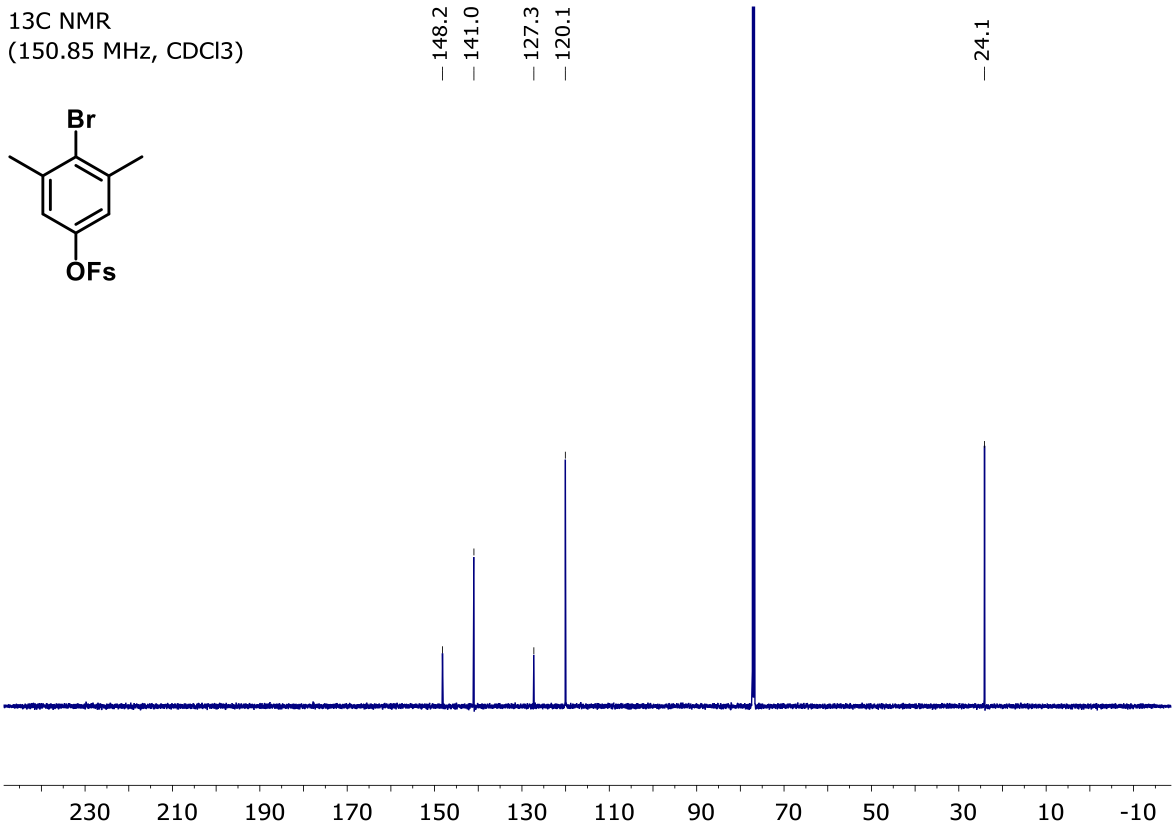

19F NMR
(564.40 MHz, CDCl3)

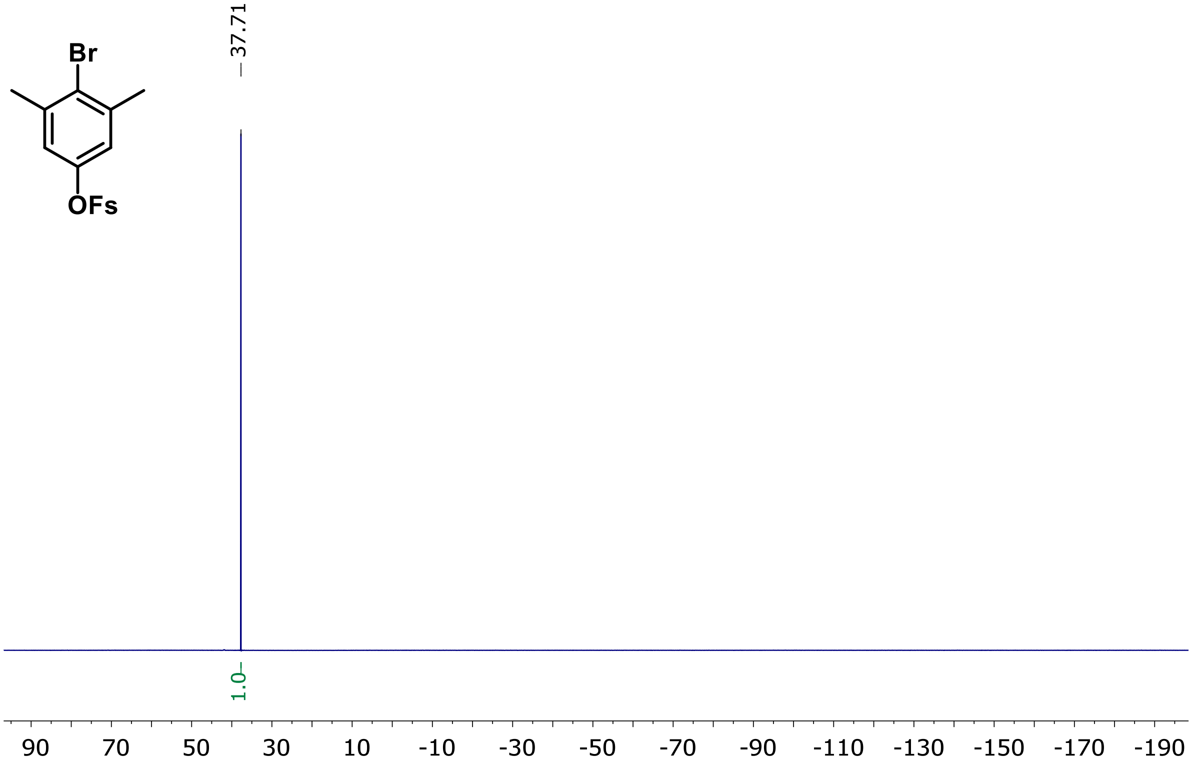

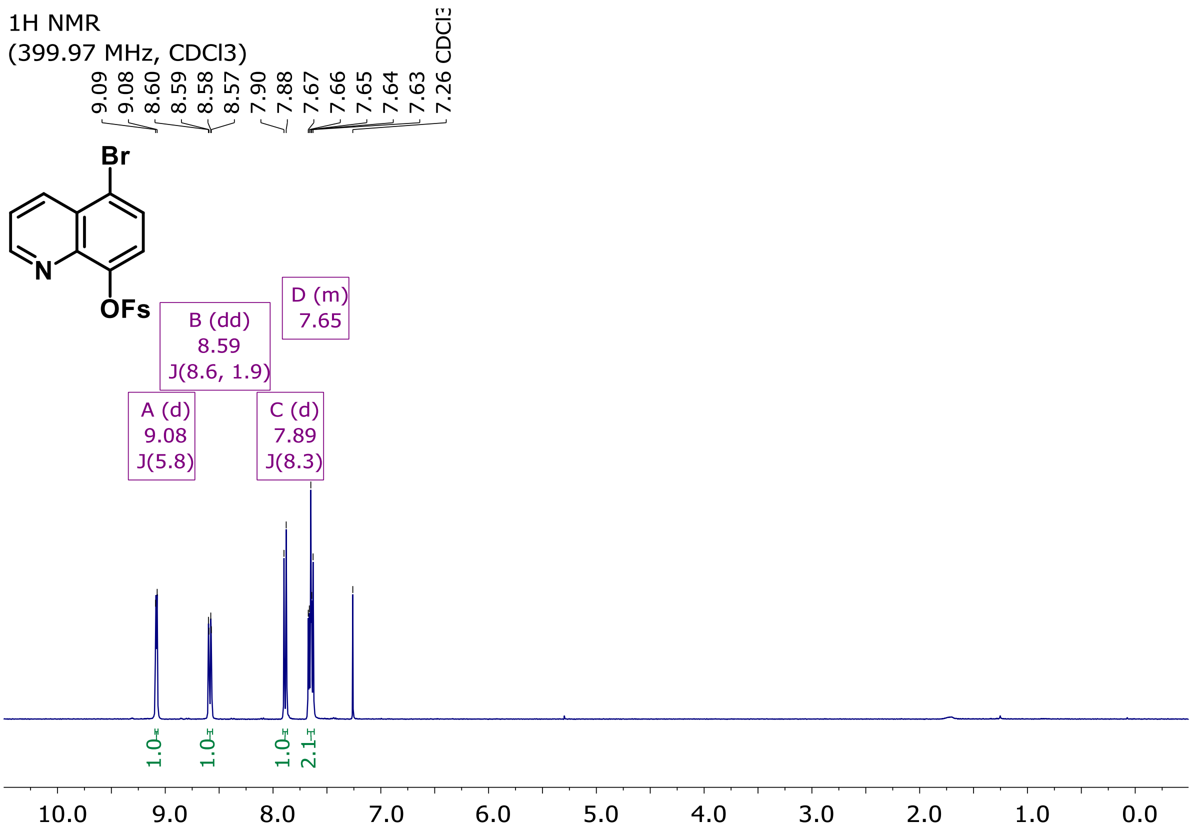
1H NMR
(399.97 MHz, CDCl3)
9.09
9.08
8.60
8.59
8.58
8.57
7.90
7.88
7.67
7.66
7.65
7.64
7.63
7.26 CDCl3
Br
N
OFs
B (dd)
8.59
J(8.6, 1.9)
D (m)
7.65
A (d)
9.08
J(5.8)
C (d)
7.89
J(8.3)
1.0
1.0
1.0
2.1
10.0
9.0
8.0
7.0
6.0
5.0
4.0
3.0
2.0
1.0
0.0

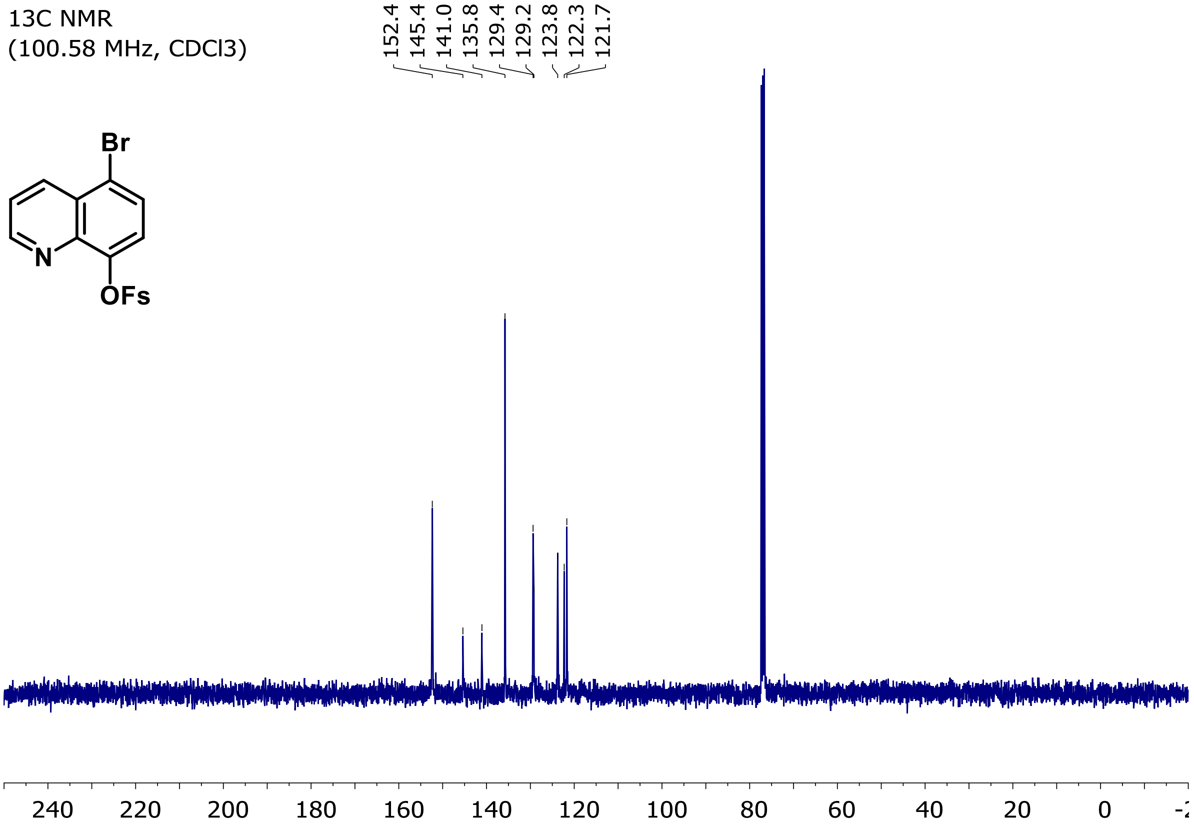
13C NMR
(100.58 MHz, CDCl3)
152.4
145.4
141.0
135.8
129.4
129.2
123.8
122.3
121.7
Br
N
OFs
240
220
200
180
160
140
120
100
80
60
40
20
0

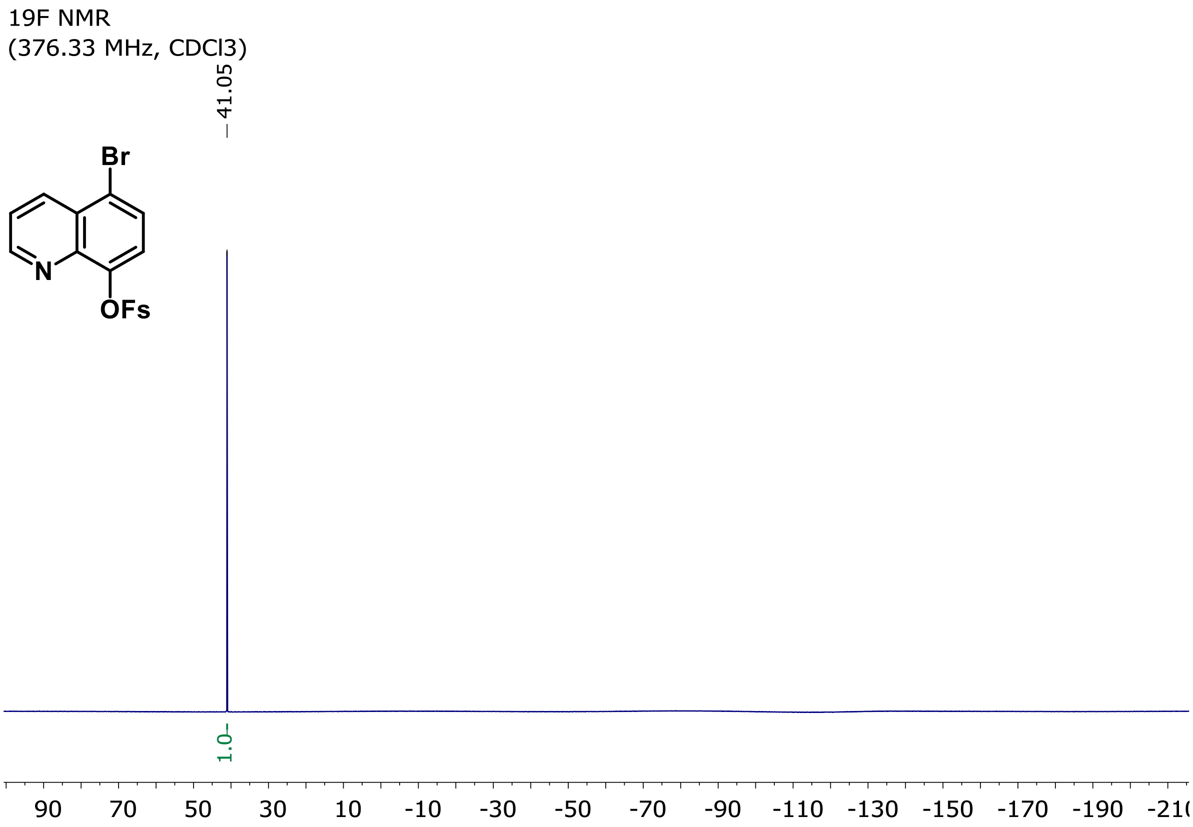
19F NMR
(376.33 MHz, CDCl3)
41.05
Br
N
OFs
1.0
90
70
50
30
10
-10
-30
-50
-70
-90
-110
-130
-150
-170
-190

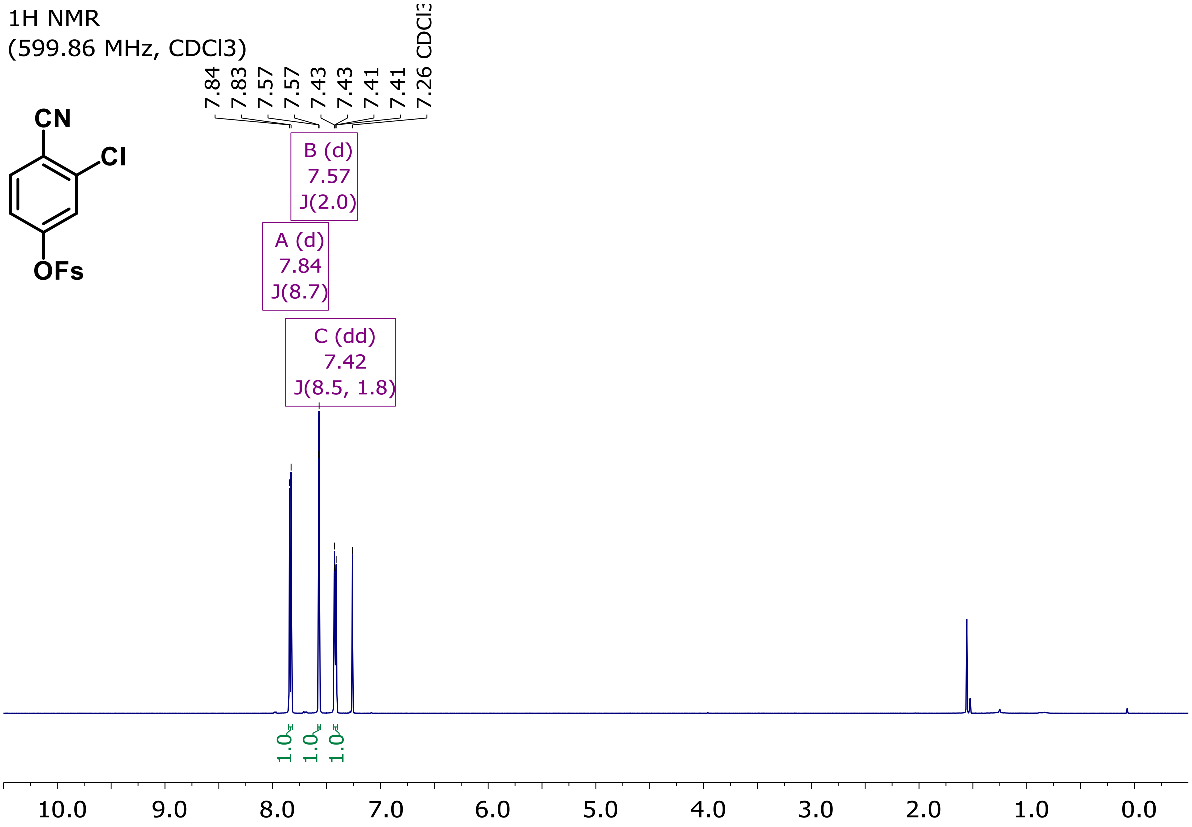
1H NMR
(599.86 MHz, CDCl3)
7.84
7.83
7.57
7.57
7.43
7.43
7.41
7.41
7.26 CDCl3
CN
Cl
OFs
B (d)
7.57
J(2.0)
A (d)
7.84
J(8.7)
C (dd)
7.42
J(8.5, 1.8)
1.0
1.0
1.0
10.0
9.0
8.0
7.0
6.0
5.0
4.0
3.0
2.0
1.0
0.0

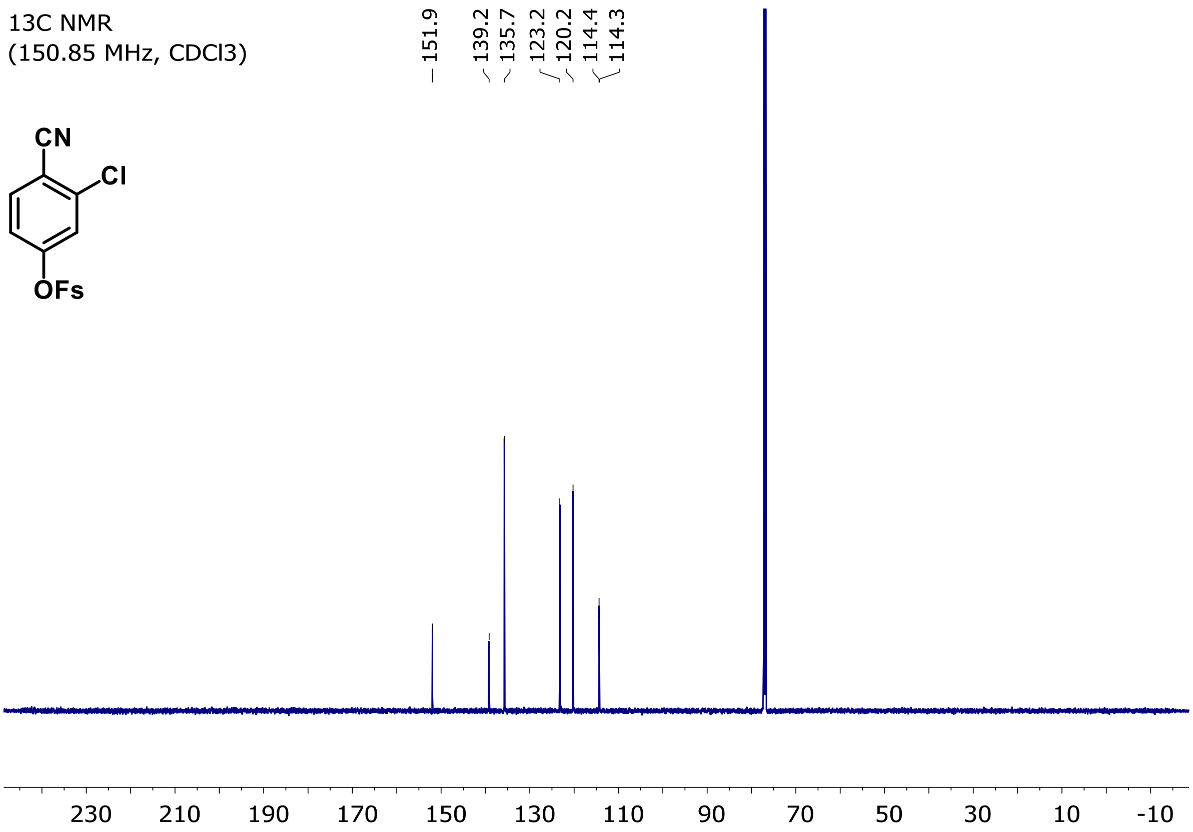
13C NMR
(150.85 MHz, CDCl3)
CN
Cl
OFs
151.9
139.2
135.7
123.2
120.2
114.4
114.3
230
210
190
170
150
130
110
90
70
50
30
10
-10

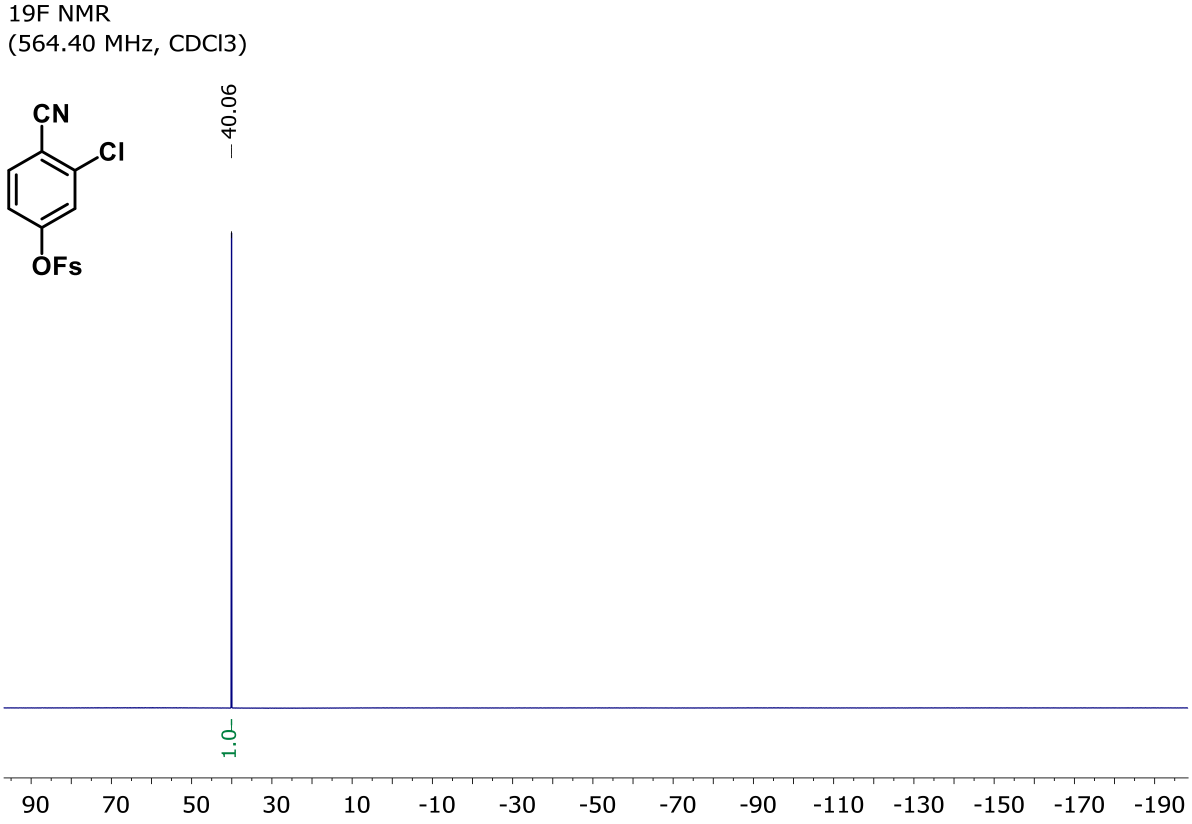
19F NMR
(564.40 MHz, CDCl3)
CN
Cl
OFs
40.06
1.0
90
70
50
30
10
-10
-30
-50
-70
-90
-110
-130
-150
-170
-190

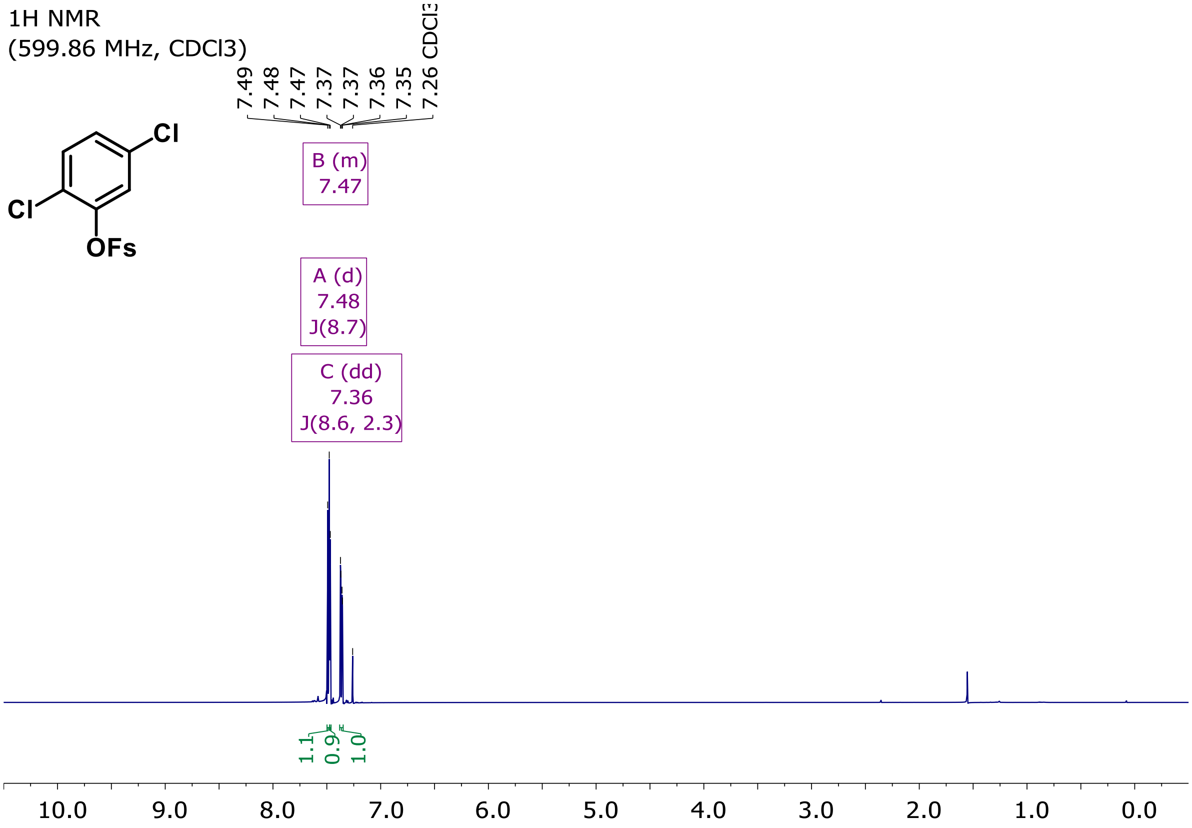
1H NMR
(599.86 MHz, CDCl3)
7.49
7.48
7.47
7.37
7.37
7.36
7.35
7.26 CDCl3
Cl
Cl
OFs
B (m)
7.47
A (d)
7.48
J(8.7)
C (dd)
7.36
J(8.6, 2.3)
1.1
0.9
1.0
10.0
9.0
8.0
7.0
6.0
5.0
4.0
3.0
2.0
1.0
0.0

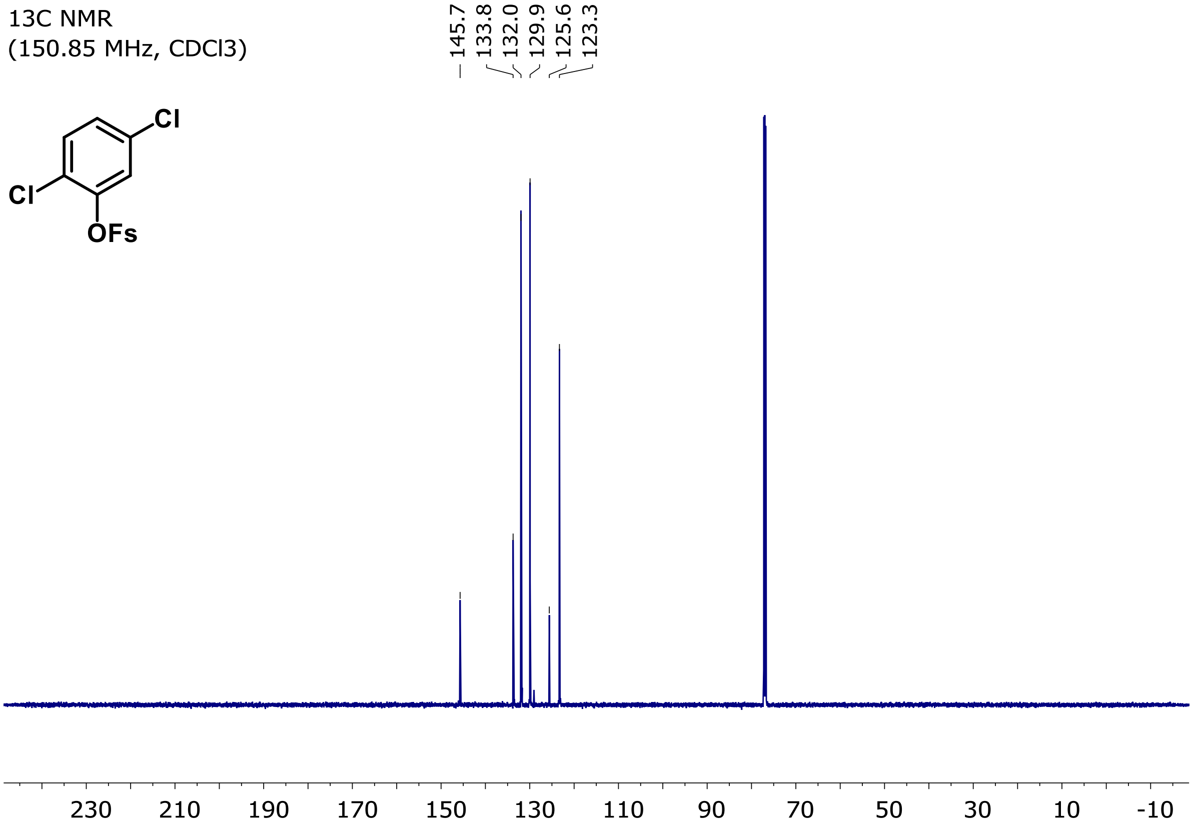
13C NMR
(150.85 MHz, CDCl3)
145.7
133.8
132.0
129.9
125.6
123.3
Cl
Cl
OFs
230
210
190
170
150
130
110
90
70
50
30
10
-10

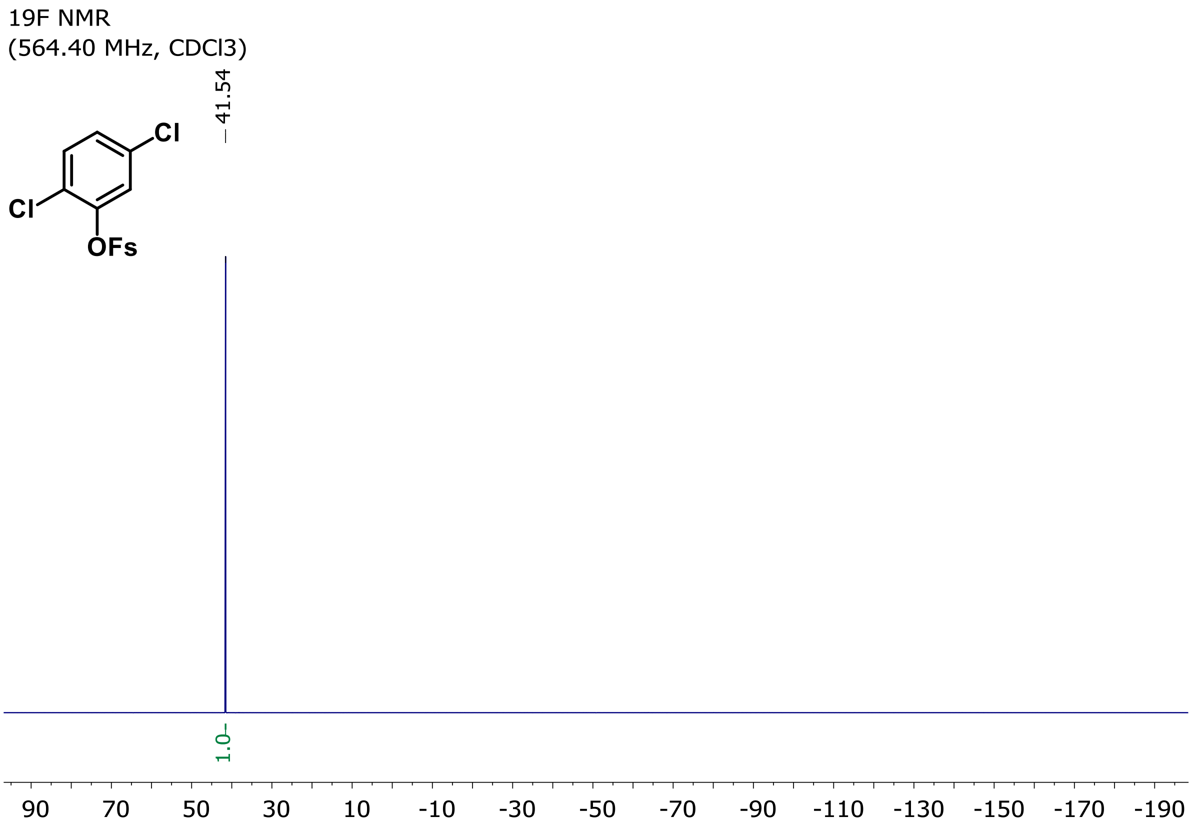

19F NMR
(564.40 MHz, CDCl3)
41.54
Cl
Cl
OFs
1.0
90
70
50
30
10
-10
-30
-50
-70
-90
-110
-130
-150
-170
-190

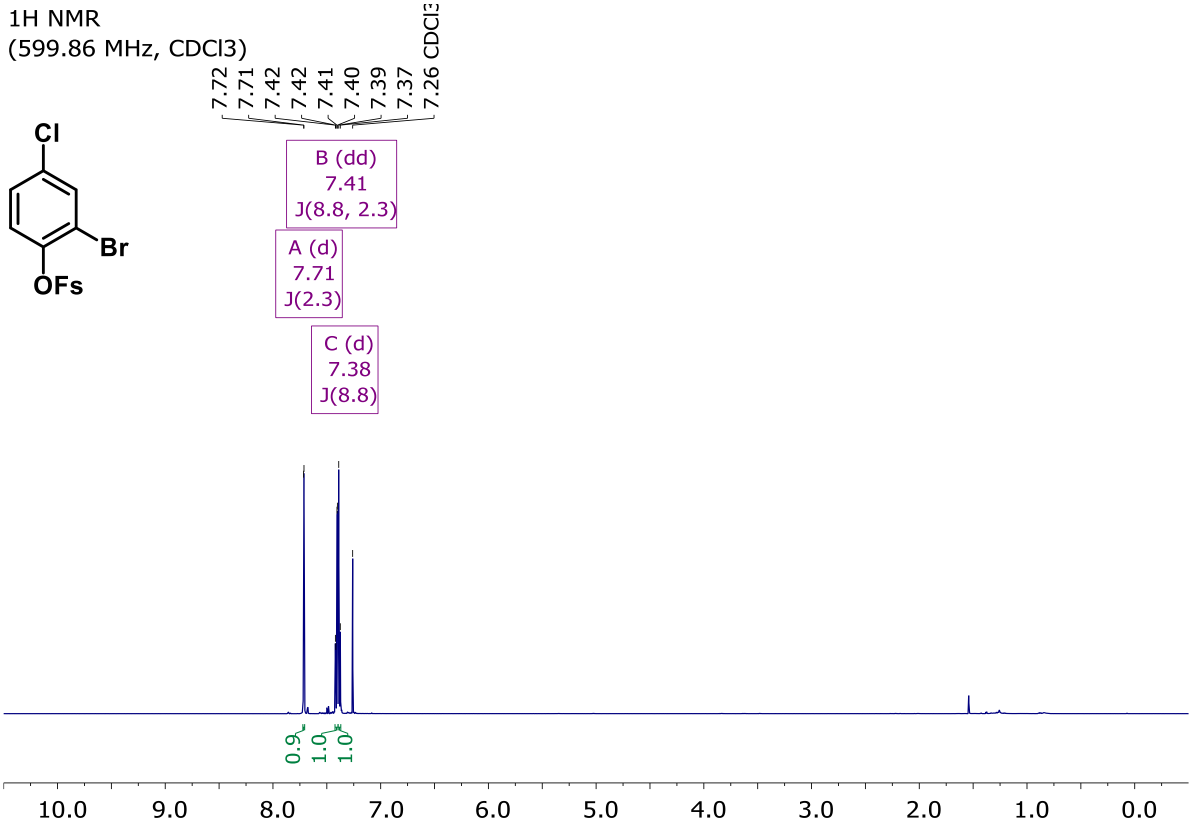

1H NMR
(599.86 MHz, CDCl3)
7.72
7.71
7.42
7.42
7.41
7.40
7.39
7.37
7.26 CDCl3
Cl
Br
OFs
B (dd)
7.41
J(8.8, 2.3)
A (d)
7.71
J(2.3)
C (d)
7.38
J(8.8)
0.9
1.0
1.0
10.0
9.0
8.0
7.0
6.0
5.0
4.0
3.0
2.0
1.0
0.0

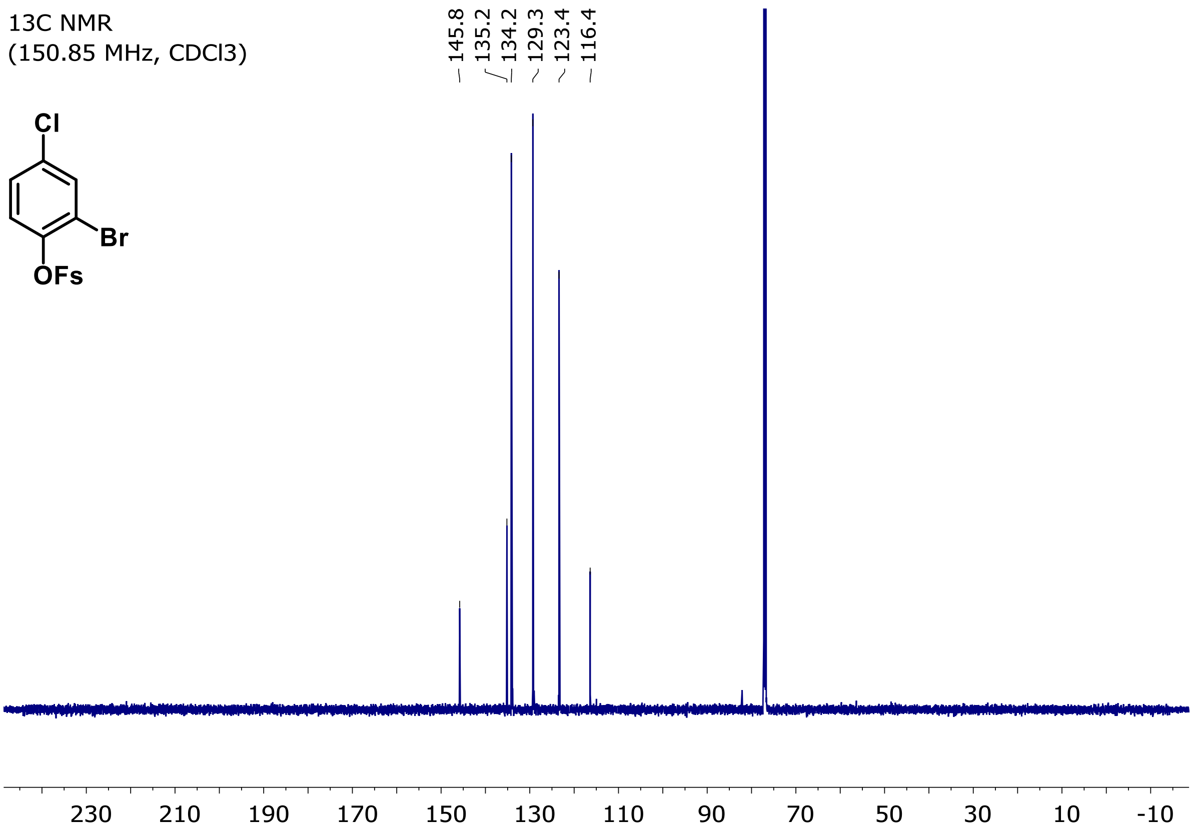
13C NMR
(150.85 MHz, CDCl3)
145.8
135.2
134.2
129.3
123.4
116.4
Cl
Br
OFs
230
210
190
170
150
130
110
90
70
50
30
10
-10

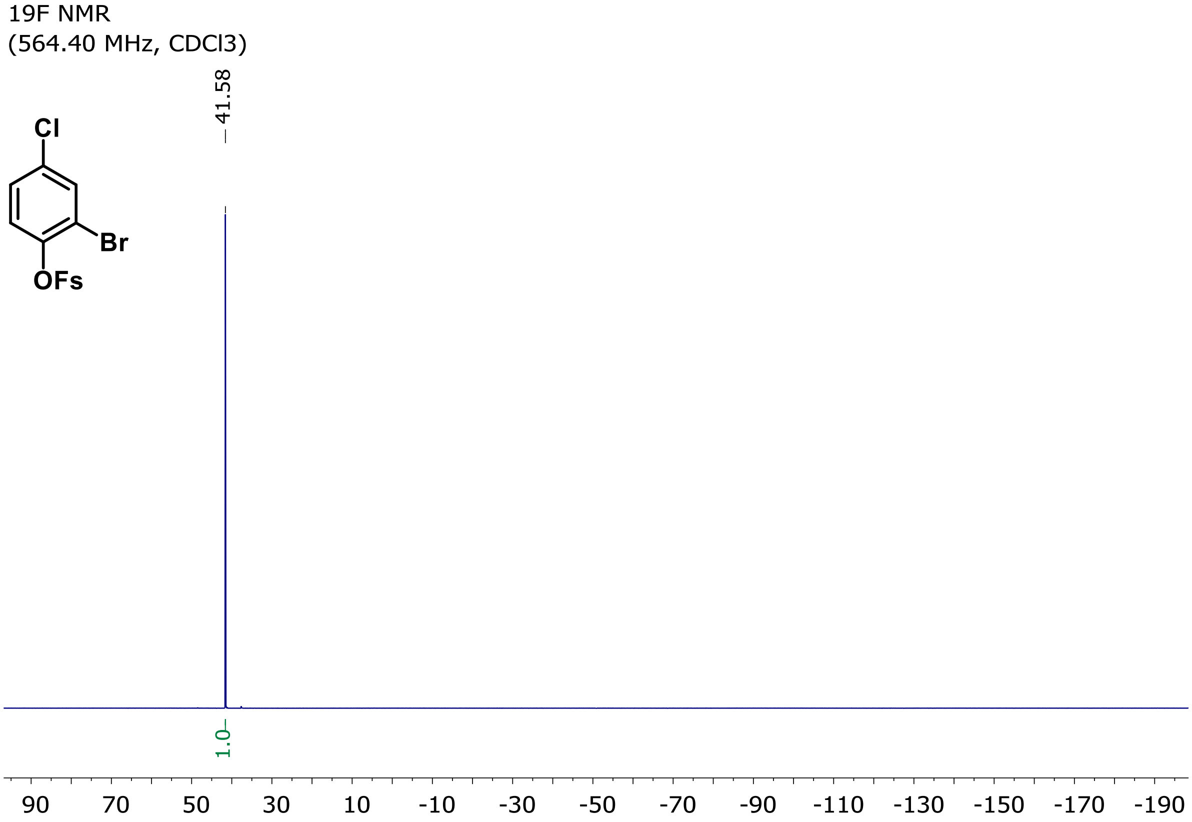
19F NMR
(564.40 MHz, CDCl3)
41.58
Cl
Br
OFs
1.0
90
70
50
30
10
-10
-30
-50
-70
-90
-110
-130
-150
-170
-190

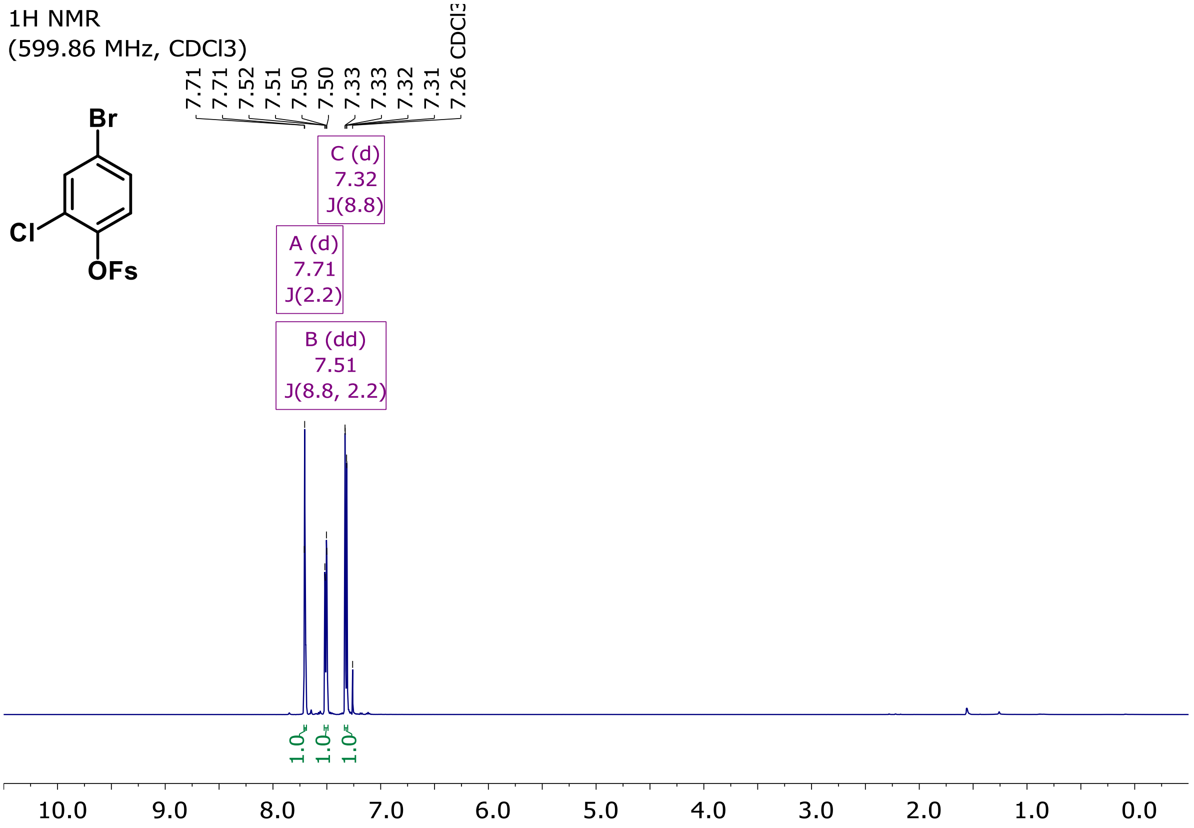
1H NMR
(599.86 MHz, CDCl3)
7.71
7.71
7.52
7.51
7.50
7.50
7.33
7.33
7.32
7.31
7.26 CDCl3
Br
Cl
OFs
C (d)
7.32
J(8.8)
A (d)
7.71
J(2.2)
B (dd)
7.51
J(8.8, 2.2)
1.0
1.0
1.0
10.0
9.0
8.0
7.0
6.0
5.0
4.0
3.0
2.0
1.0
0.0

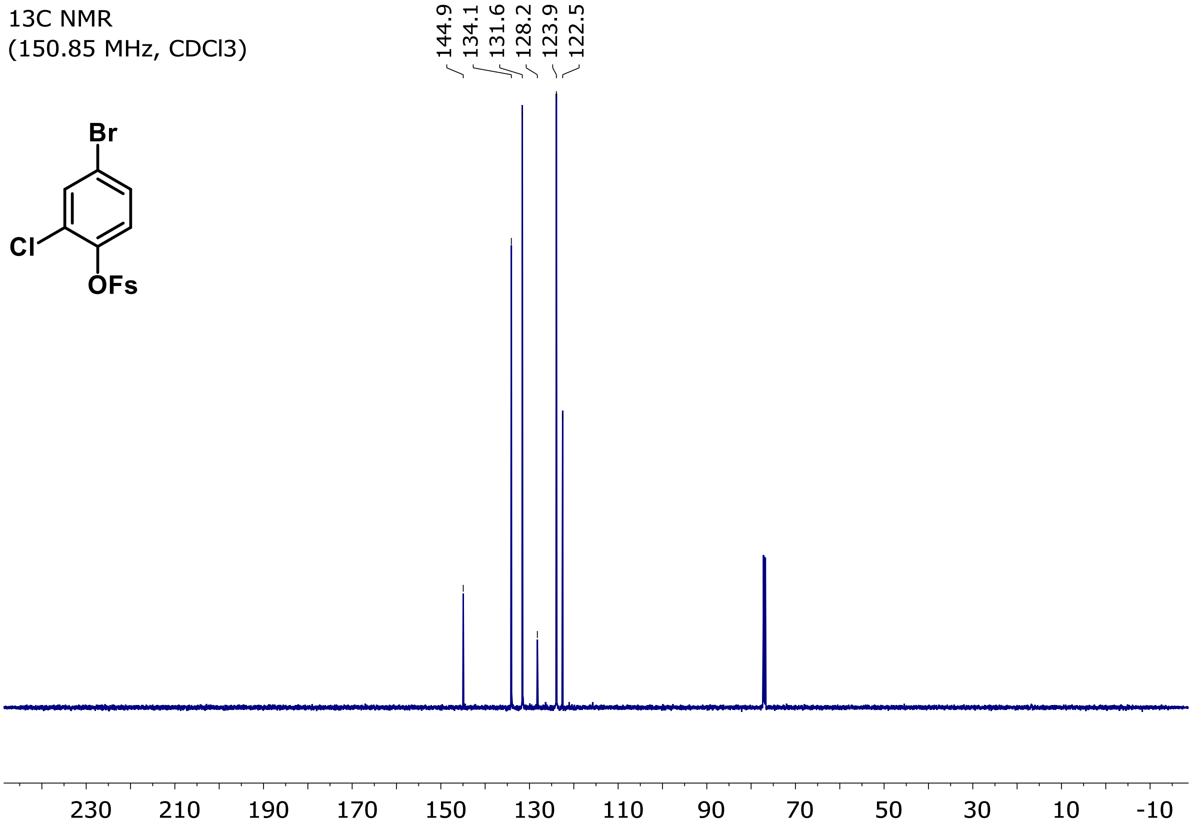
13C NMR
(150.85 MHz, CDCl3)
144.9
134.1
131.6
128.2
123.9
122.5
Br
Cl
OFs
230
210
190
170
150
130
110
90
70
50
30
10
-10

19F NMR
(564.40 MHz, CDCl3)

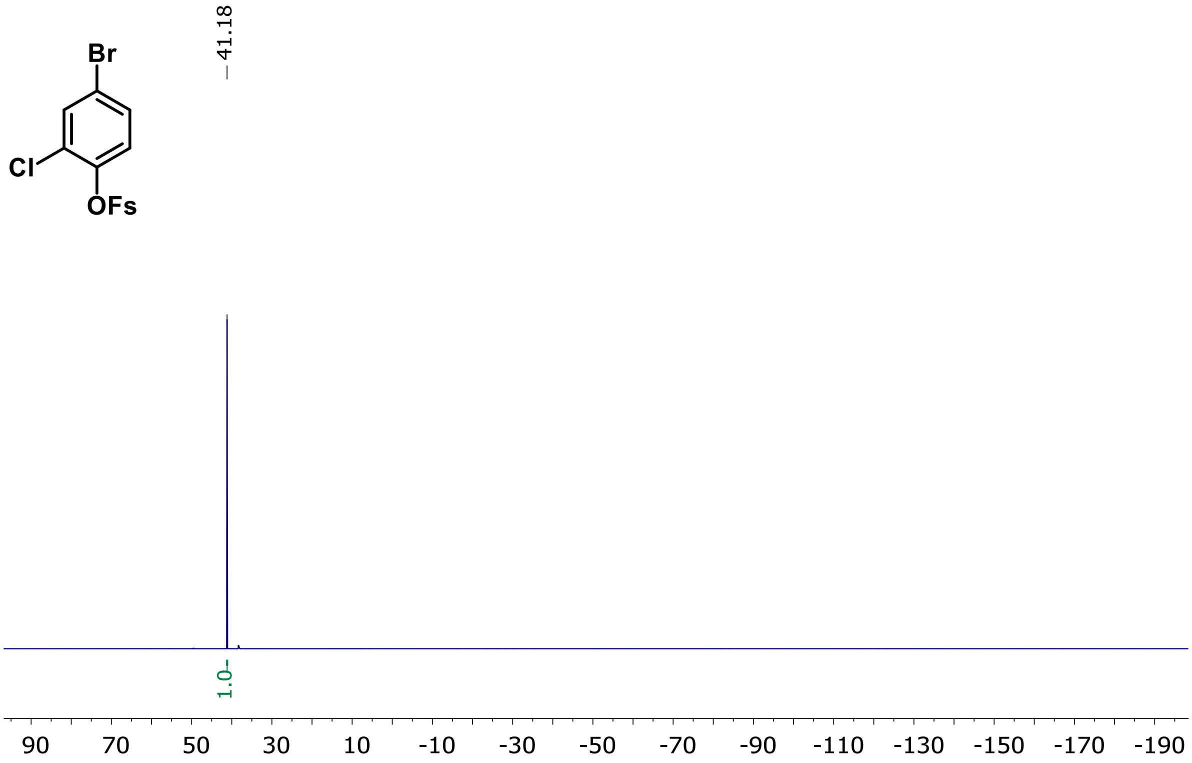

1H NMR
(599.86 MHz, CDCl3)

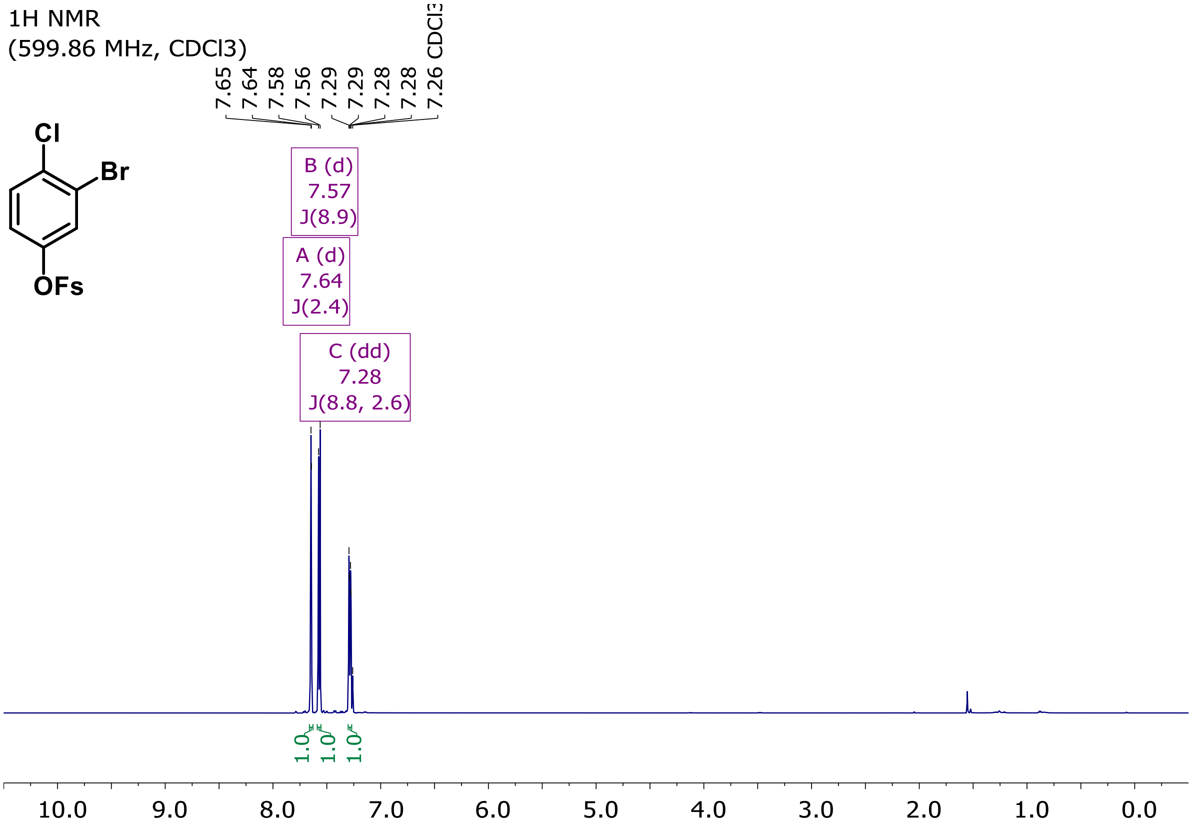

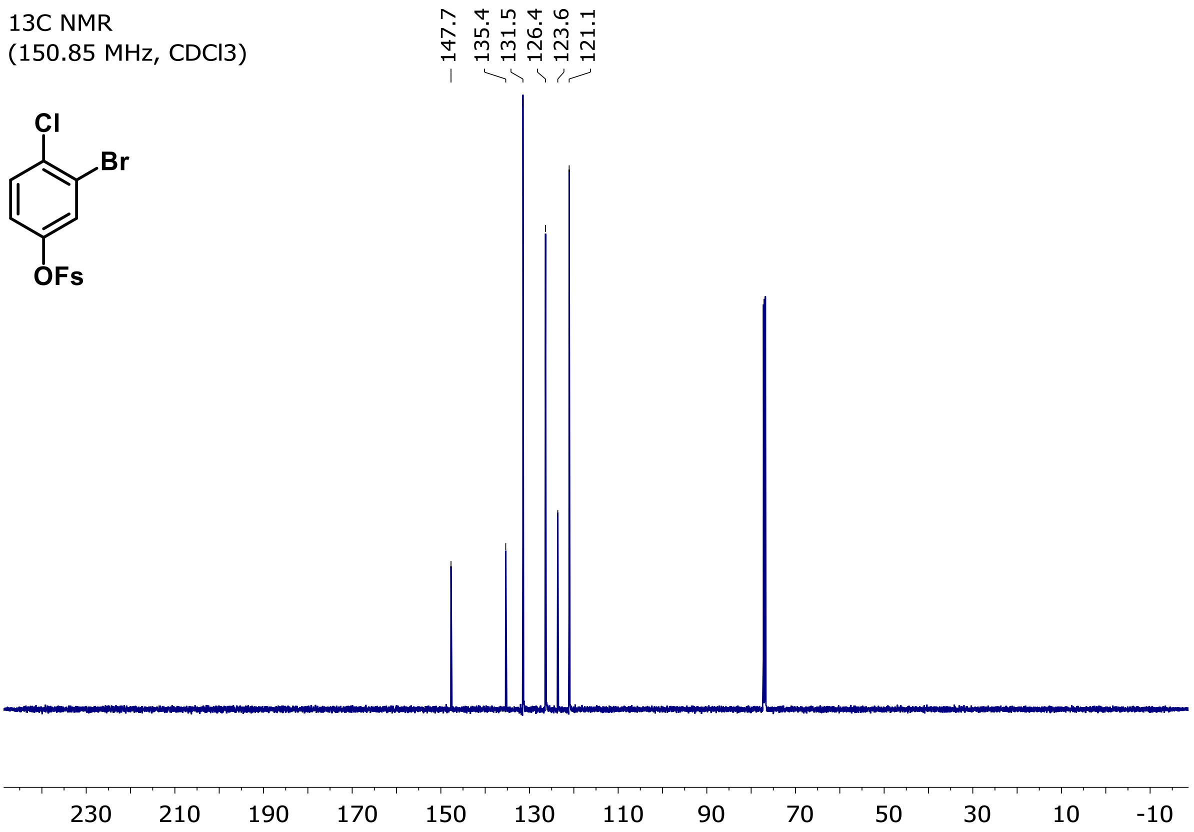
13C NMR
(150.85 MHz, CDCl3)
147.7
135.4
131.5
126.4
123.6
121.1
Cl
Br
OFs
230
210
190
170
150
130
110
90
70
50
30
10
-10

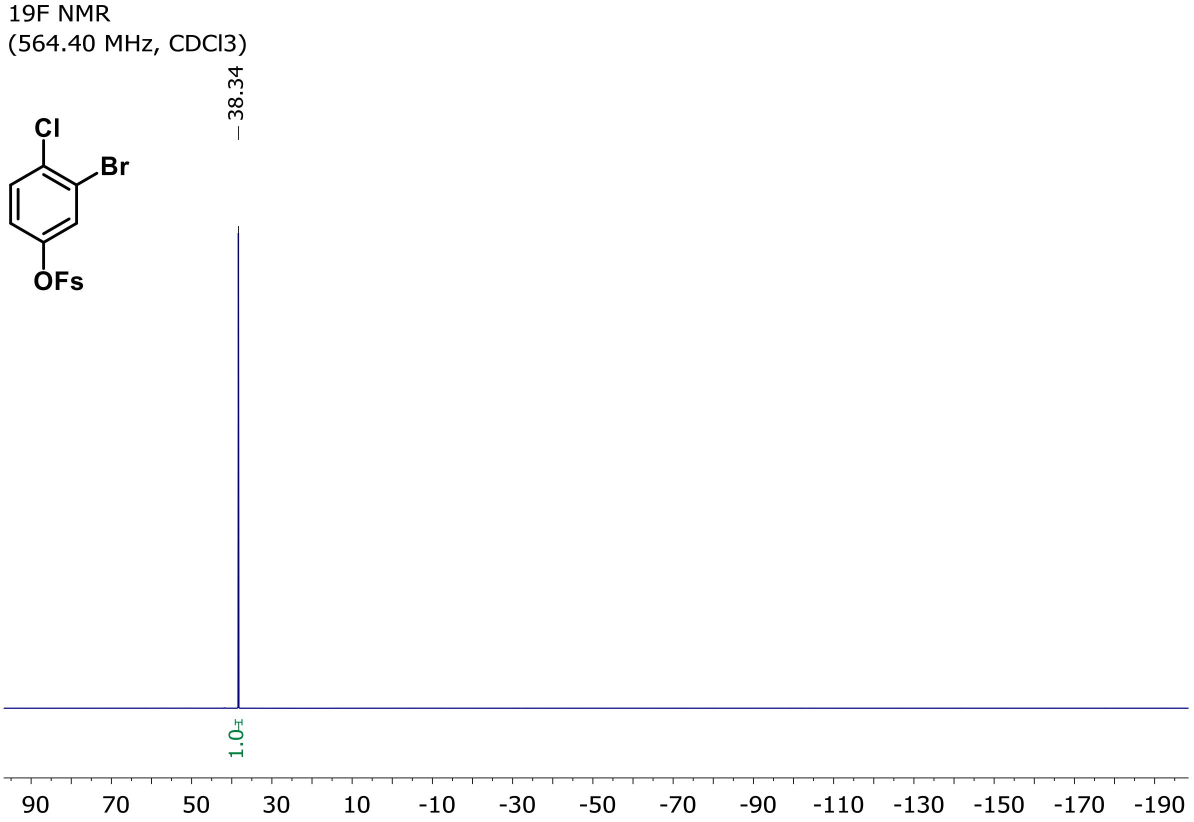
19F NMR
(564.40 MHz, CDCl3)
38.34
Cl
Br
OFs
1.0
90
70
50
30
10
-10
-30
-50
-70
-90
-110
-130
-150
-170
-190

Coupling products

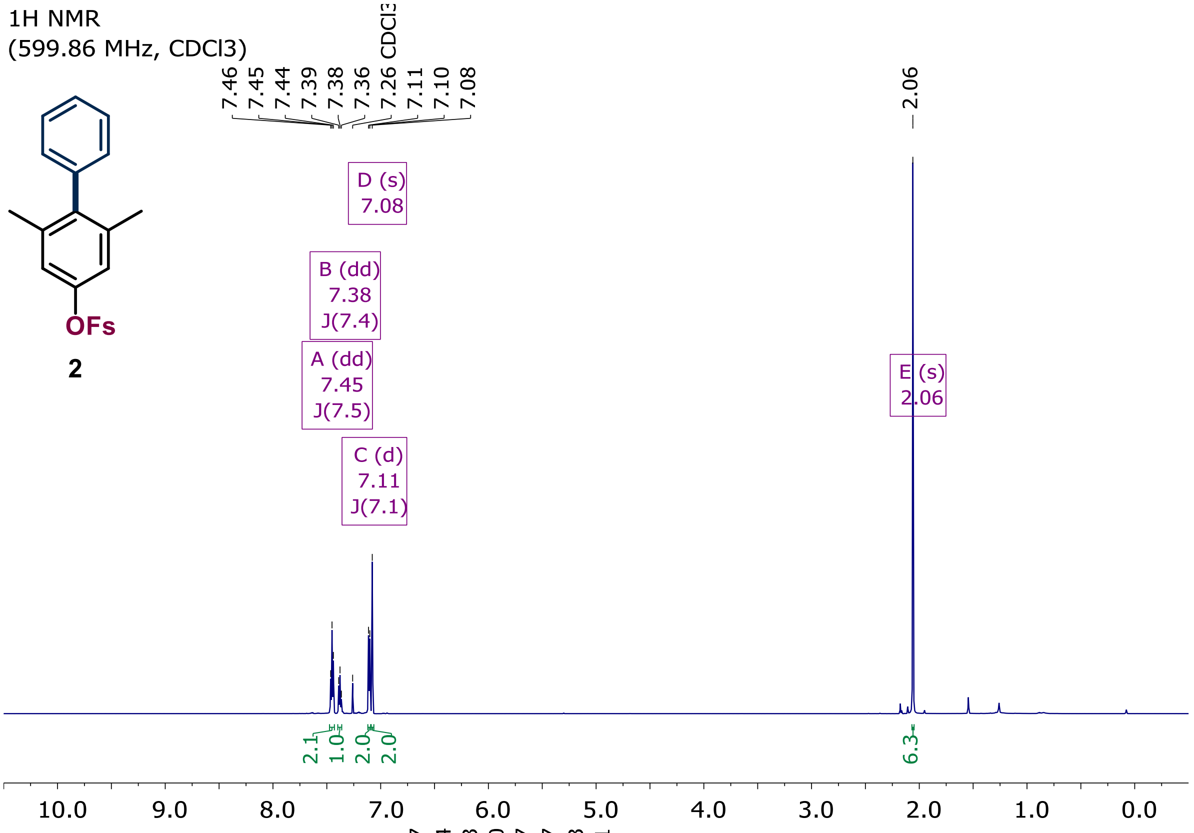

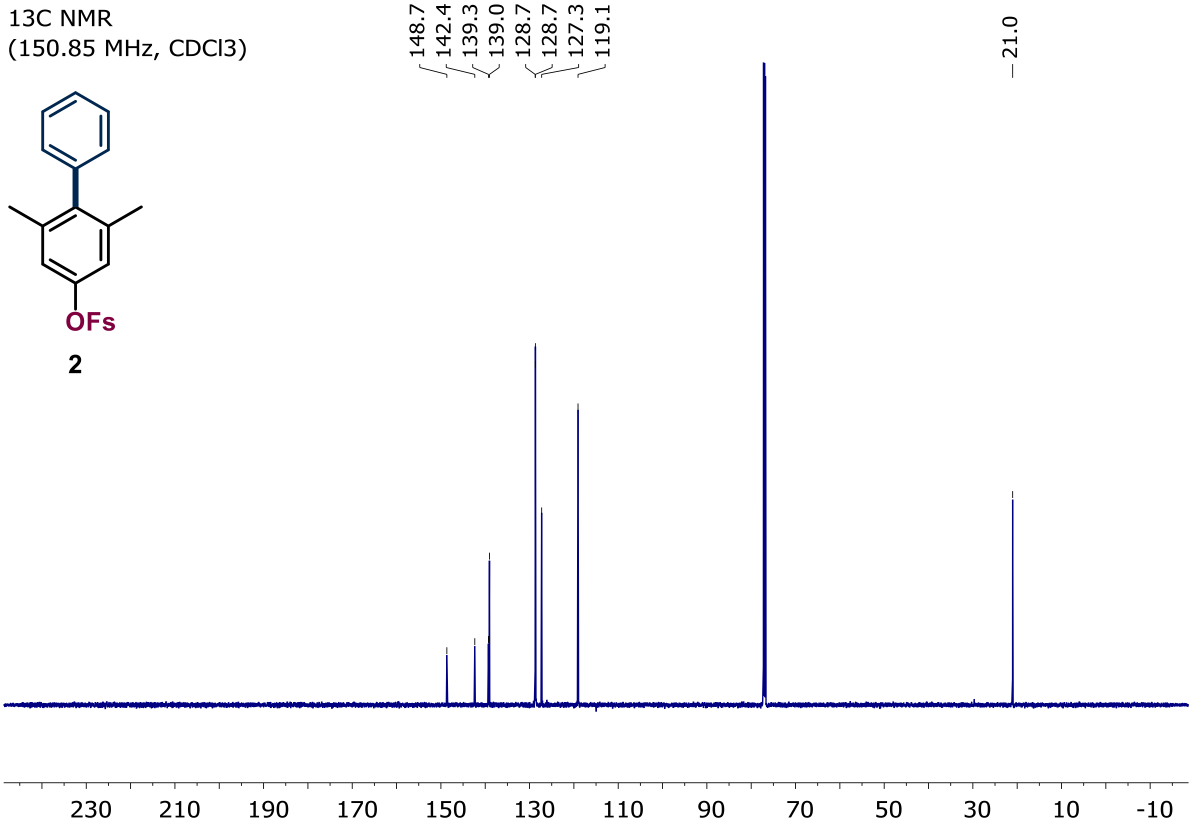

19F NMR
(564.40 MHz, CDCl3)

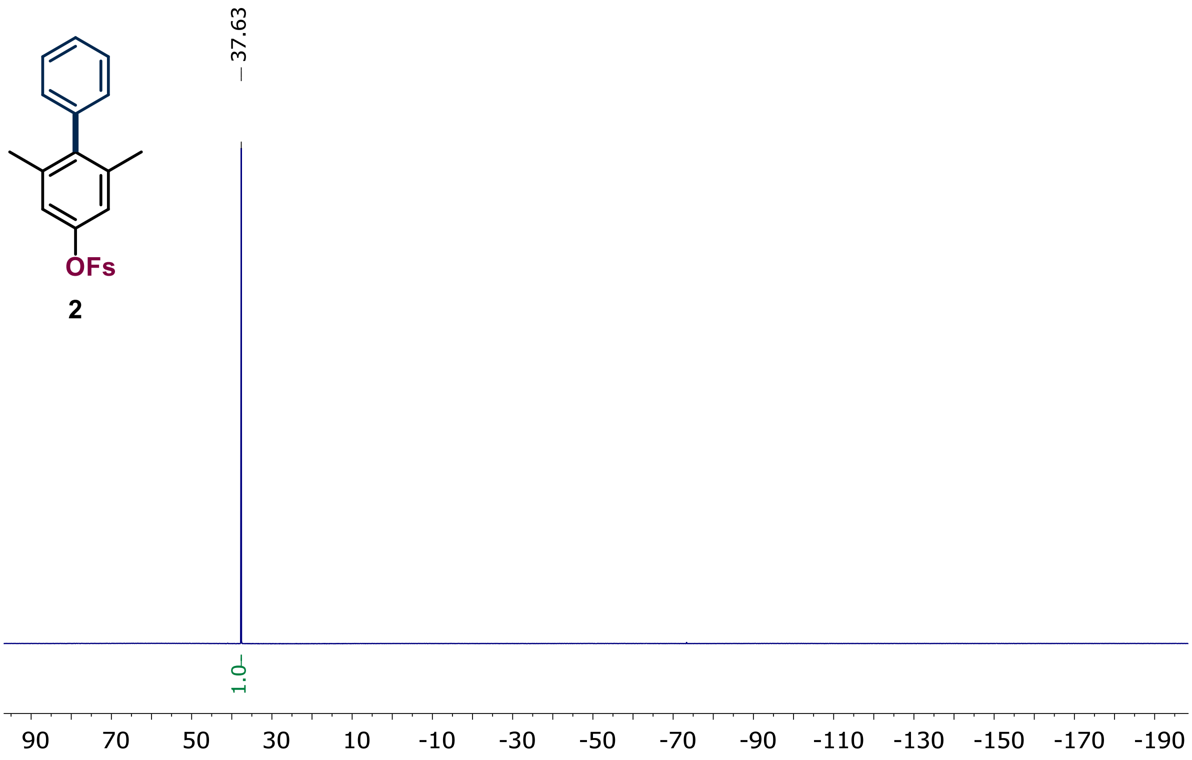

1H NMR
(599.86 MHz, CDCl3)

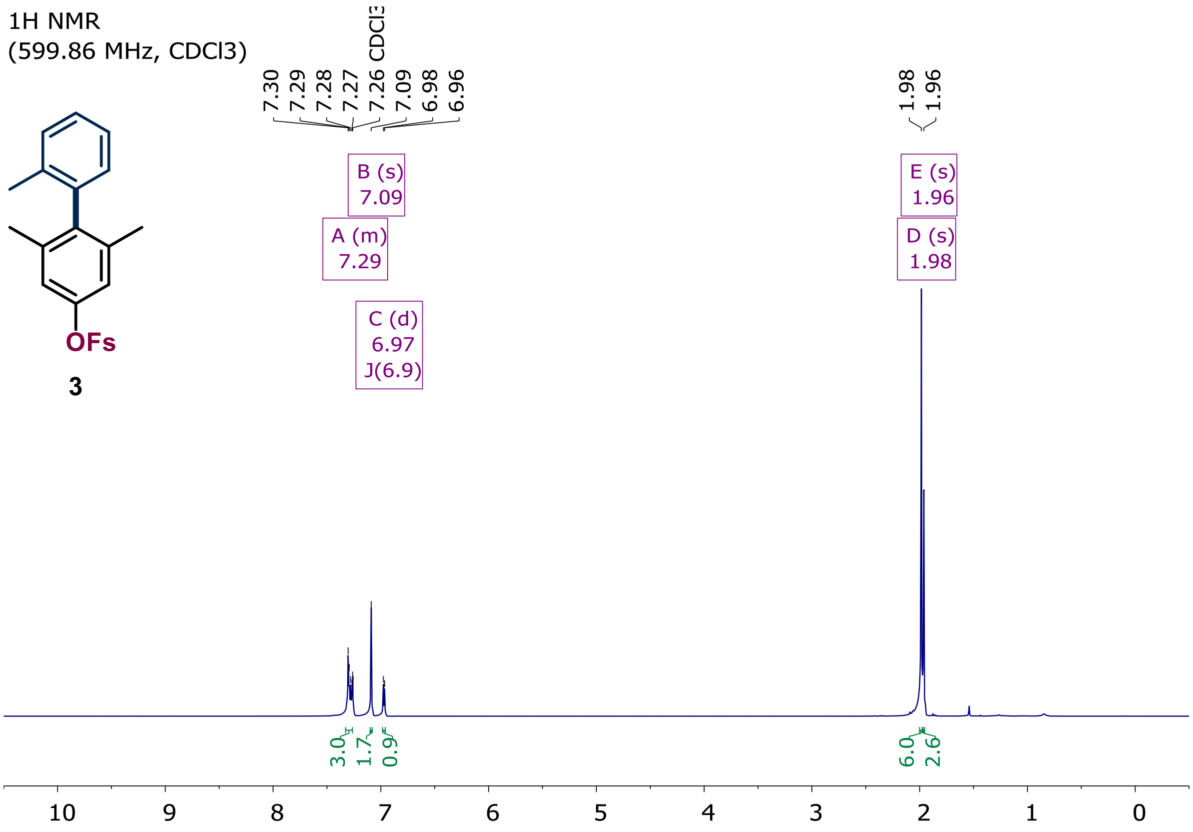

13C NMR
(150.85 MHz, CDCl3)

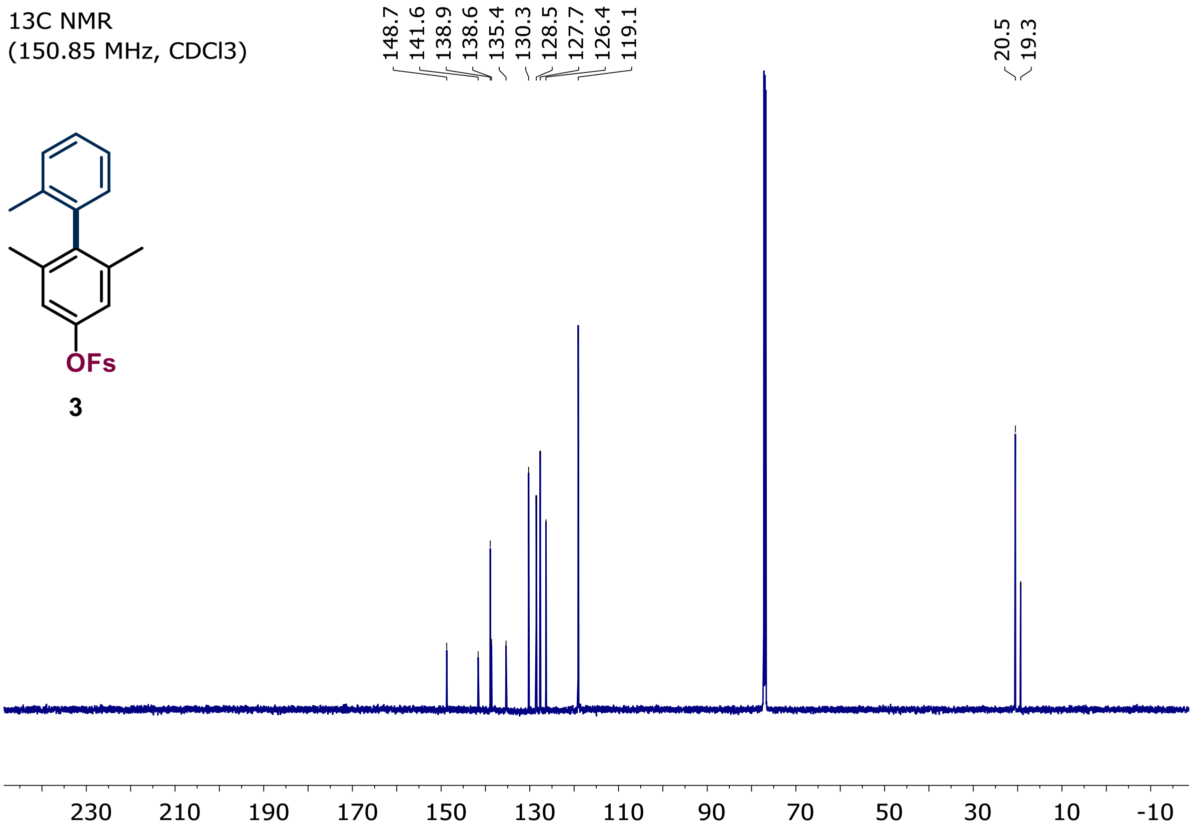

19F NMR
(564.40 MHz, CDCl3)

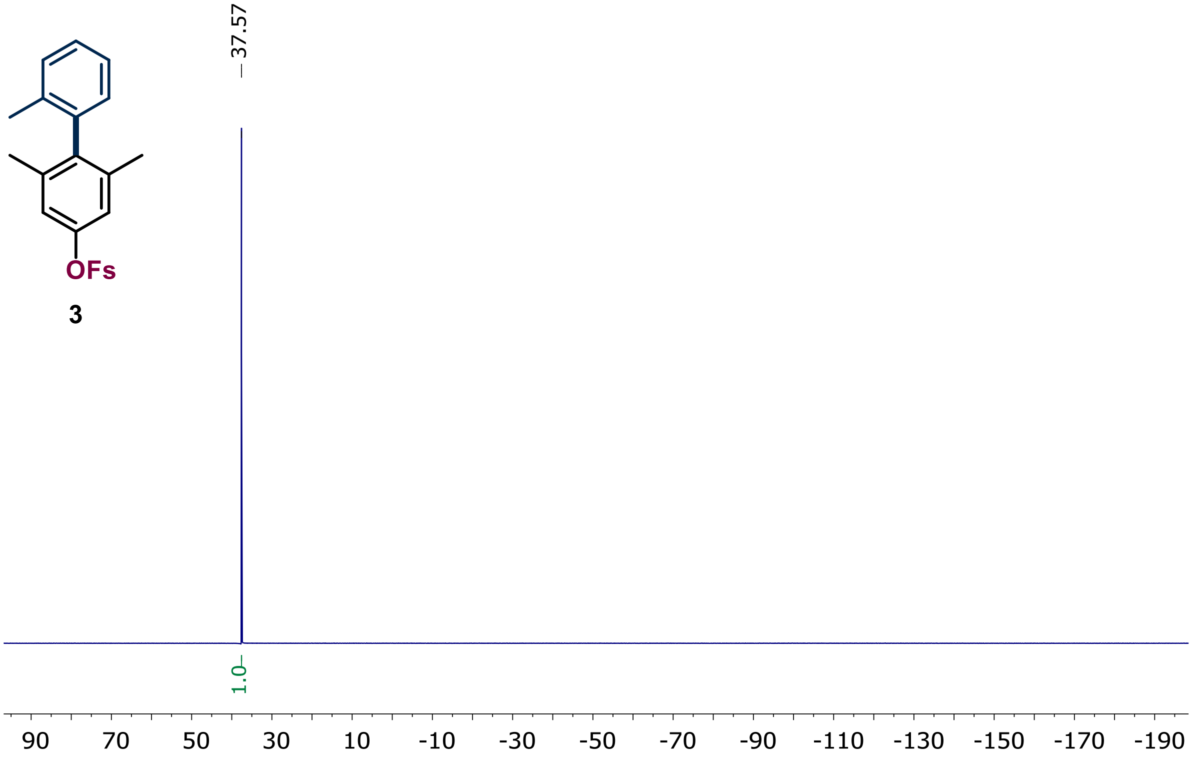

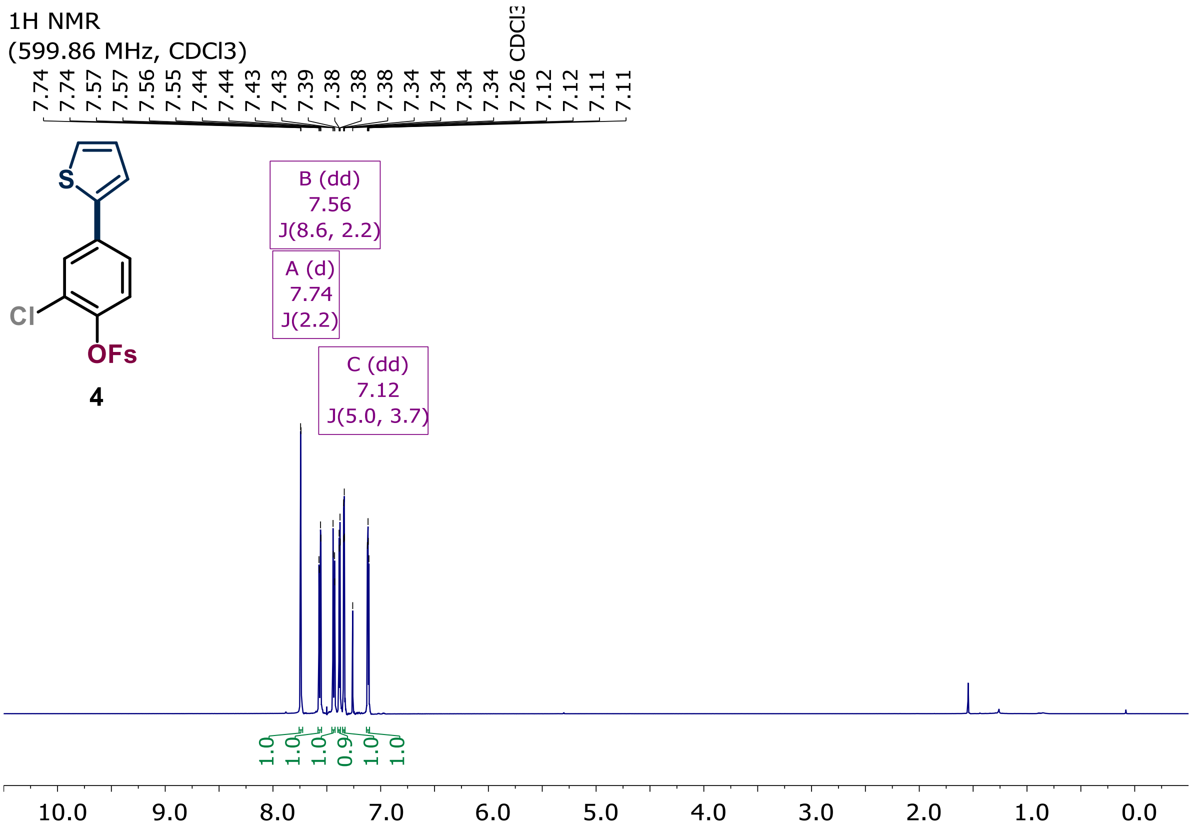

1H NMR
(599.86 MHz, CDCl3)
7.74
7.74
7.57
7.57
7.56
7.55
7.44
7.44
7.43
7.43
7.39
7.38
7.38
7.38
7.34
7.34
7.34
7.34
7.26 CDCl3
7.12
7.12
7.11
7.11
S
Cl
OFs
4
B (dd)
7.56
J(8.6, 2.2)
A (d)
7.74
J(2.2)
C (dd)
7.12
J(5.0, 3.7)
1.0
1.0
1.0
0.9
1.0
1.0
10.0
9.0
8.0
7.0
6.0
5.0
4.0
3.0
2.0
1.0
0.0

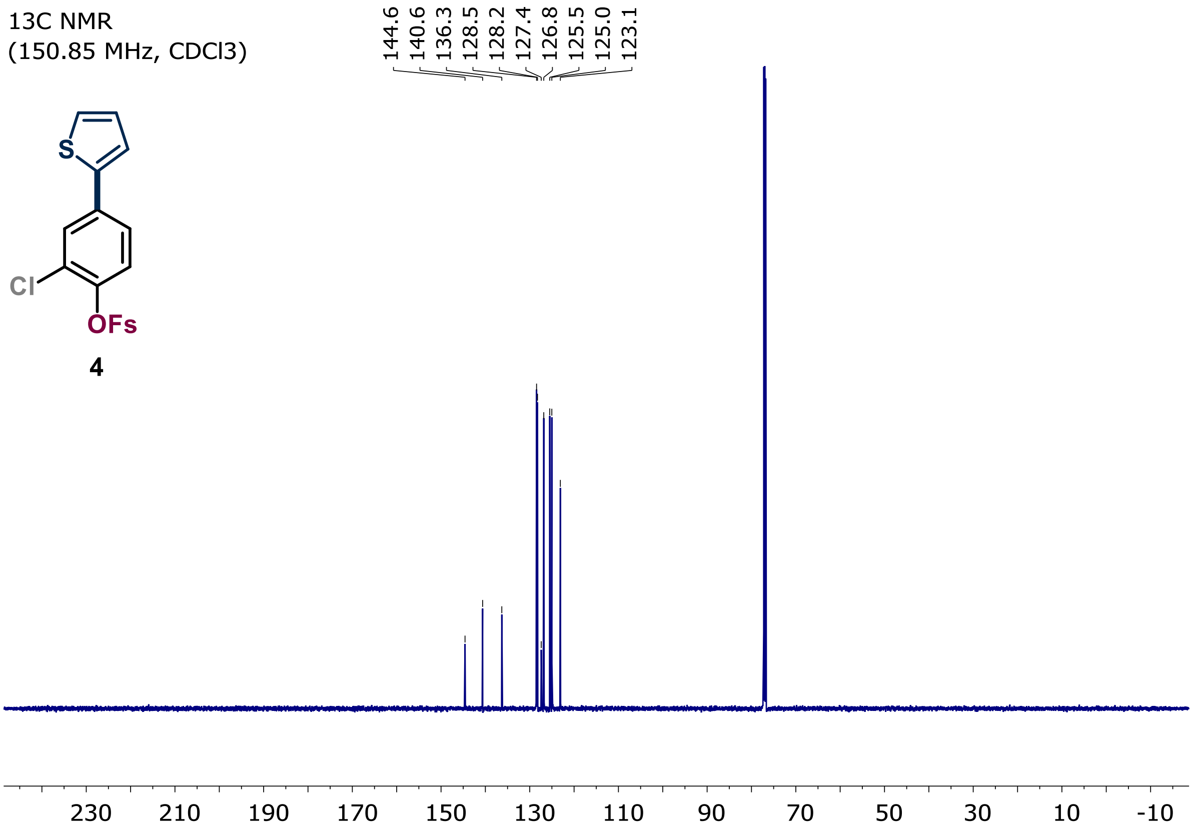

13C NMR
(150.85 MHz, CDCl3)
144.6
140.6
136.3
128.5
128.2
127.4
126.8
125.5
125.0
123.1
S
Cl
OFs
4
230
210
190
170
150
130
110
90
70
50
30
10
-10

19F NMR
(564.40 MHz, CDCl3)

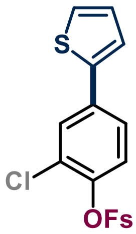

4

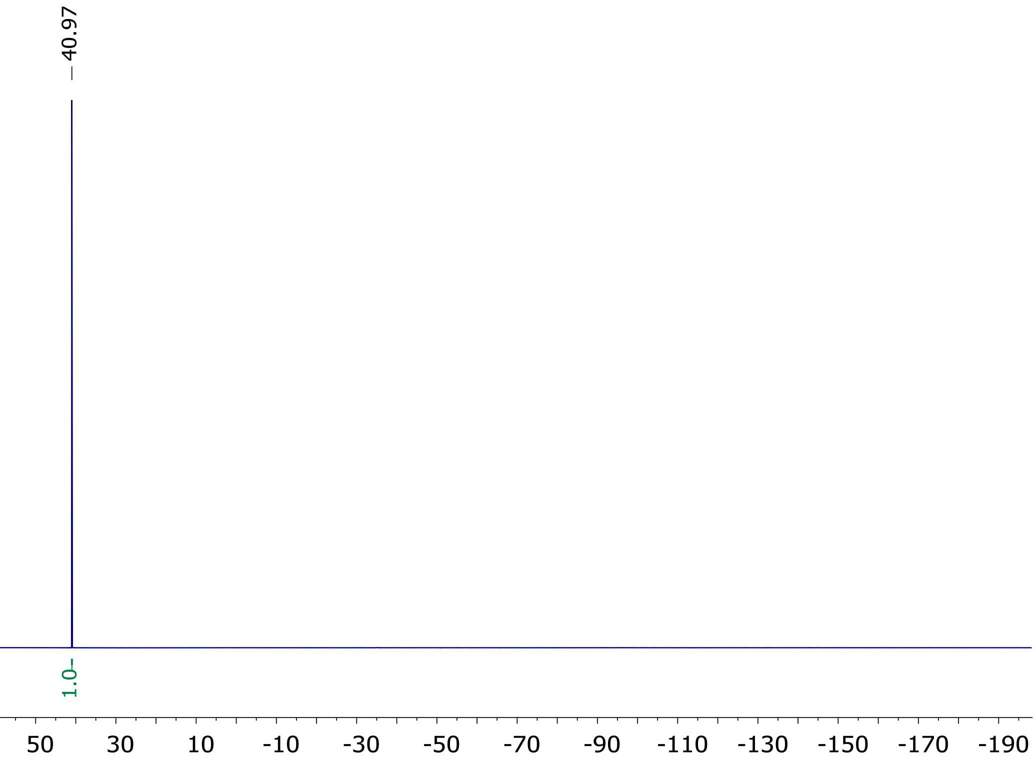

1H NMR
(599.86 MHz, CDCl3)

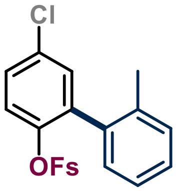

5

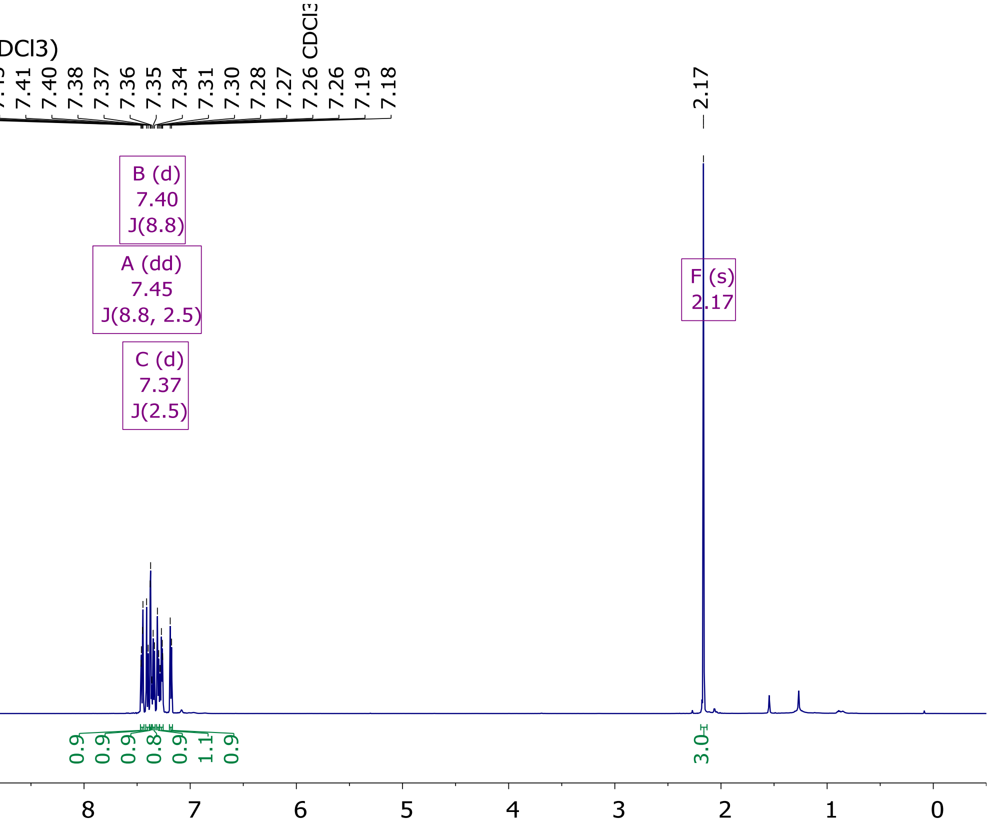

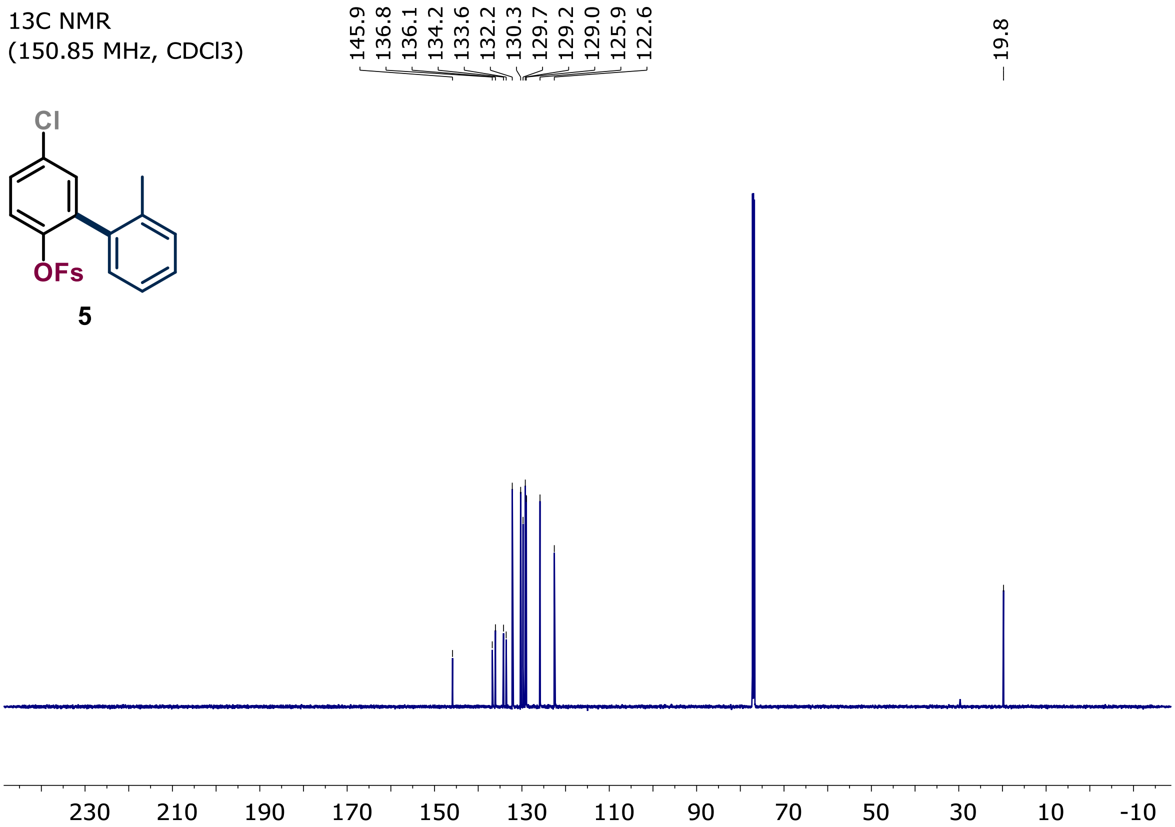

13C NMR
(150.85 MHz, CDCl3)
145.9
136.8
136.1
134.2
133.6
132.2
130.3
129.7
129.2
129.0
125.9
122.6
19.8
Cl
OFs
5
230
210
190
170
150
130
110
90
70
50
30
10
-10

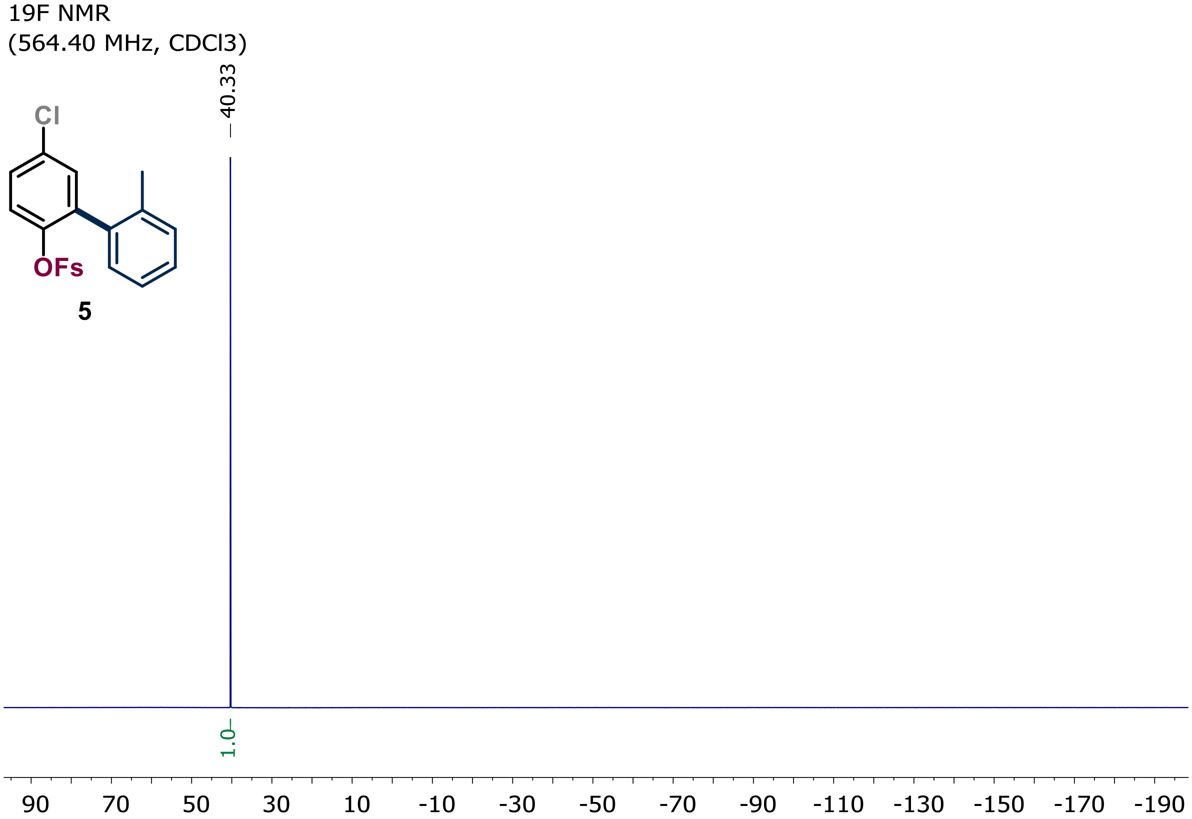

19F NMR
(564.40 MHz, CDCl3)
-40.33
Cl
OFs
5
1.0
90
70
50
30
10
-10
-30
-50
-70
-90
-110
-130
-150
-170
-190

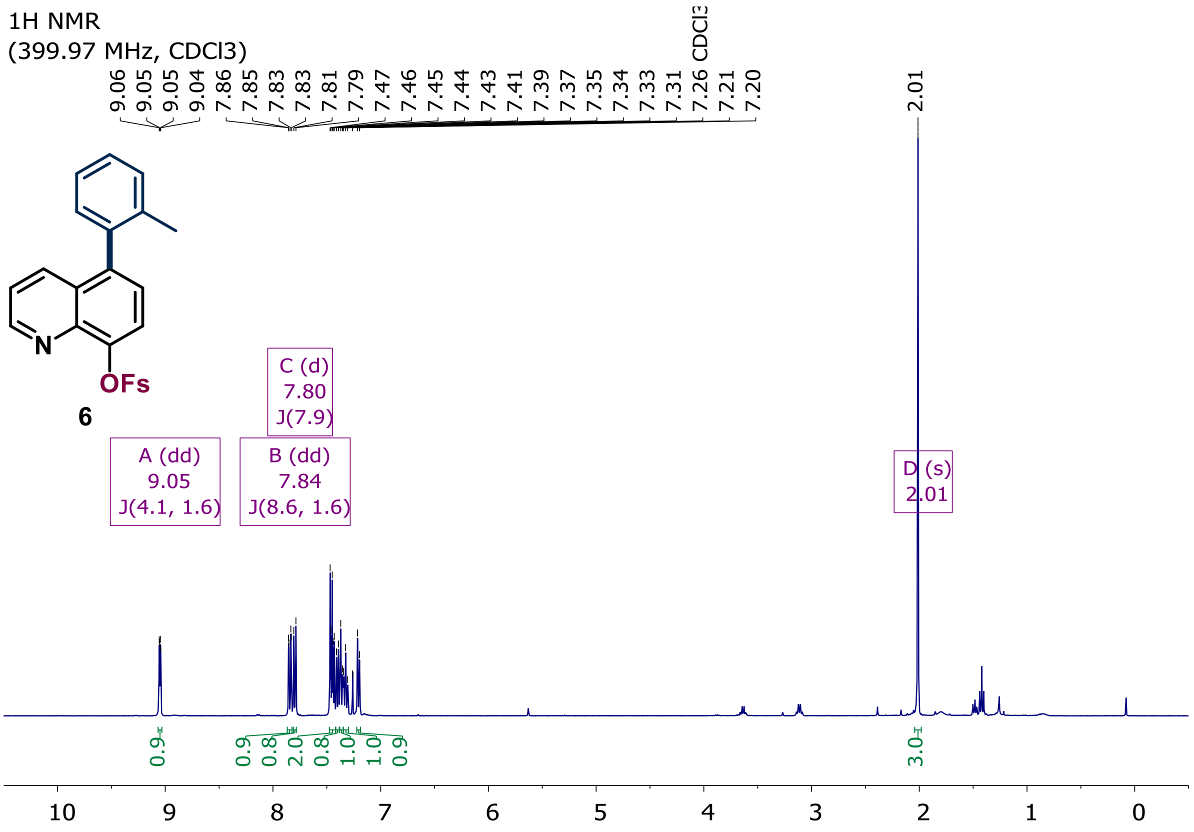
1H NMR
(399.97 MHz, CDCl3)
9.06 9.05 9.05 9.04 7.86 7.85 7.83 7.83 7.81 7.79 7.47 7.46 7.45 7.44 7.43 7.41 7.39 7.37 7.35 7.34 7.33 7.31 7.26 CDCl3 7.21 7.20
2.01
N
OFs
6
C (d) 7.80 J(7.9)
A (dd) 9.05 J(4.1, 1.6)
B (dd) 7.84 J(8.6, 1.6)
D (s) 2.01
0.9 0.9 0.8 2.0 0.8 1.0 1.0 0.9 3.0
10 9 8 7 6 5 4 3 2 1 0

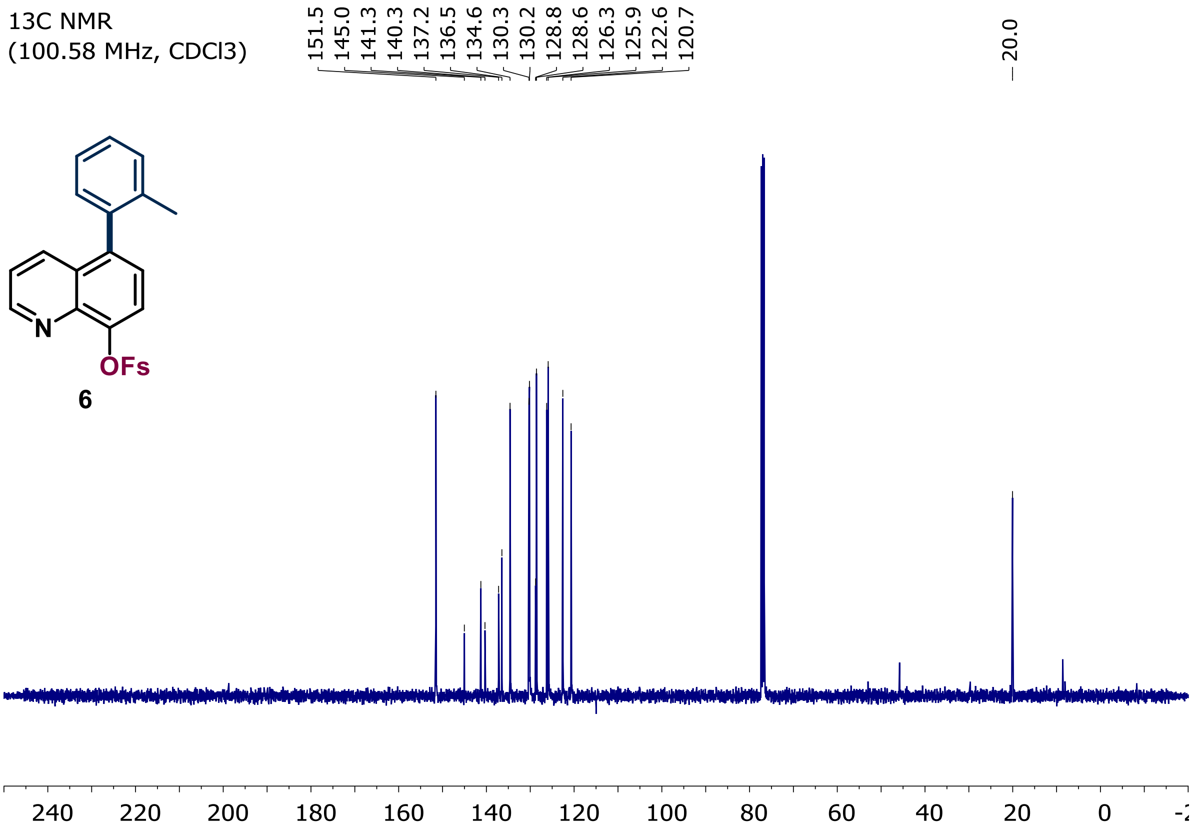
13C NMR
(100.58 MHz, CDCl3)
151.5 145.0 141.3 140.3 137.2 136.5 134.6 130.3 130.2 128.8 128.6 126.3 125.9 122.6 120.7
20.0
N
OFs
6
240 220 200 180 160 140 120 100 80 60 40 20 0

19F NMR
(376.33 MHz, CDCl3)

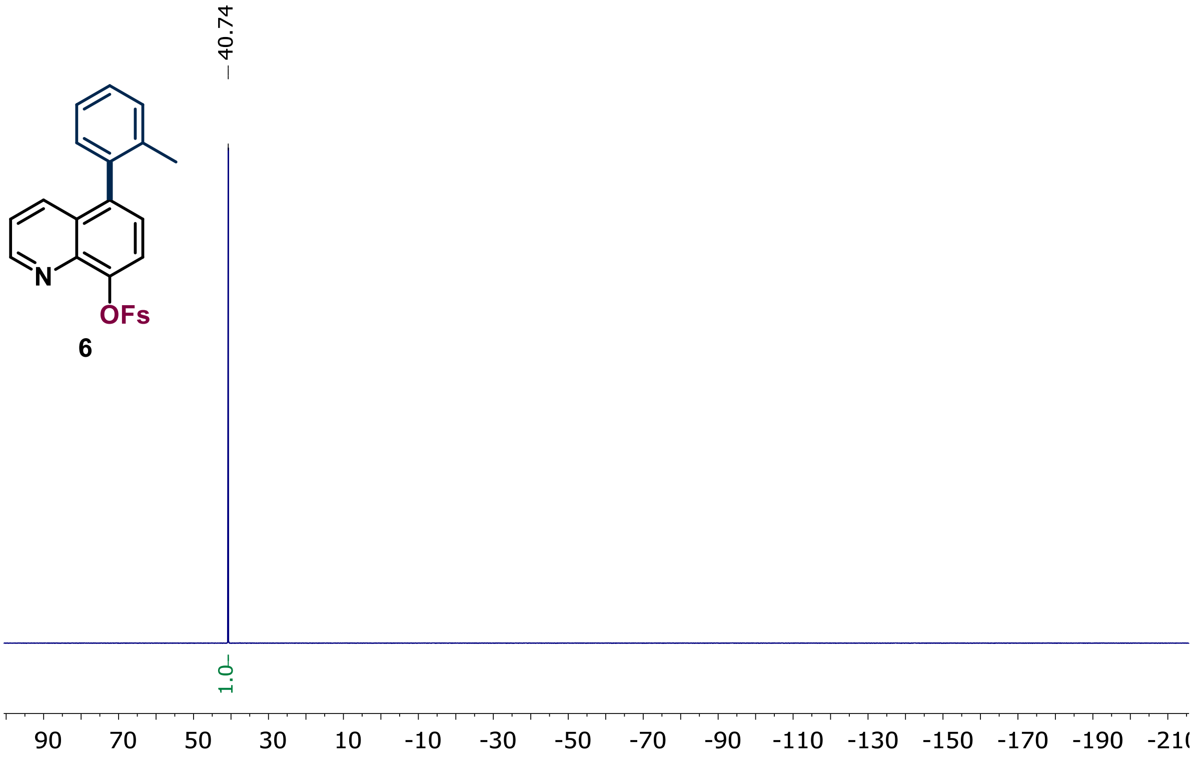

1H NMR
(599.86 MHz, CDCl3)

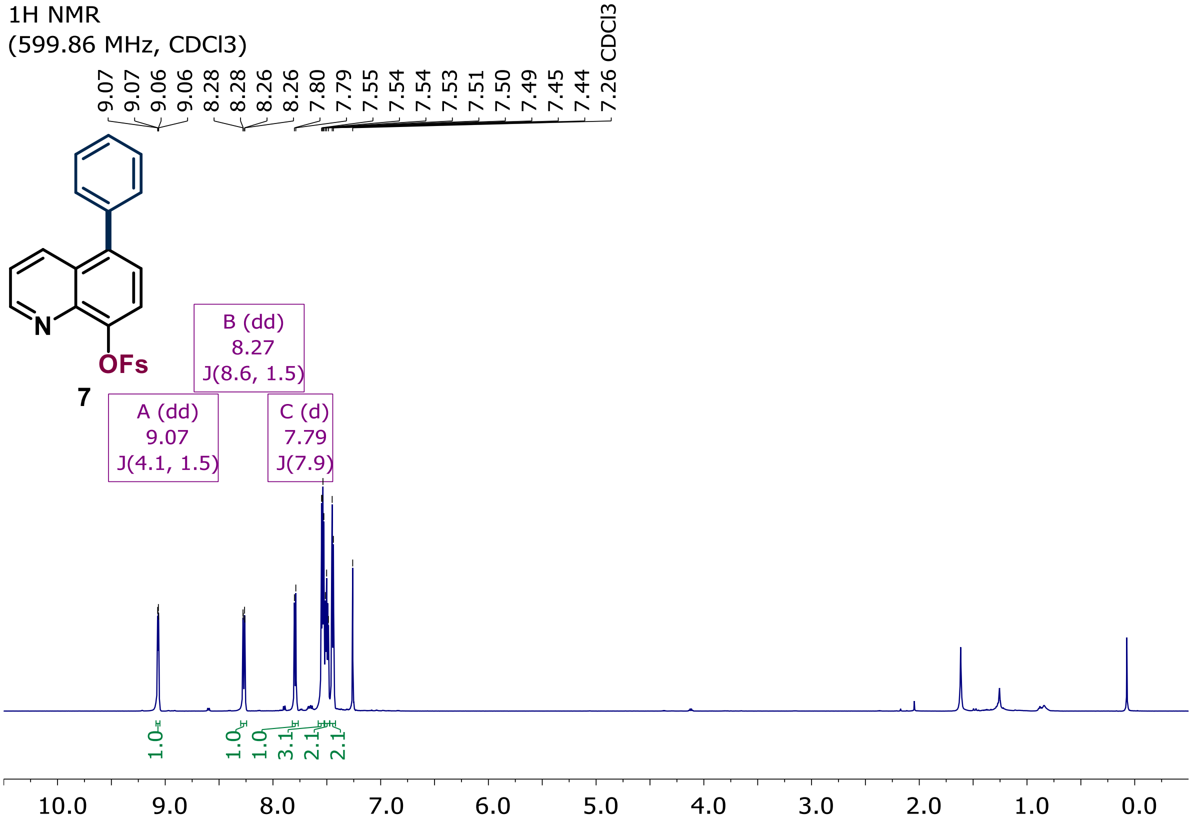

13C NMR
(150.85 MHz, CDCl3)

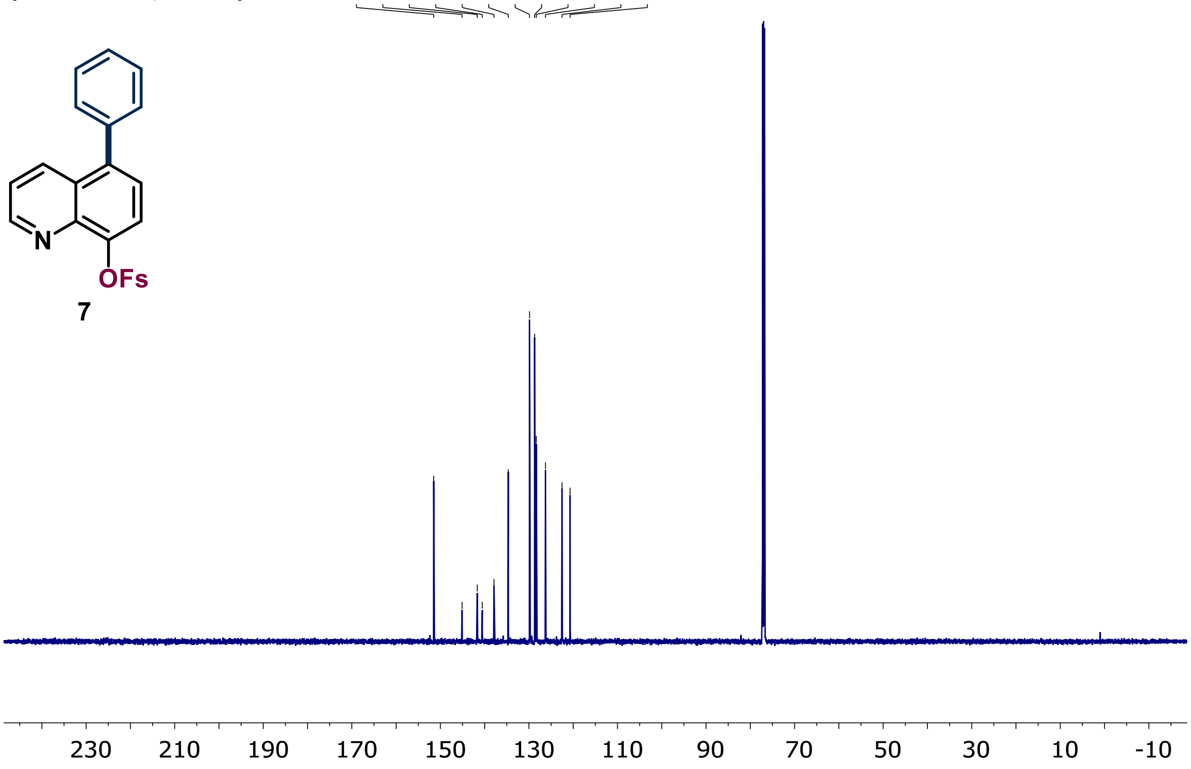

7

19F NMR
(564.40 MHz, CDCl3)

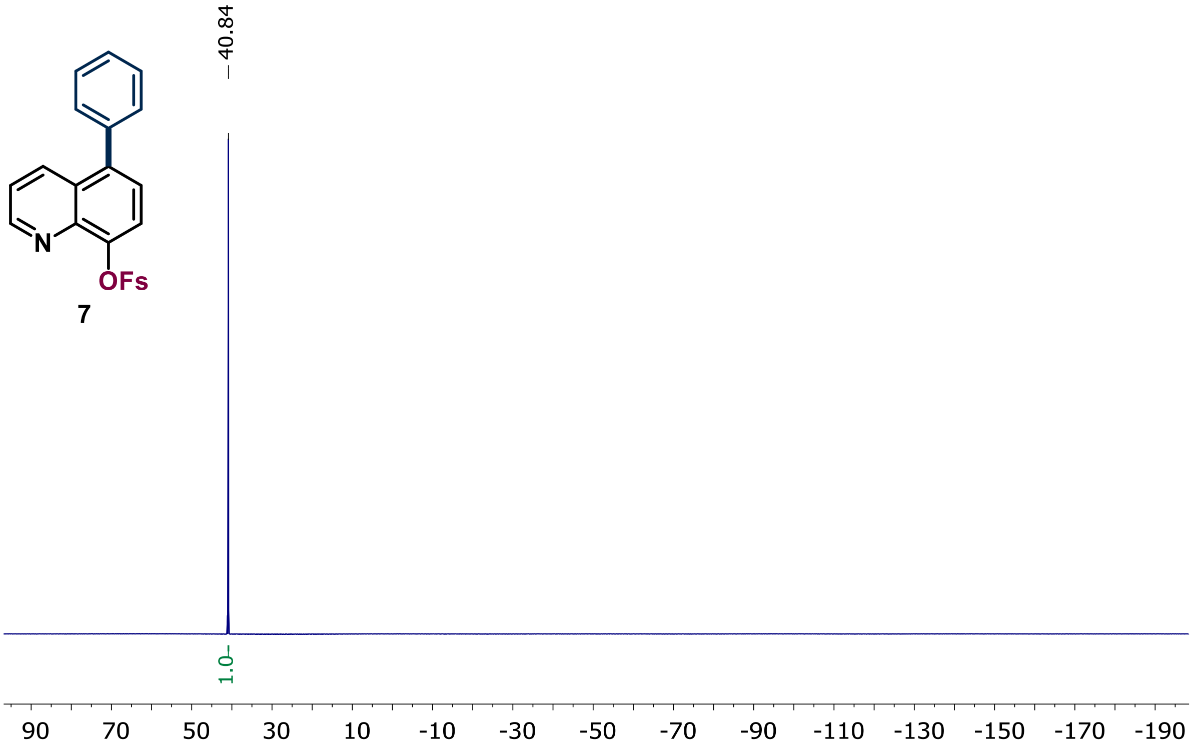

7

1H NMR
(399.97 MHz, CDCl3)

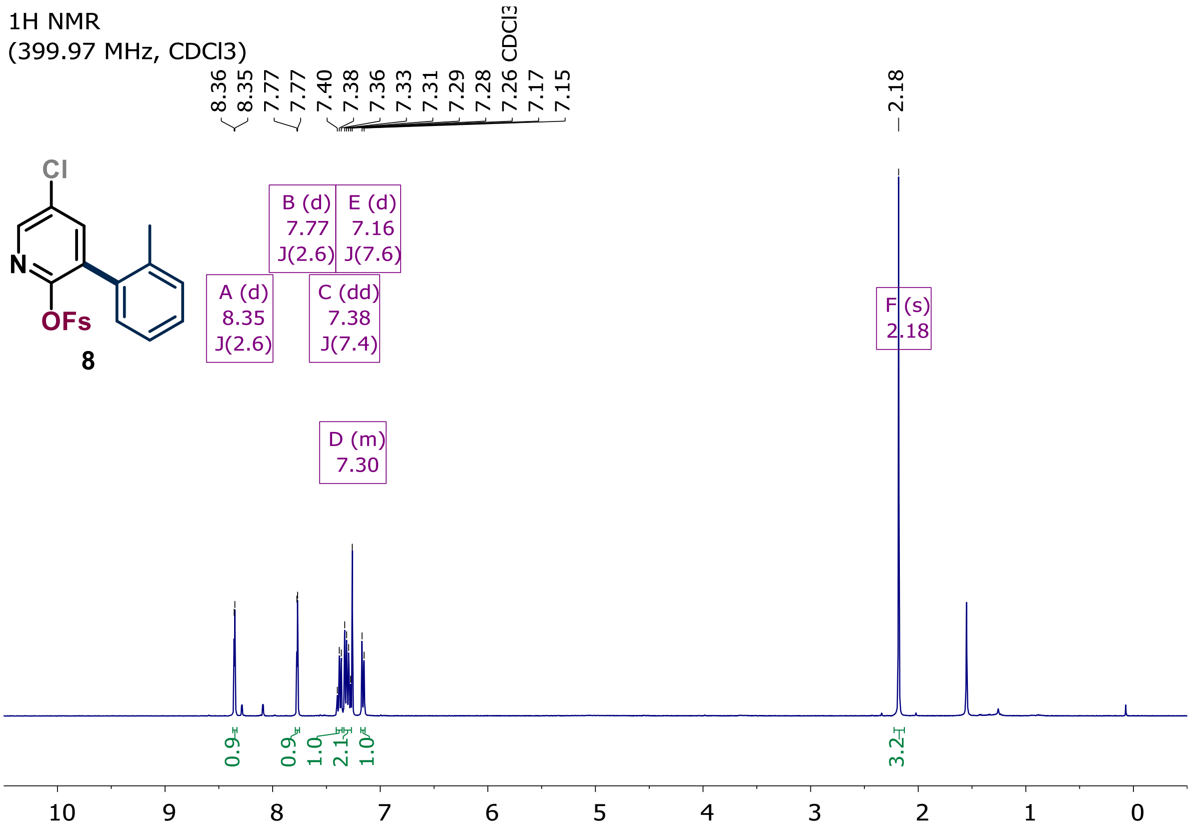

13C NMR
(100.58 MHz, CDCl3)

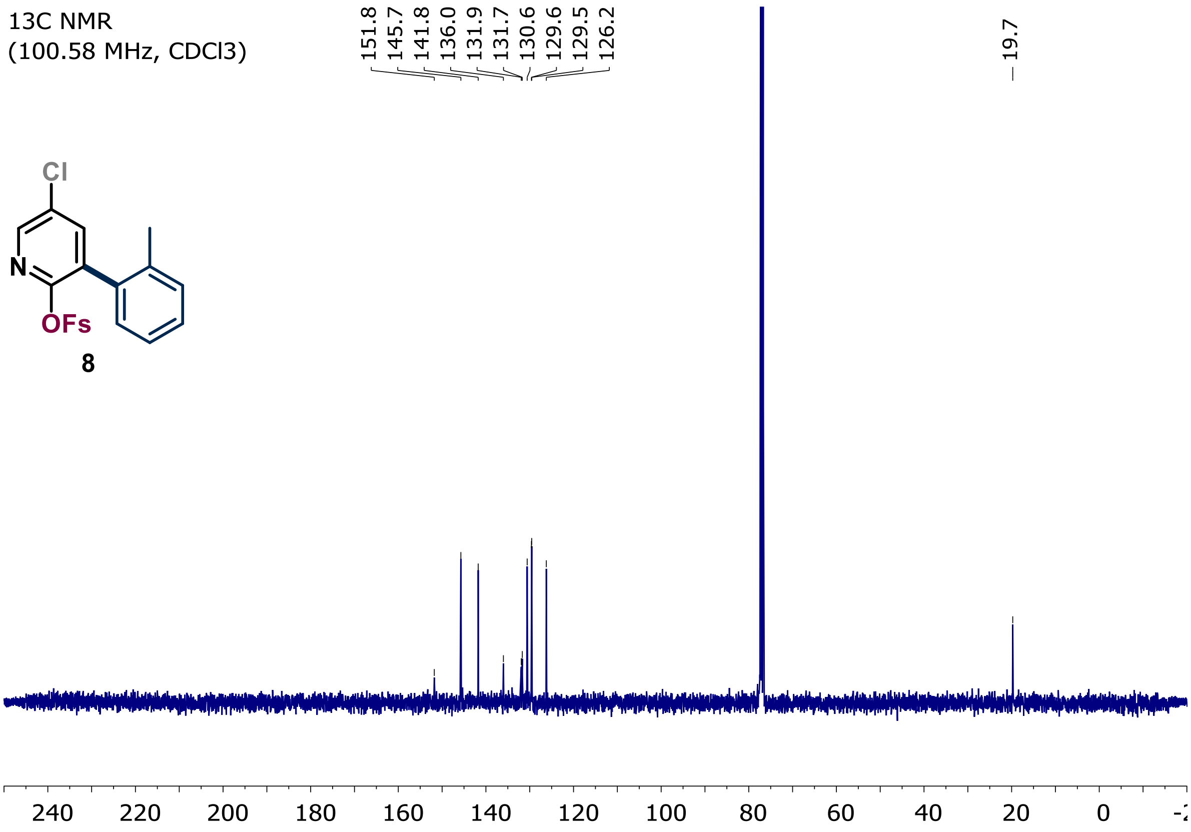

19F NMR
(376.33 MHz, CDCl3)

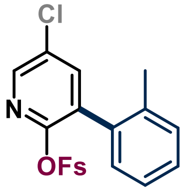

8

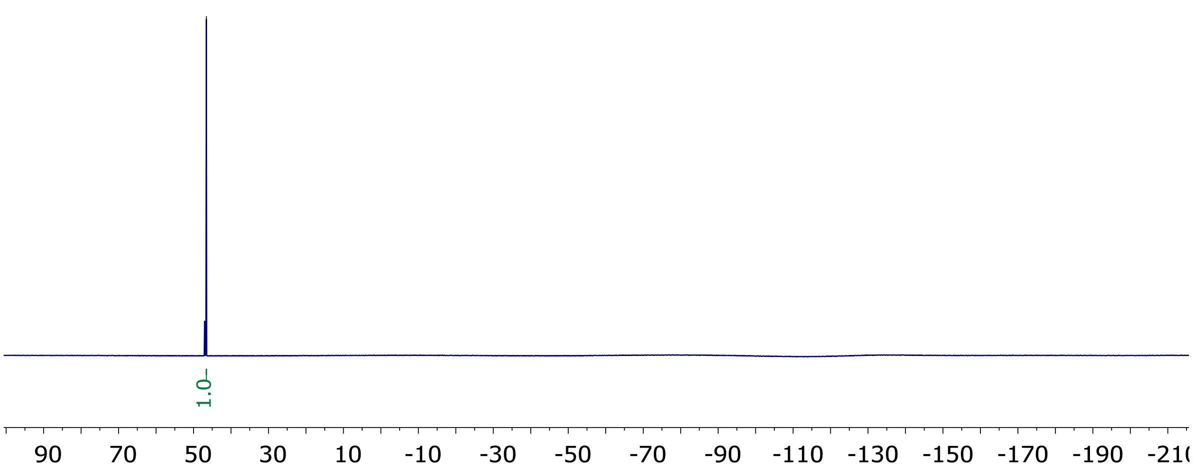

1H NMR
(599.86 MHz, CDCl3)

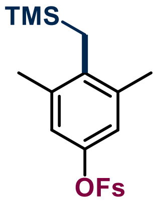

11

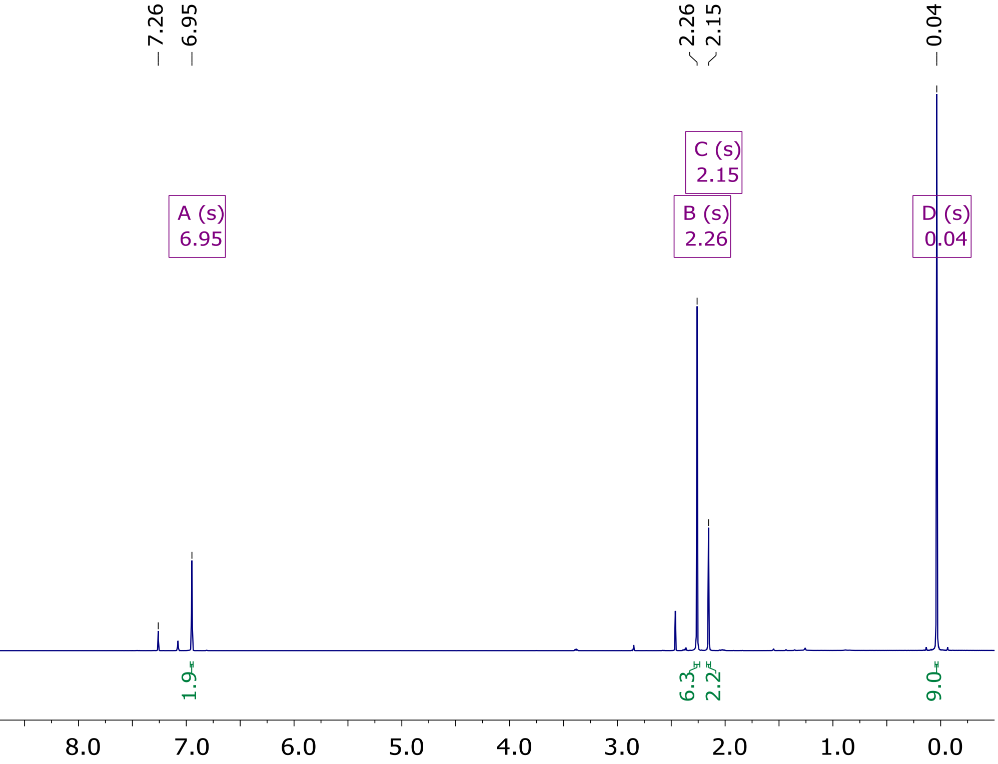

13C NMR
(150.85 MHz, CDCl3)

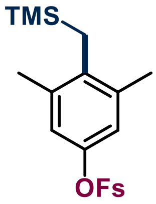

11

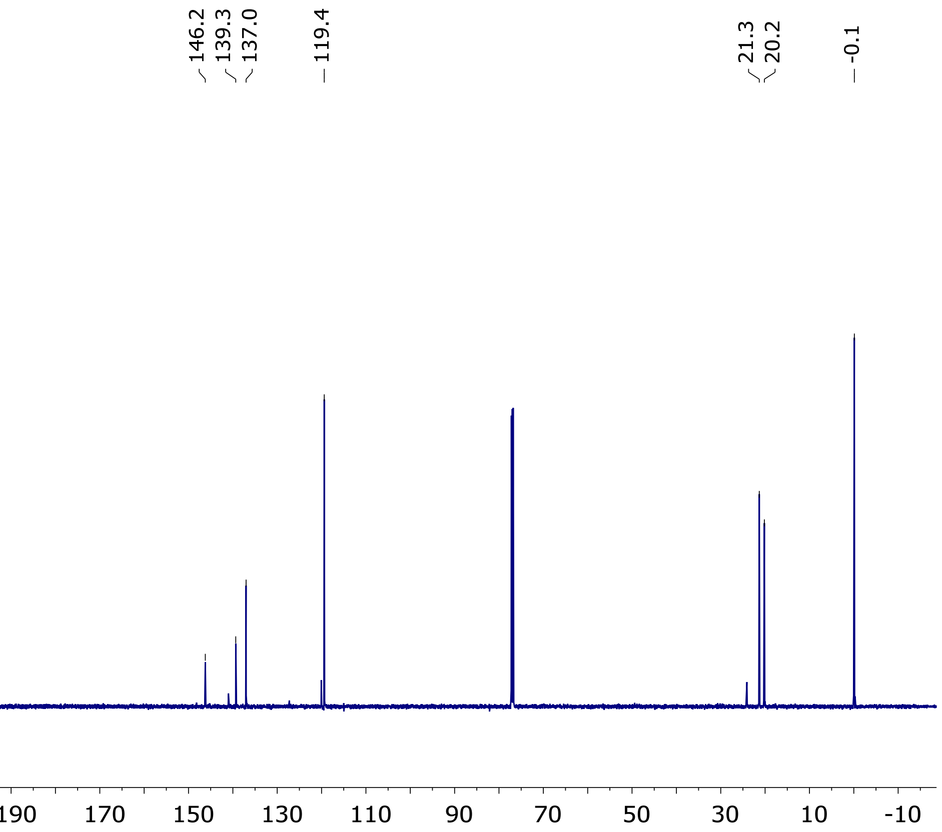

19F NMR
(564.40 MHz, CDCl3)

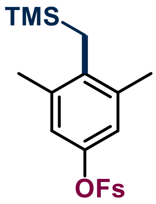

11

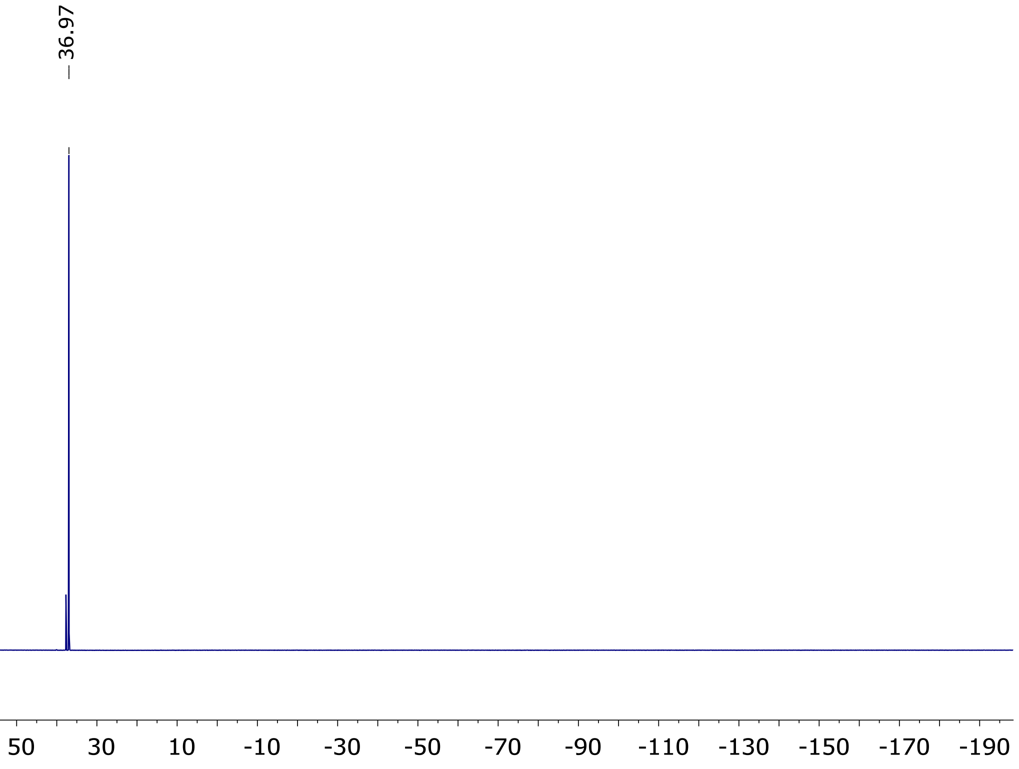

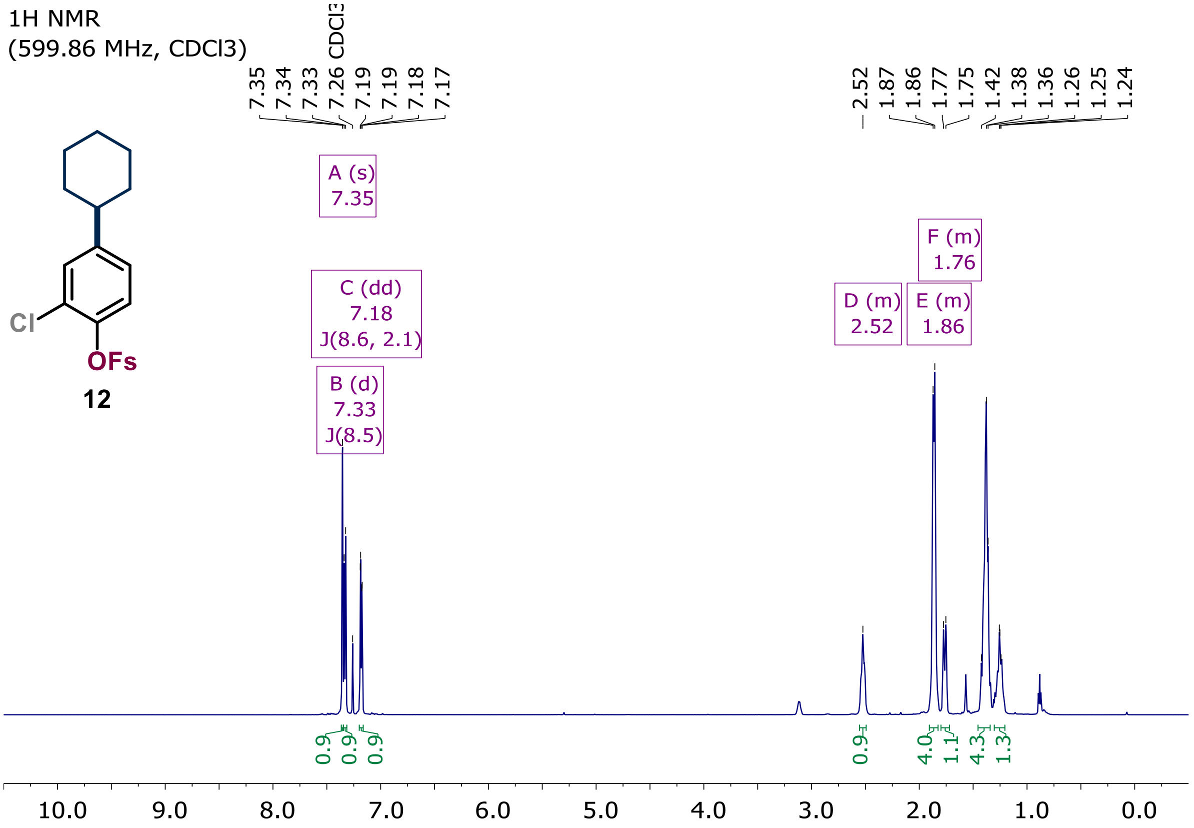
1H NMR
(599.86 MHz, CDCl3)
7.35 7.34 7.33 7.26 CDCl3 7.19 7.19 7.18 7.17
2.52 1.87 1.86 1.77 1.75 1.42 1.38 1.36 1.26 1.25 1.24
A (s) 7.35
C (dd) 7.18 J(8.6, 2.1)
B (d) 7.33 J(8.5)
F (m) 1.76
D (m) 2.52
E (m) 1.86
Cl
OFs
12
0.9 0.9 0.9
0.9
4.0 1.1 4.3 1.3
10.0 9.0 8.0 7.0 6.0 5.0 4.0 3.0 2.0 1.0 0.0

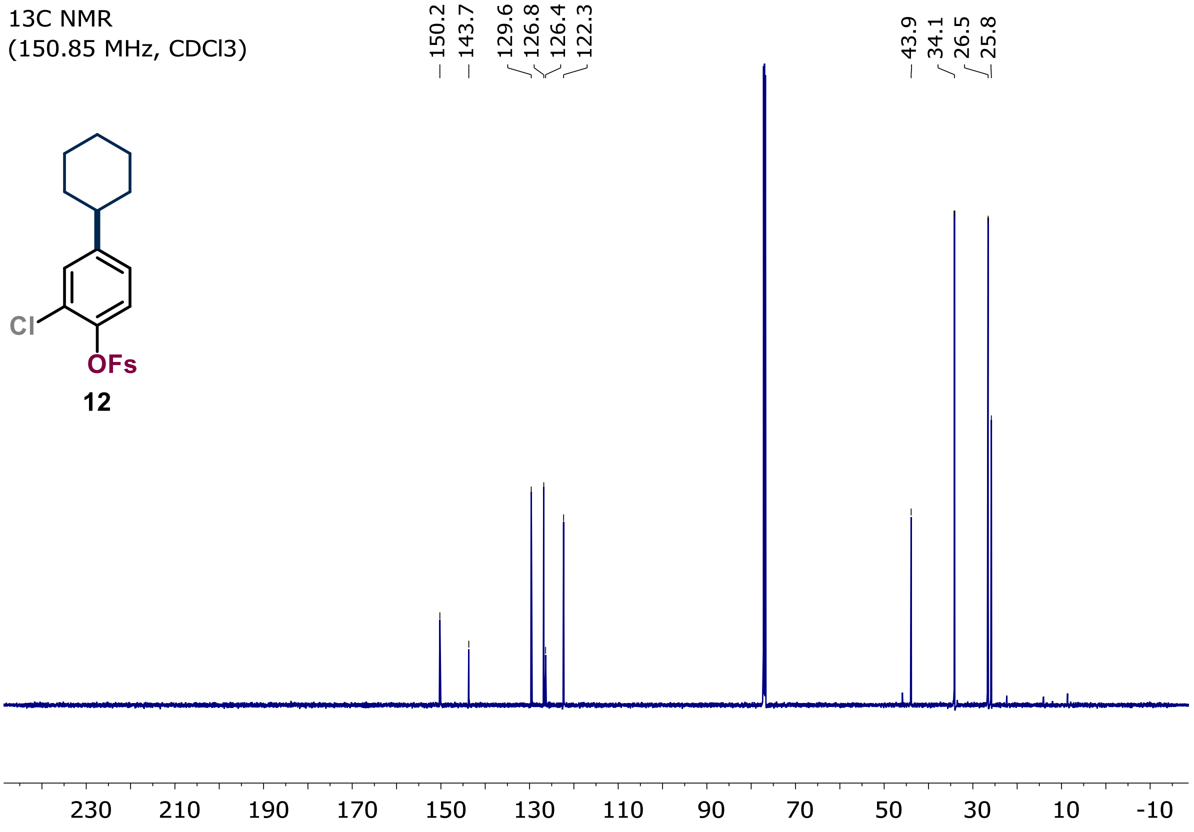
13C NMR
(150.85 MHz, CDCl3)
150.2 143.7 129.6 126.8 126.4 122.3
43.9 34.1 26.5 25.8
Cl
OFs
12
230 210 190 170 150 130 110 90 70 50 30 10 -10

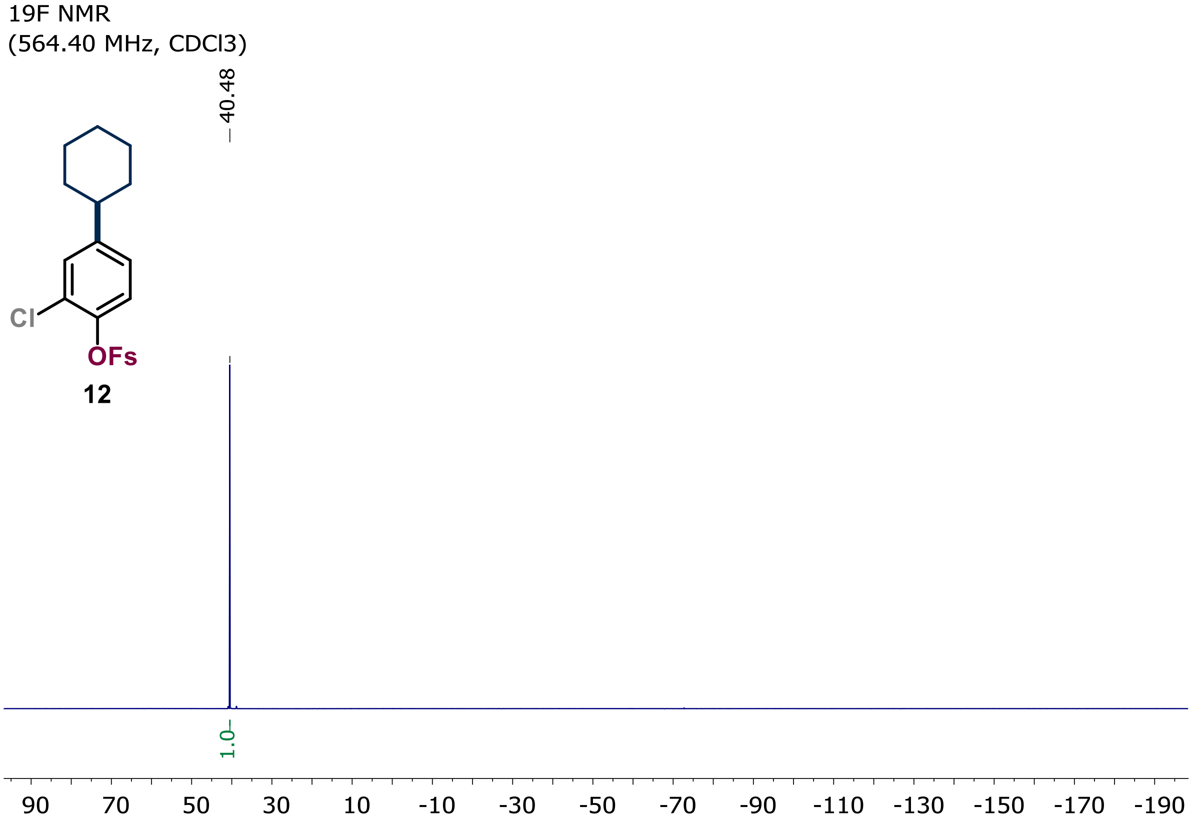
19F NMR
(564.40 MHz, CDCl3)
40.48
Cl
OFs
12
1.0
90 70 50 30 10 -10 -30 -50 -70 -90 -110 -130 -150 -170 -190

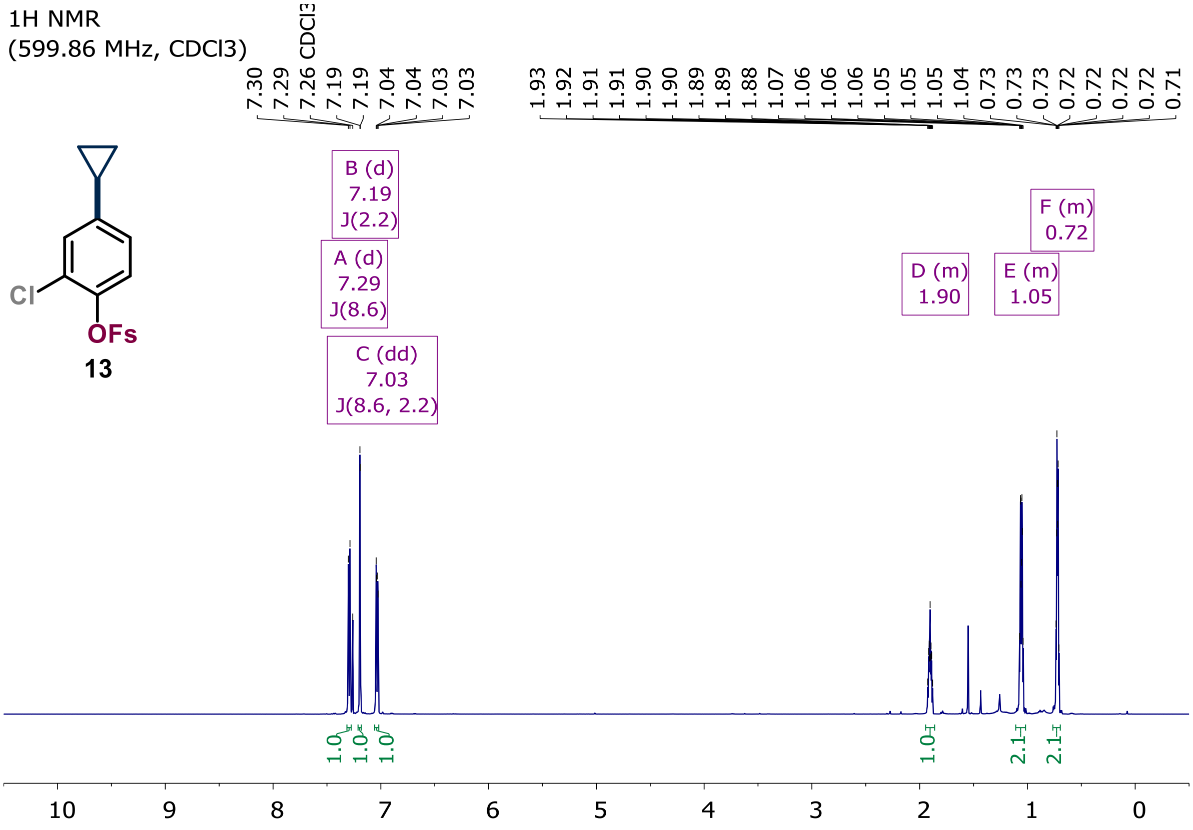
1H NMR
(599.86 MHz, CDCl3)
7.30 7.29 7.26 CDCl3 7.19 7.19 7.04 7.04 7.03 7.03
1.93 1.92 1.91 1.91 1.90 1.90 1.89 1.89 1.88 1.07 1.06 1.06 1.06 1.05 1.05 1.05 1.04 0.73 0.73 0.73 0.72 0.72 0.72 0.72 0.71
B (d) 7.19 J(2.2)
A (d) 7.29 J(8.6)
C (dd) 7.03 J(8.6, 2.2)
D (m) 1.90
E (m) 1.05
F (m) 0.72
Cl
OFs
13
1.0 1.0 1.0 1.0 2.1 2.1
10 9 8 7 6 5 4 3 2 1 0

13C NMR
(150.85 MHz, CDCl3)

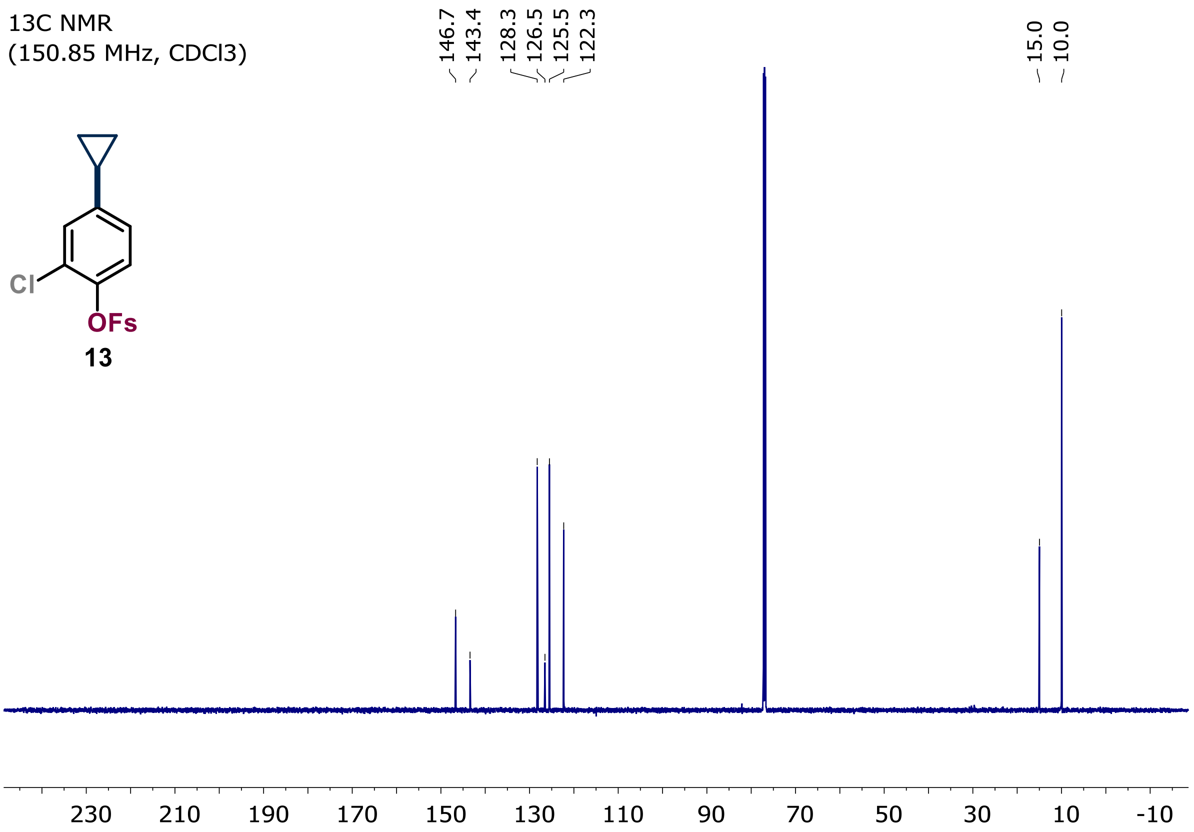

19F NMR
(564.40 MHz, CDCl3)

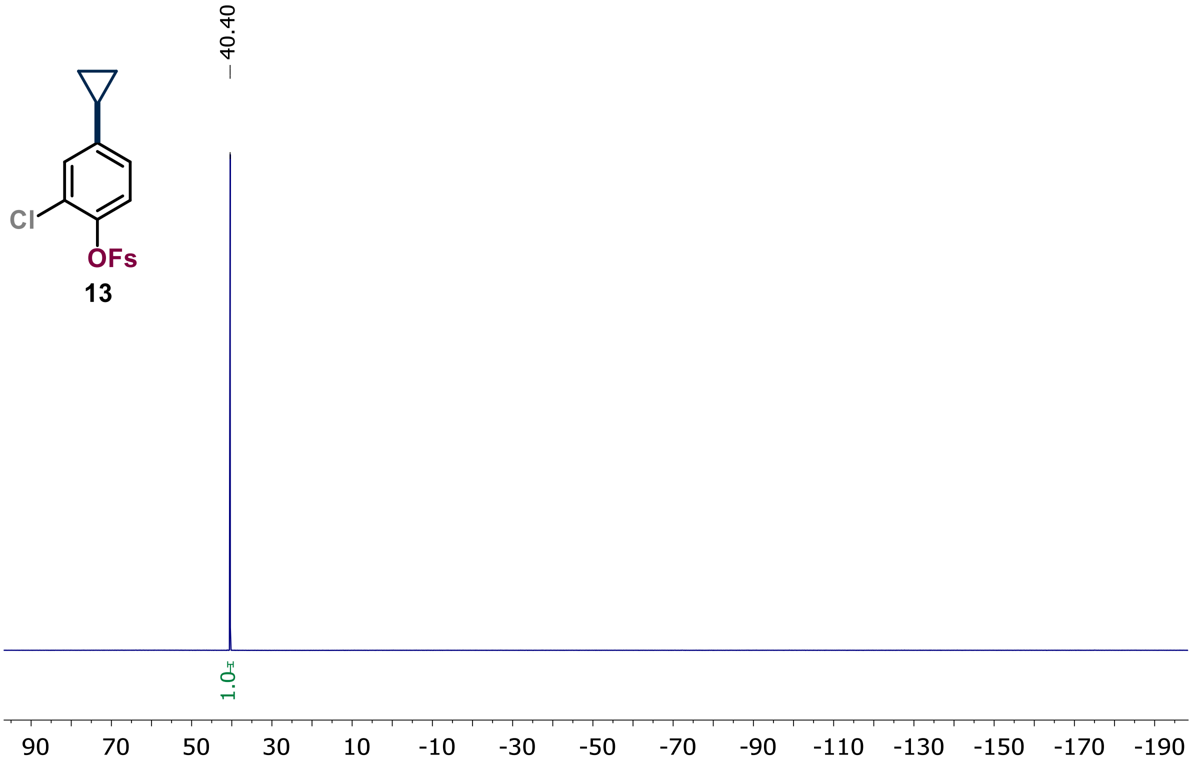

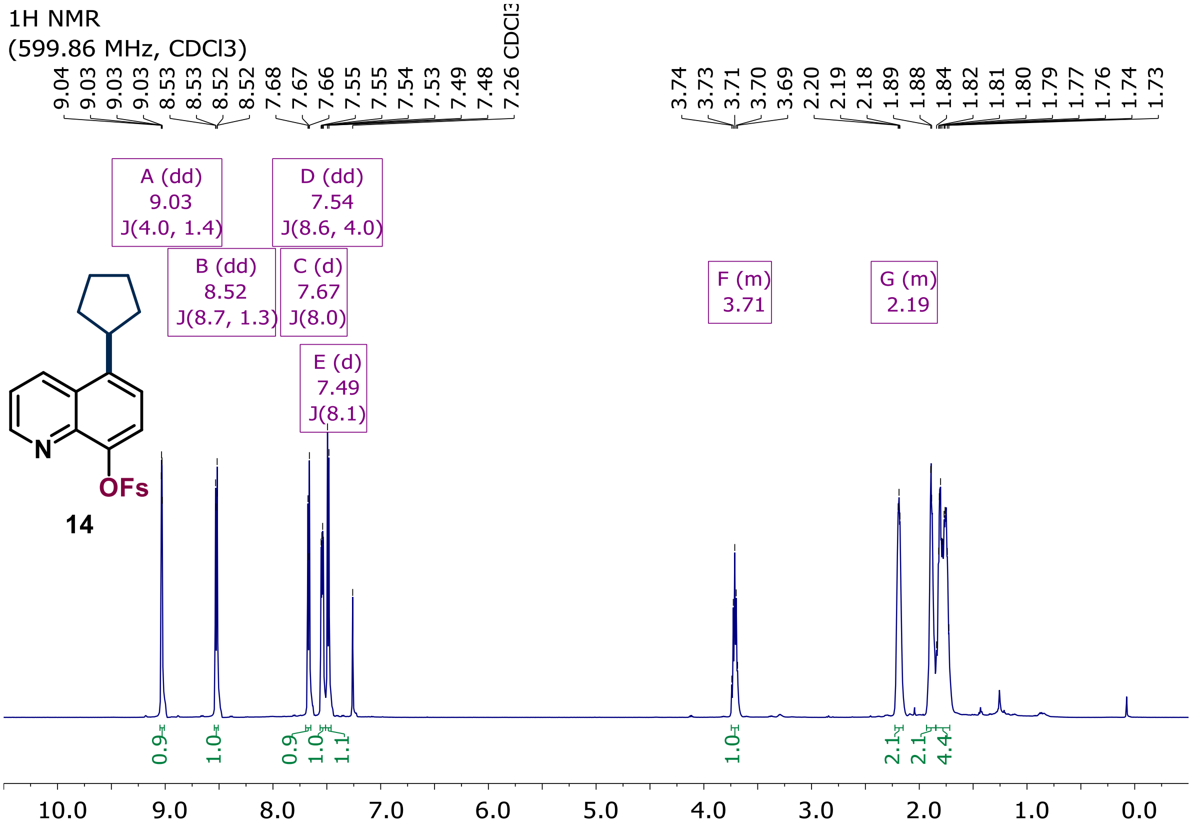
1H NMR
(599.86 MHz, CDCl3)
9.04 9.03 9.03 9.03 8.53 8.53 8.52 8.52 7.68 7.67 7.66 7.55 7.55 7.54 7.53 7.49 7.48 7.26 CDCl3
3.74 3.73 3.71 3.70 3.69 2.20 2.19 2.18 1.89 1.88 1.84 1.82 1.81 1.80 1.79 1.77 1.76 1.74 1.73
A (dd) 9.03 J(4.0, 1.4)
B (dd) 8.52 J(8.7, 1.3)
D (dd) 7.54 J(8.6, 4.0)
C (d) 7.67 J(8.0)
E (d) 7.49 J(8.1)
F (m) 3.71
G (m) 2.19
N
OFs
14
0.9 1.0 0.9 1.0 1.1 1.0 2.1 2.1 4.4
10.0 9.0 8.0 7.0 6.0 5.0 4.0 3.0 2.0 1.0 0.0

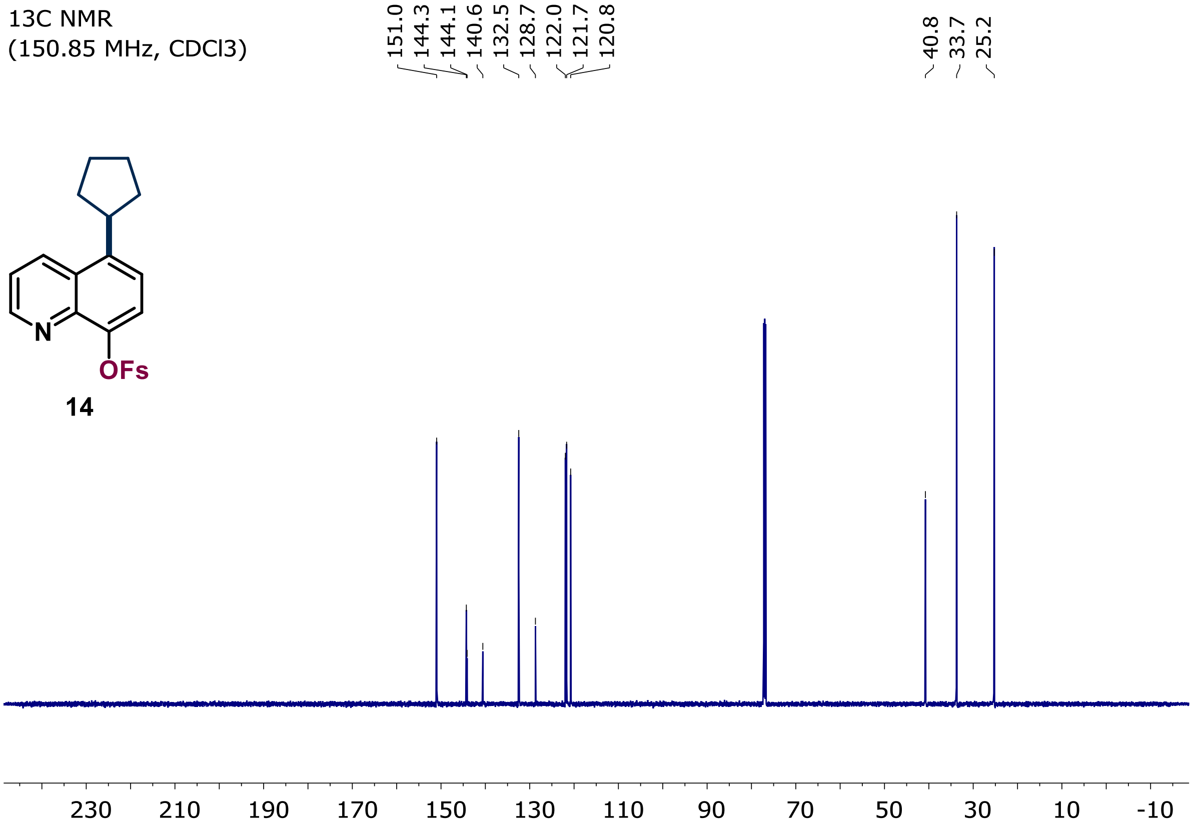
13C NMR
(150.85 MHz, CDCl3)
151.0 144.3 144.1 140.6 132.5 128.7 122.0 121.7 120.8
40.8 33.7 25.2
N
OFs
14
230 210 190 170 150 130 110 90 70 50 30 10 -10

19F NMR
(564.40 MHz, CDCl3)

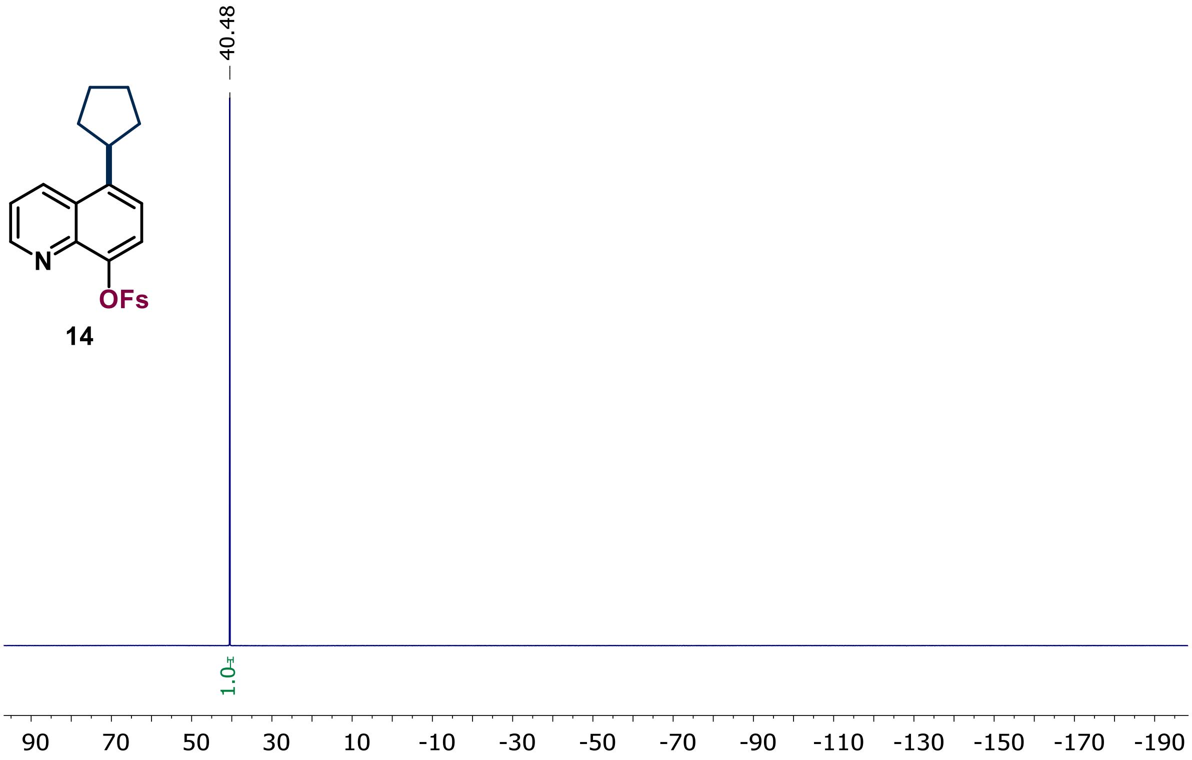

1H NMR
(399.97 MHz, CDCl3)

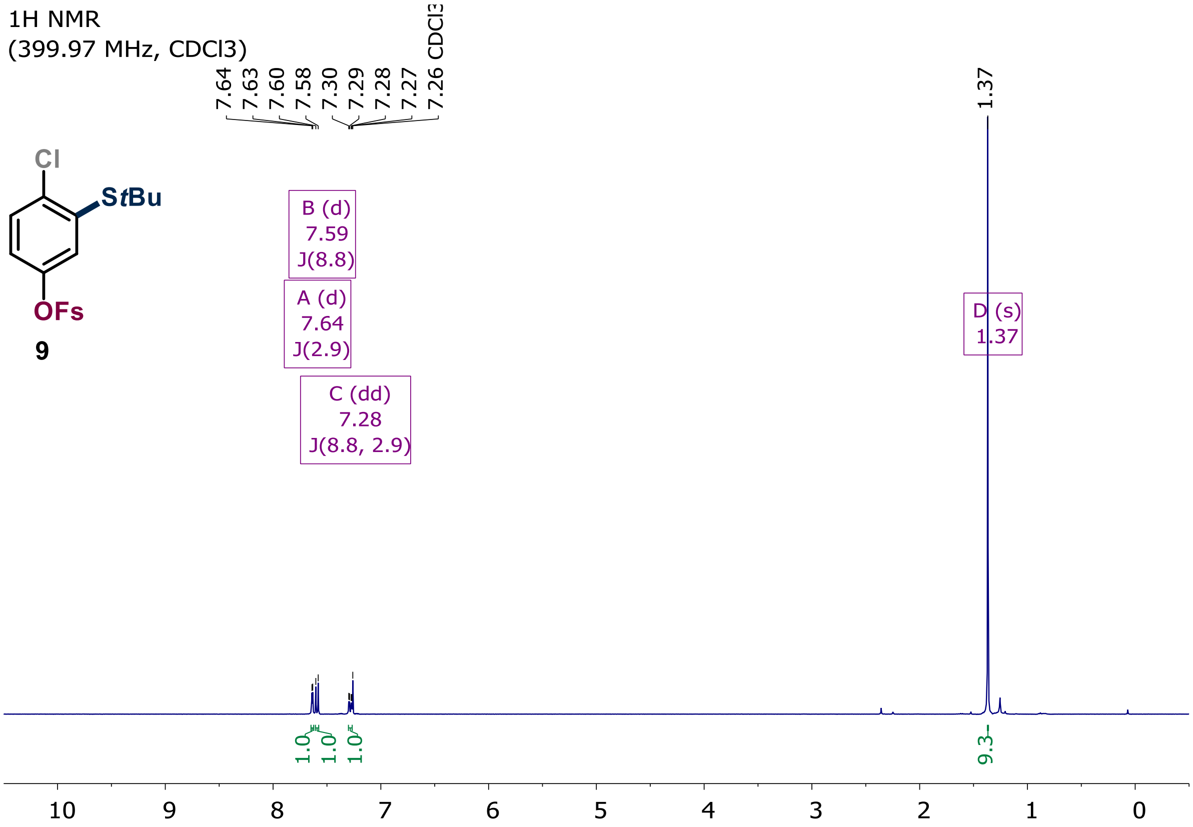

13C NMR
(100.58 MHz, CDCl3)

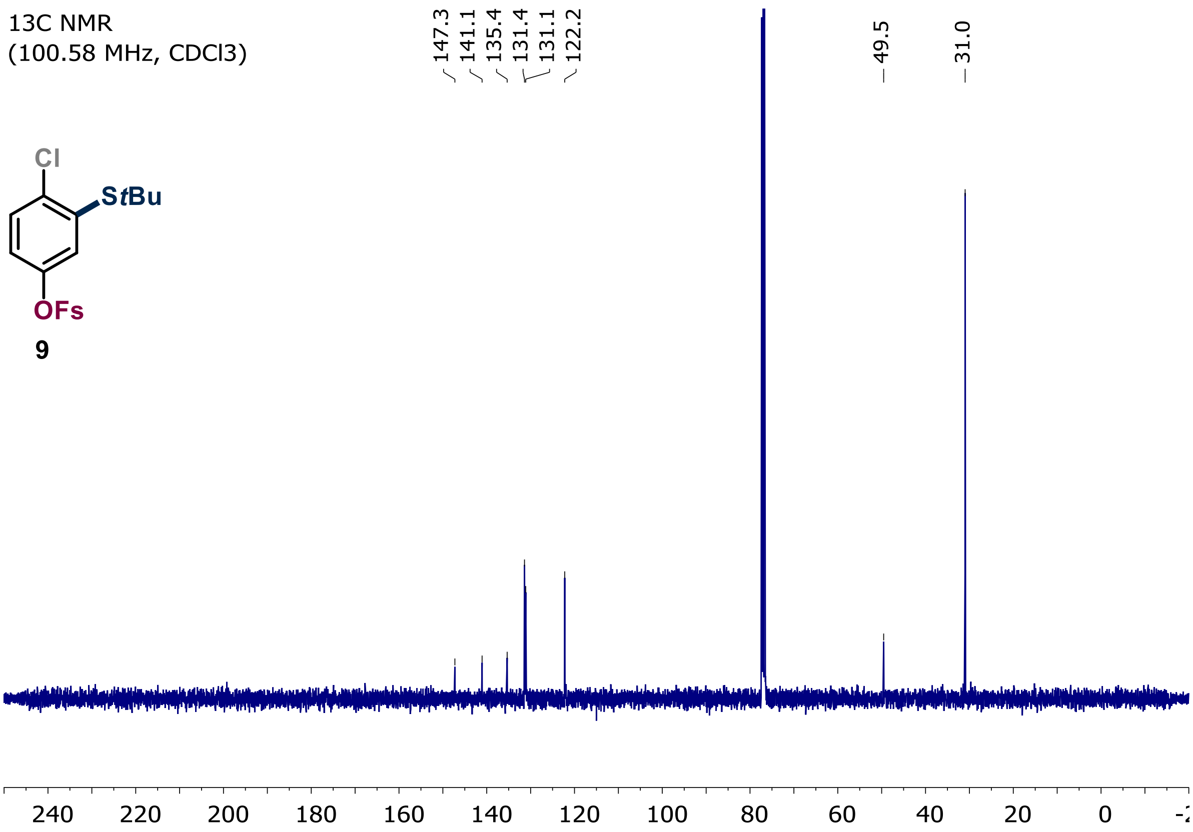

19F NMR
(376.33 MHz, CDCl3)

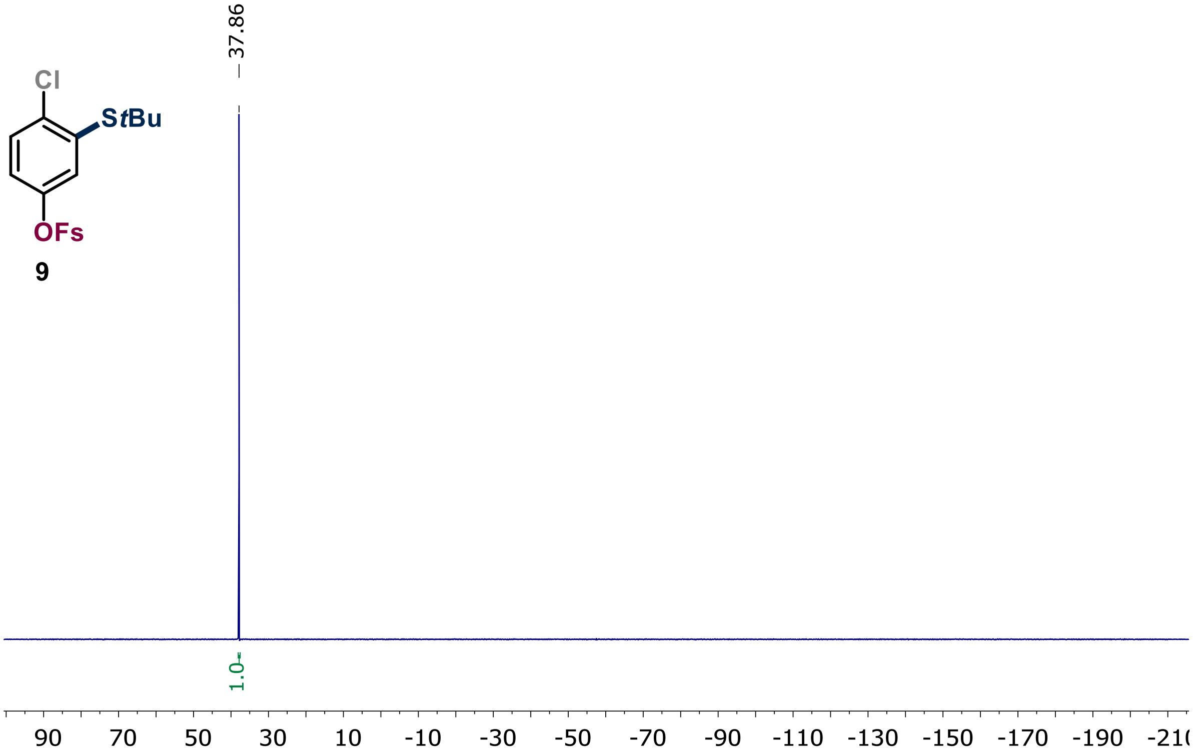

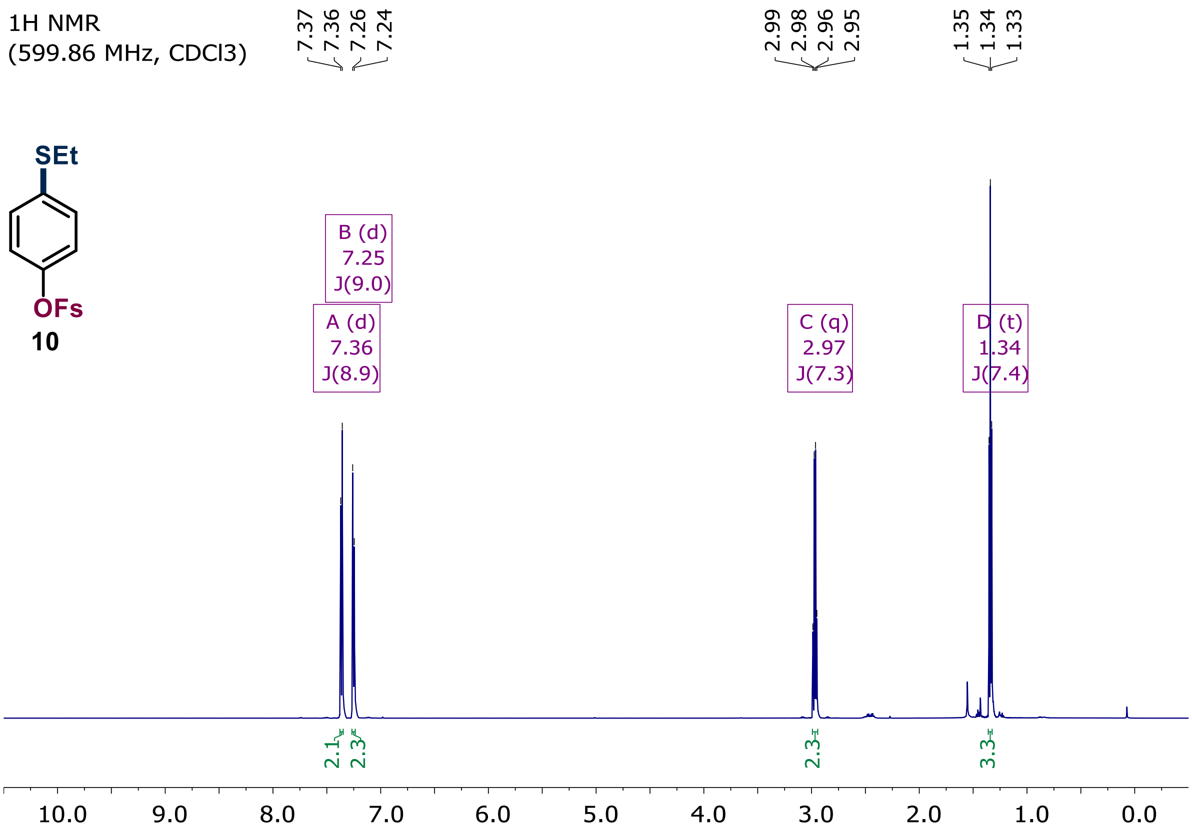
1H NMR
(599.86 MHz, CDCl3)
7.37
7.36
7.26
7.24
2.99
2.98
2.96
2.95
1.35
1.34
1.33
SEt
OFs
10
B (d) 7.25 J(9.0)
A (d) 7.36 J(8.9)
C (q) 2.97 J(7.3)
D (t) 1.34 J(7.4)
2.1
2.3
2.3
3.3
10.0
9.0
8.0
7.0
6.0
5.0
4.0
3.0
2.0
1.0
0.0

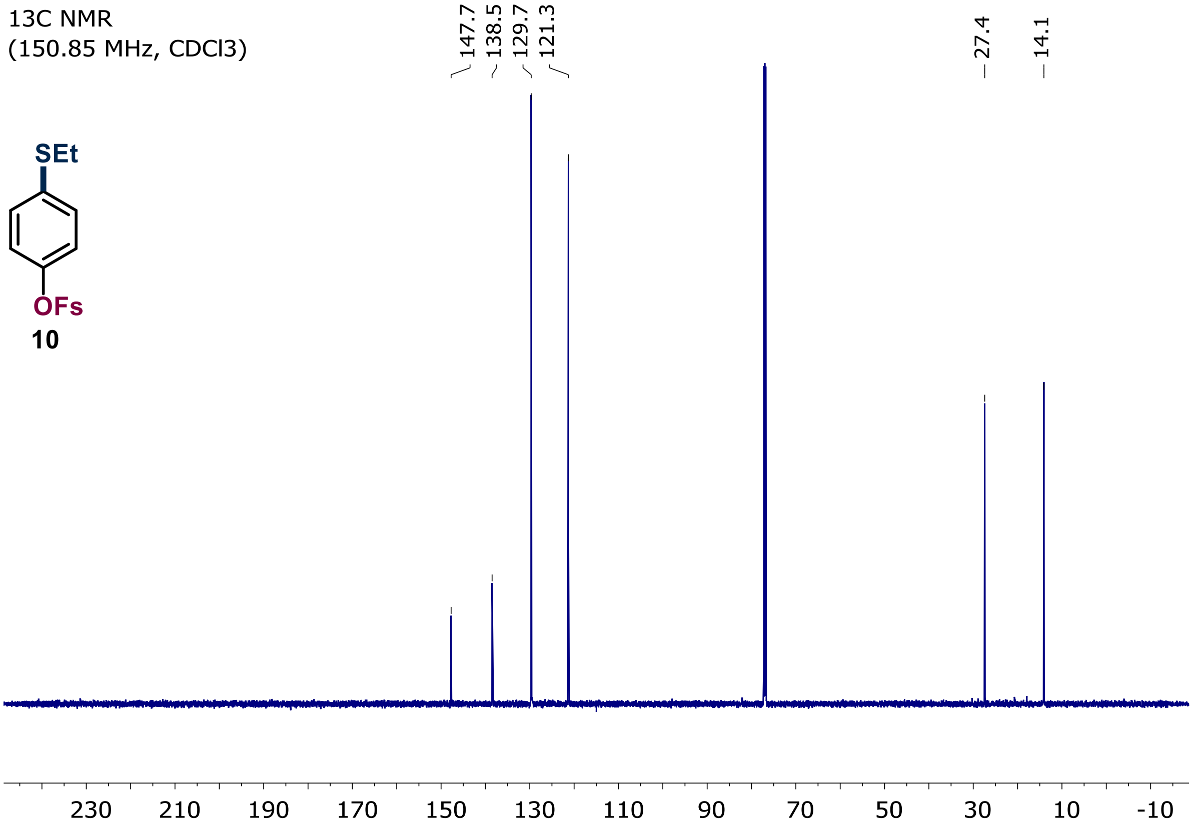
13C NMR
(150.85 MHz, CDCl3)
147.7
138.5
129.7
121.3
27.4
14.1
SEt
OFs
10
230
210
190
170
150
130
110
90
70
50
30
10
-10

19F NMR
(564.40 MHz, CDCl3)

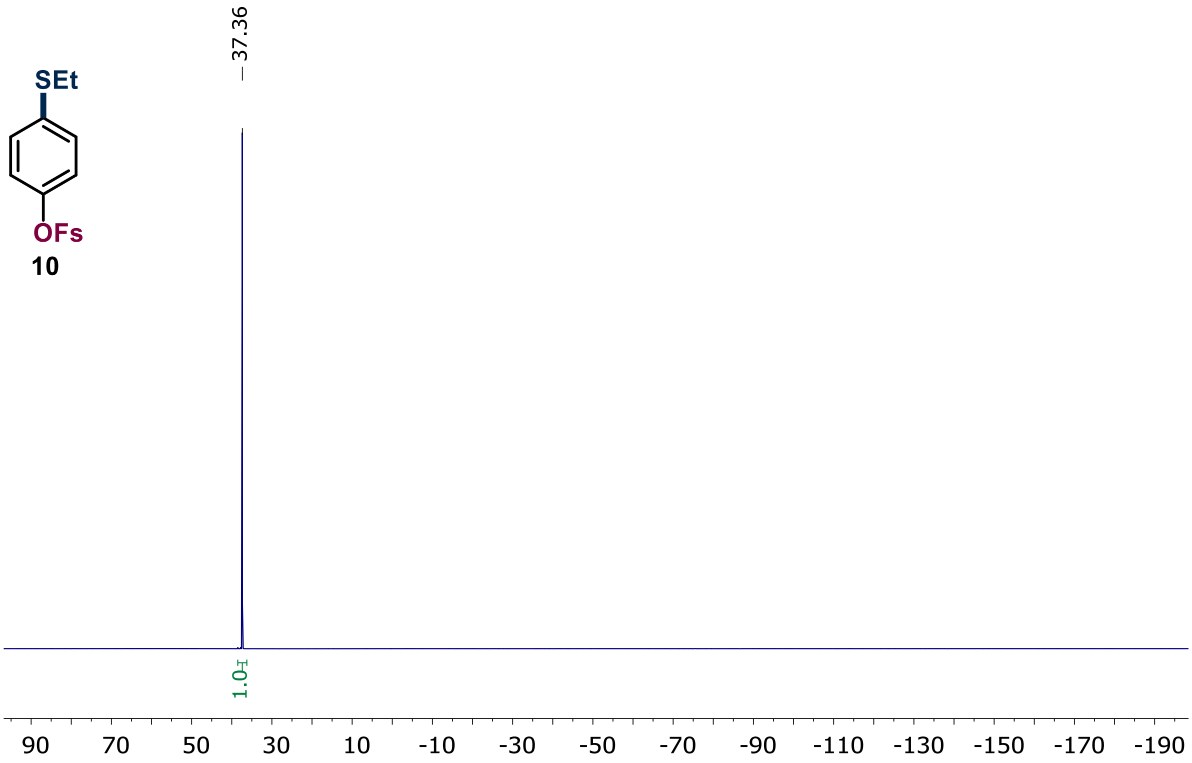

1H NMR
(599.86 MHz, CDCl3)

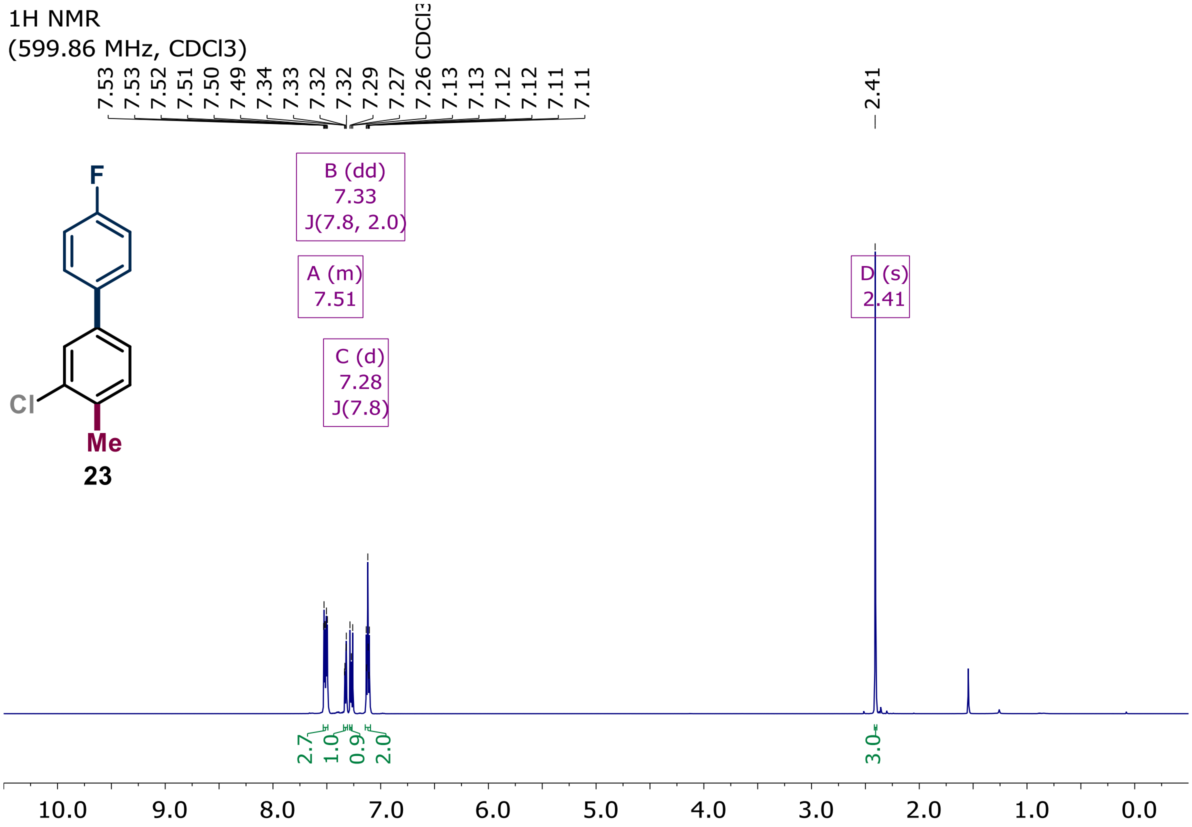

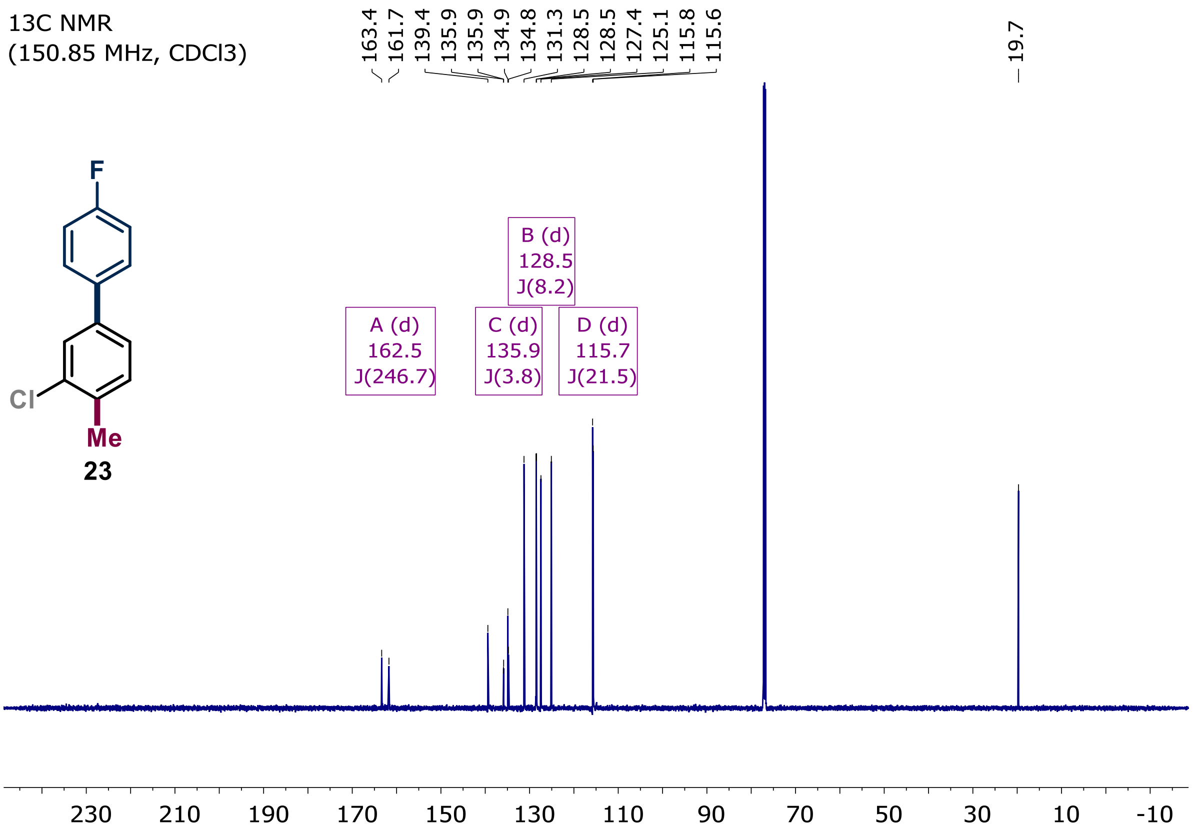
13C NMR
(150.85 MHz, CDCl3)
163.4
161.7
139.4
135.9
135.9
134.9
134.8
131.3
128.5
128.5
127.4
125.1
115.8
115.6
19.7
F
Cl
Me
23
B (d)
128.5
J(8.2)
A (d)
162.5
J(246.7)
C (d)
135.9
J(3.8)
D (d)
115.7
J(21.5)
230 210 190 170 150 130 110 90 70 50 30 10 -10

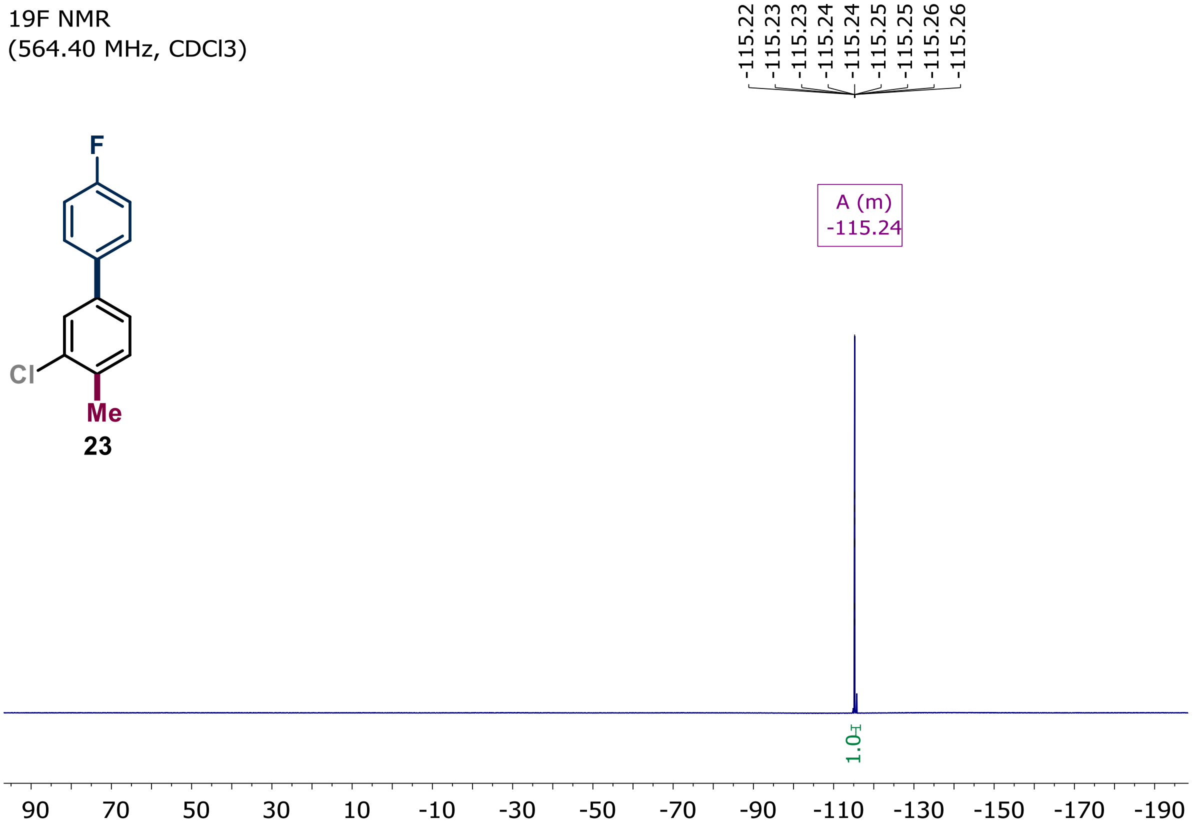
19F NMR
(564.40 MHz, CDCl3)
-115.22
-115.23
-115.23
-115.24
-115.24
-115.25
-115.25
-115.26
-115.26
F
Cl
Me
23
A (m)
-115.24
1.0
90 70 50 30 10 -10 -30 -50 -70 -90 -110 -130 -150 -170 -190

1H NMR
(599.86 MHz, CDCl3)

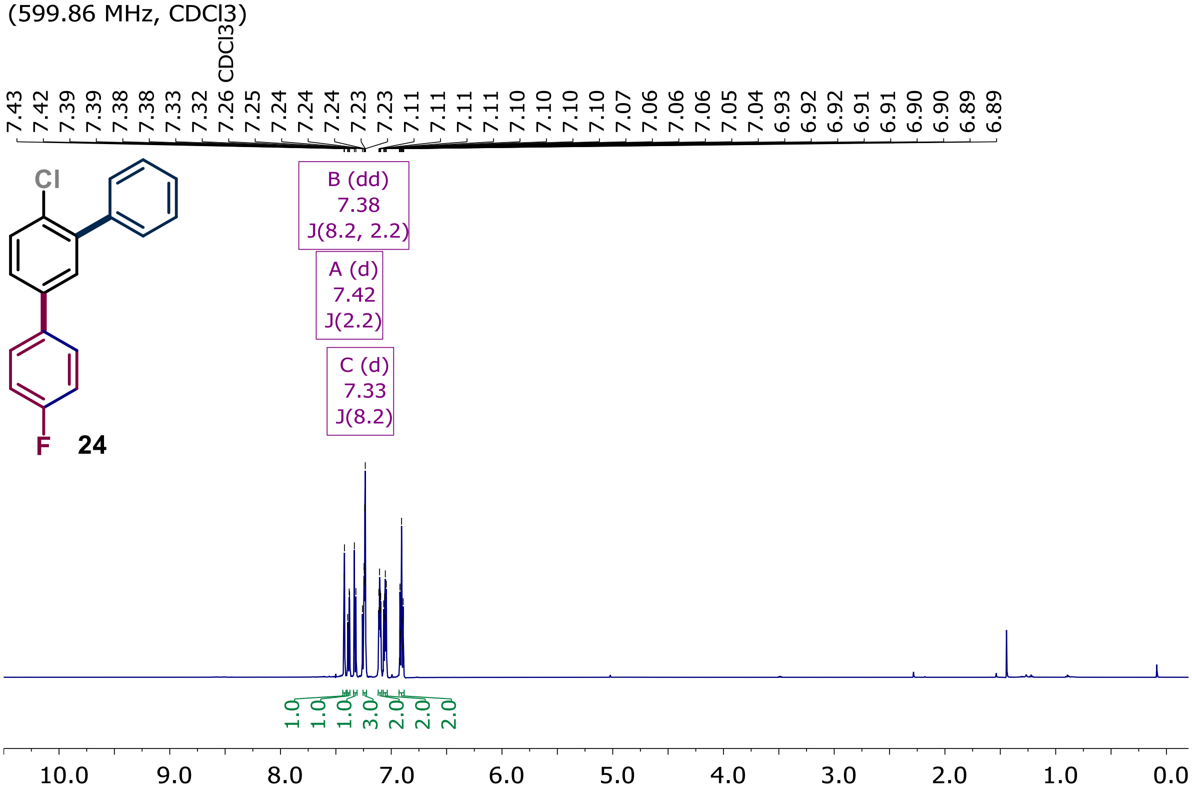

13C NMR
(150.85 MHz, CDCl3)

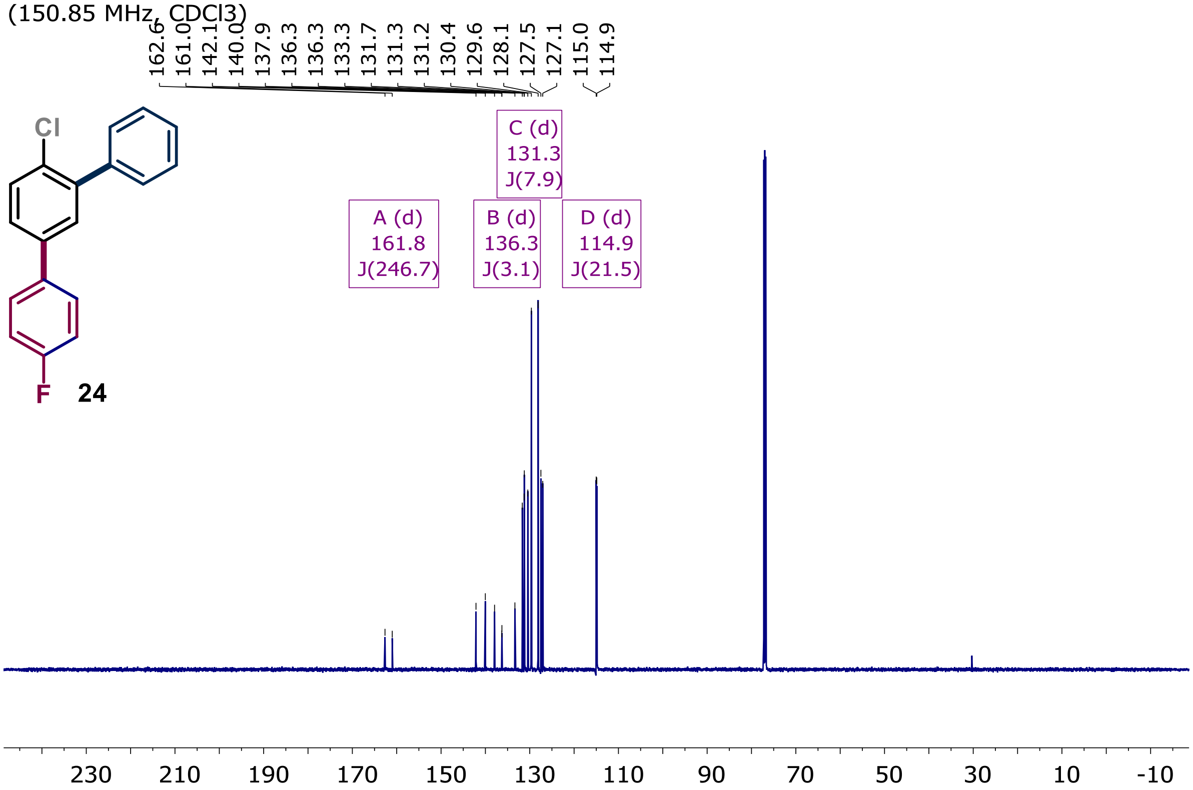

19F NMR
(564.40 MHz, CDCl3)

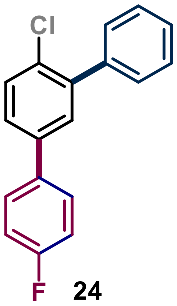

24

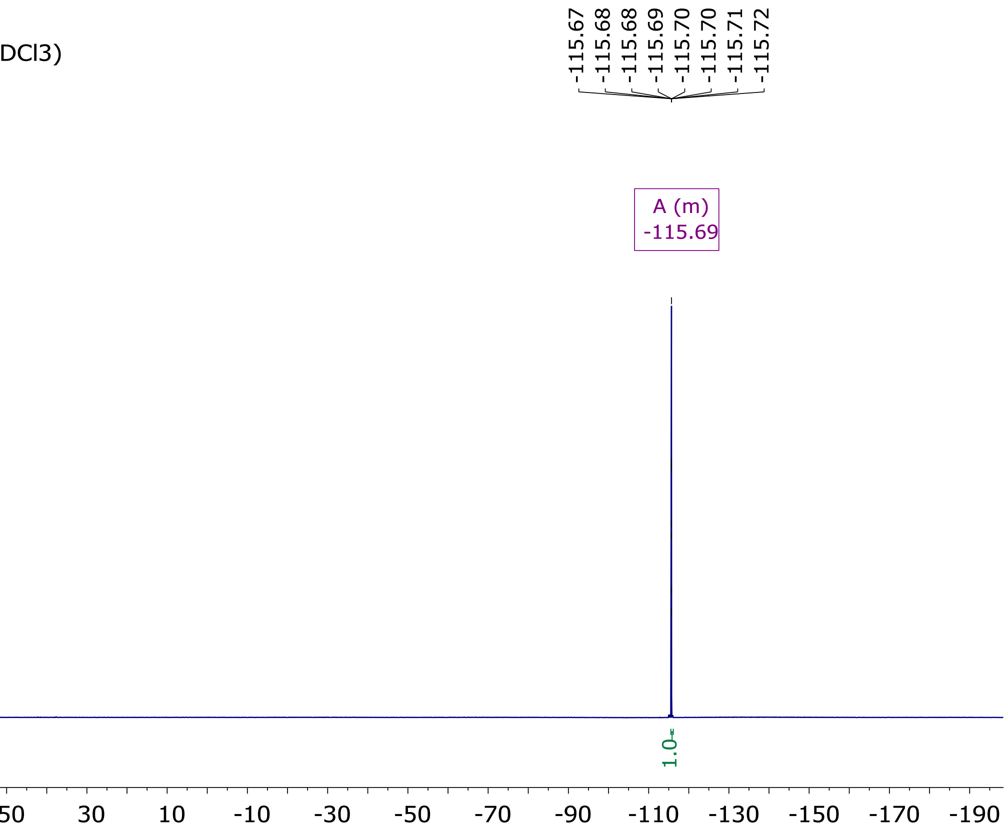

1H NMR
(599.86 MHz, CDCl3)

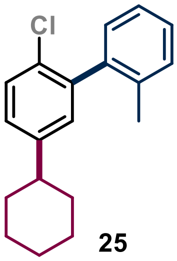

25

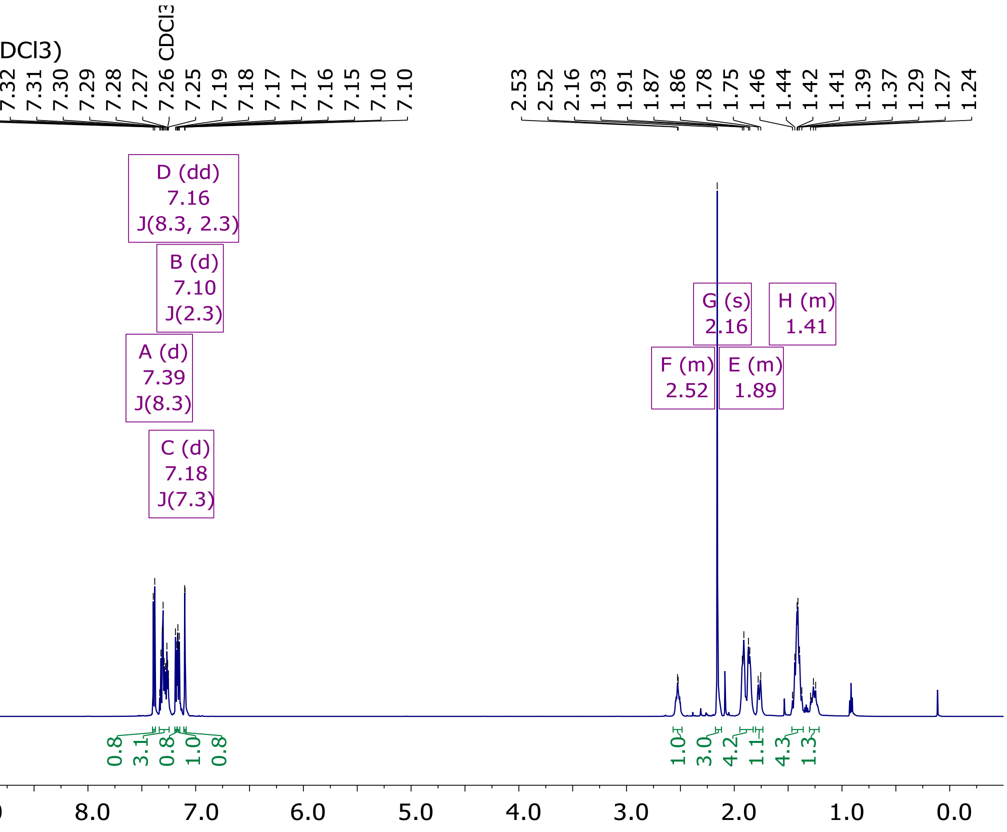

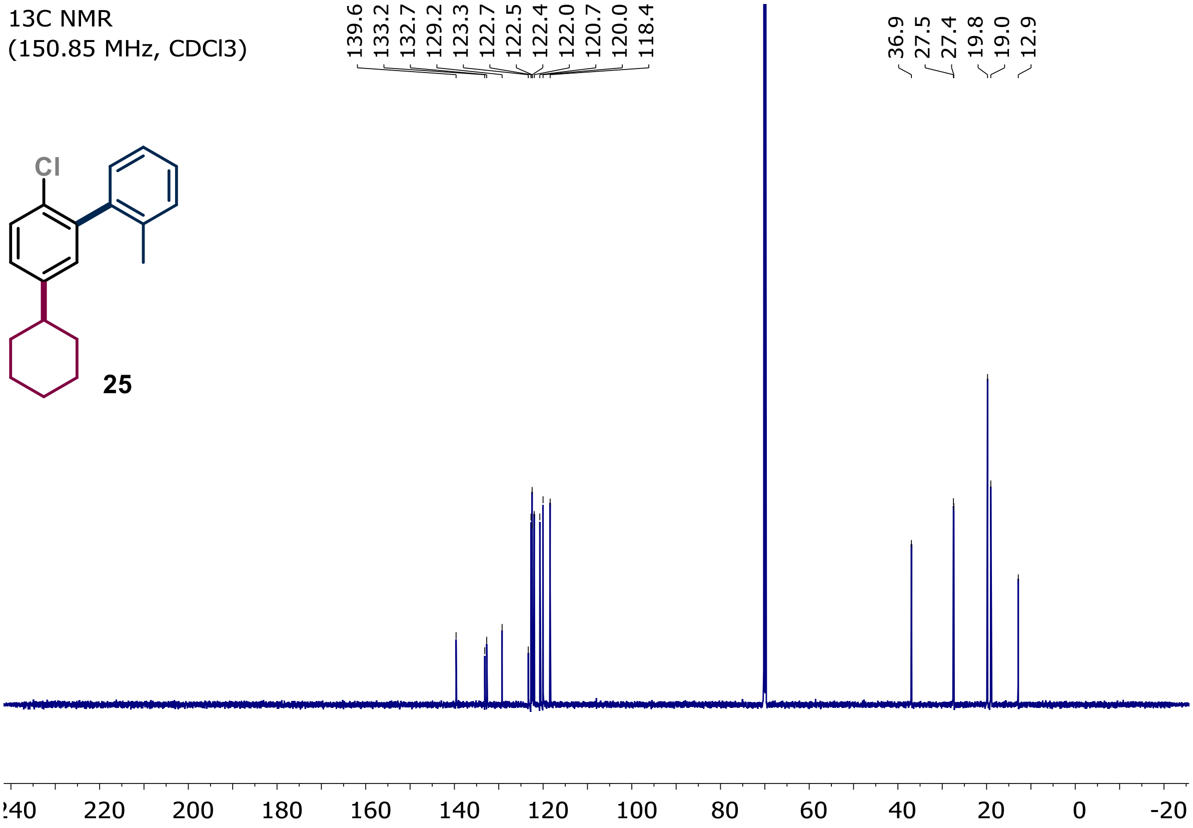
13C NMR
(150.85 MHz, CDCl3)
139.6
133.2
132.7
129.2
123.3
122.7
122.5
122.4
122.0
120.7
120.0
118.4
36.9
27.5
27.4
19.8
19.0
12.9
Cl
25
240
220
200
180
160
140
120
100
80
60
40
20
0
-20

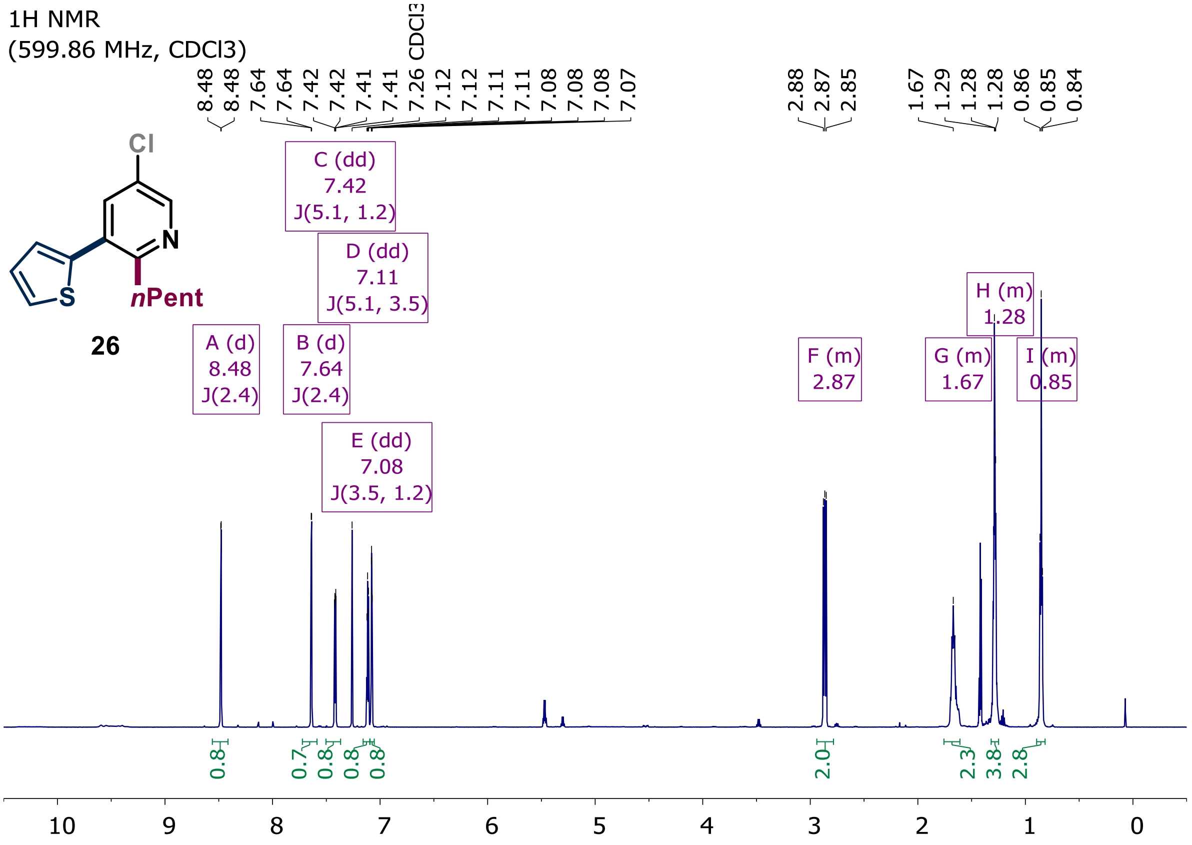
1H NMR
(599.86 MHz, CDCl3)
8.48
8.48
7.64
7.64
7.42
7.42
7.41
7.41
7.26 CDCl3
7.12
7.12
7.11
7.11
7.08
7.08
7.08
7.07
2.88
2.87
2.85
1.67
1.29
1.28
1.28
0.86
0.85
0.84
Cl
N
S
nPent
26
A (d) 8.48 J(2.4)
B (d) 7.64 J(2.4)
C (dd) 7.42 J(5.1, 1.2)
D (dd) 7.11 J(5.1, 3.5)
E (dd) 7.08 J(3.5, 1.2)
F (m) 2.87
G (m) 1.67
H (m) 1.28
I (m) 0.85
0.8
0.7
0.8
0.8
0.8
2.0
2.3
3.8
2.8
10
9
8
7
6
5
4
3
2
1
0

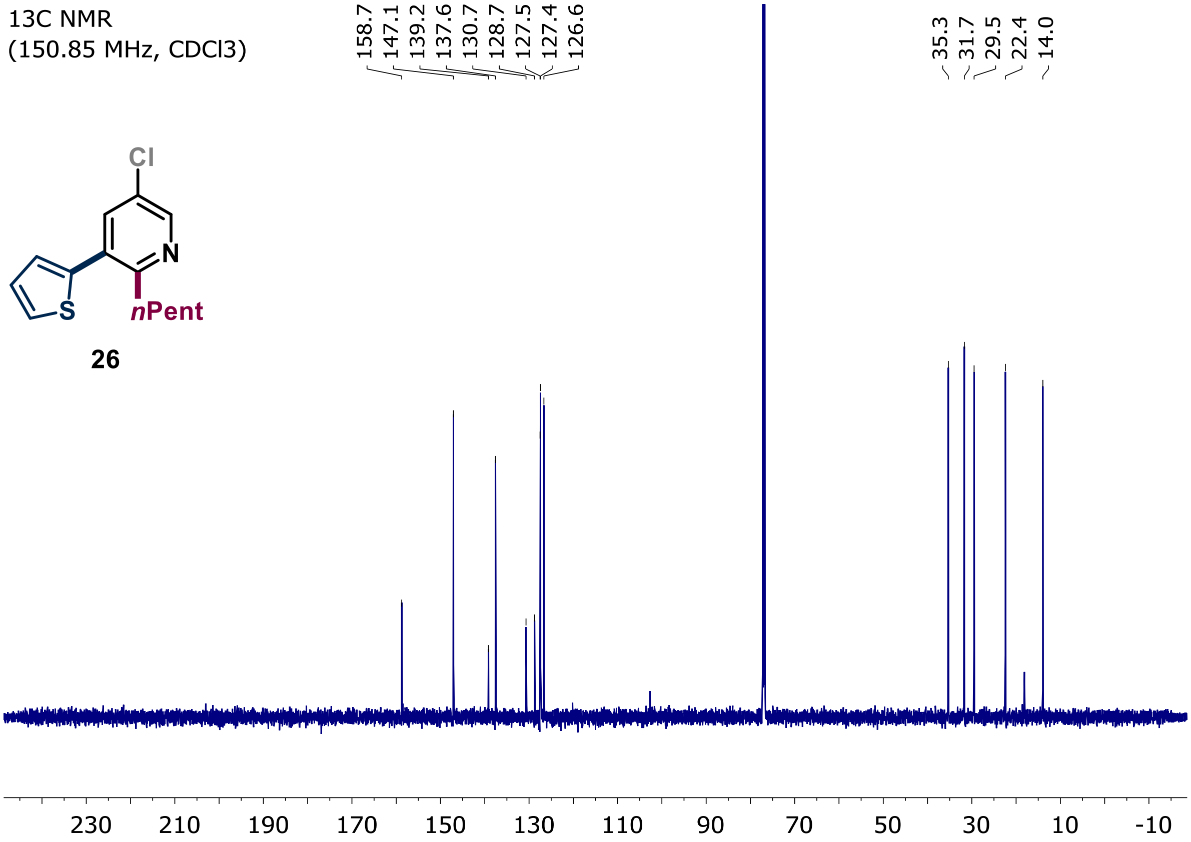
13C NMR
(150.85 MHz, CDCl3)
158.7
147.1
139.2
137.6
130.7
128.7
127.5
127.4
126.6
35.3
31.7
29.5
22.4
14.0
Cl
N
S
nPent
26
230
210
190
170
150
130
110
90
70
50
30
10
-10

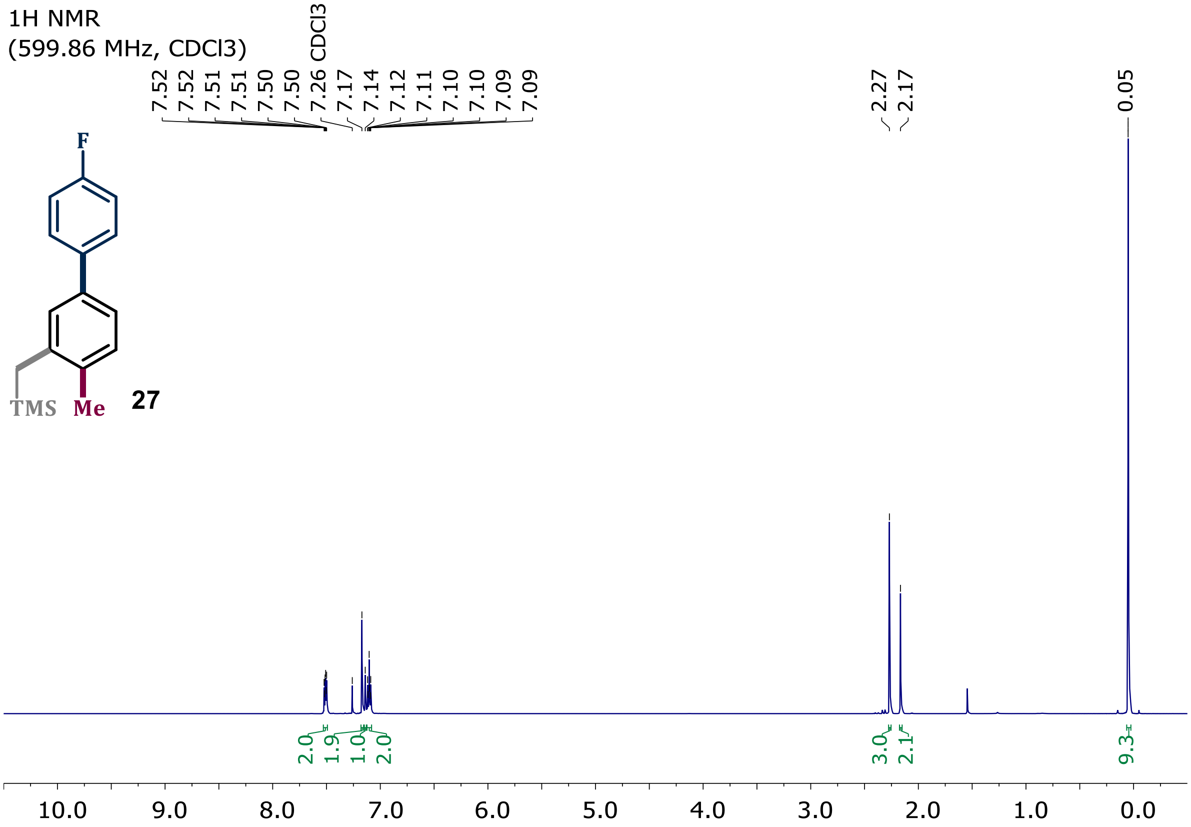
1H NMR
(599.86 MHz, CDCl3)
7.52
7.52
7.51
7.51
7.50
7.50
7.26 CDCl3
7.17
7.14
7.12
7.11
7.10
7.10
7.09
7.09
2.27
2.17
0.05
F
TMS
Me
27
2.0
1.9
1.0
2.0
3.0
2.1
9.3
10.0
9.0
8.0
7.0
6.0
5.0
4.0
3.0
2.0
1.0
0.0

13C NMR
(150.85 MHz, CDCl3)

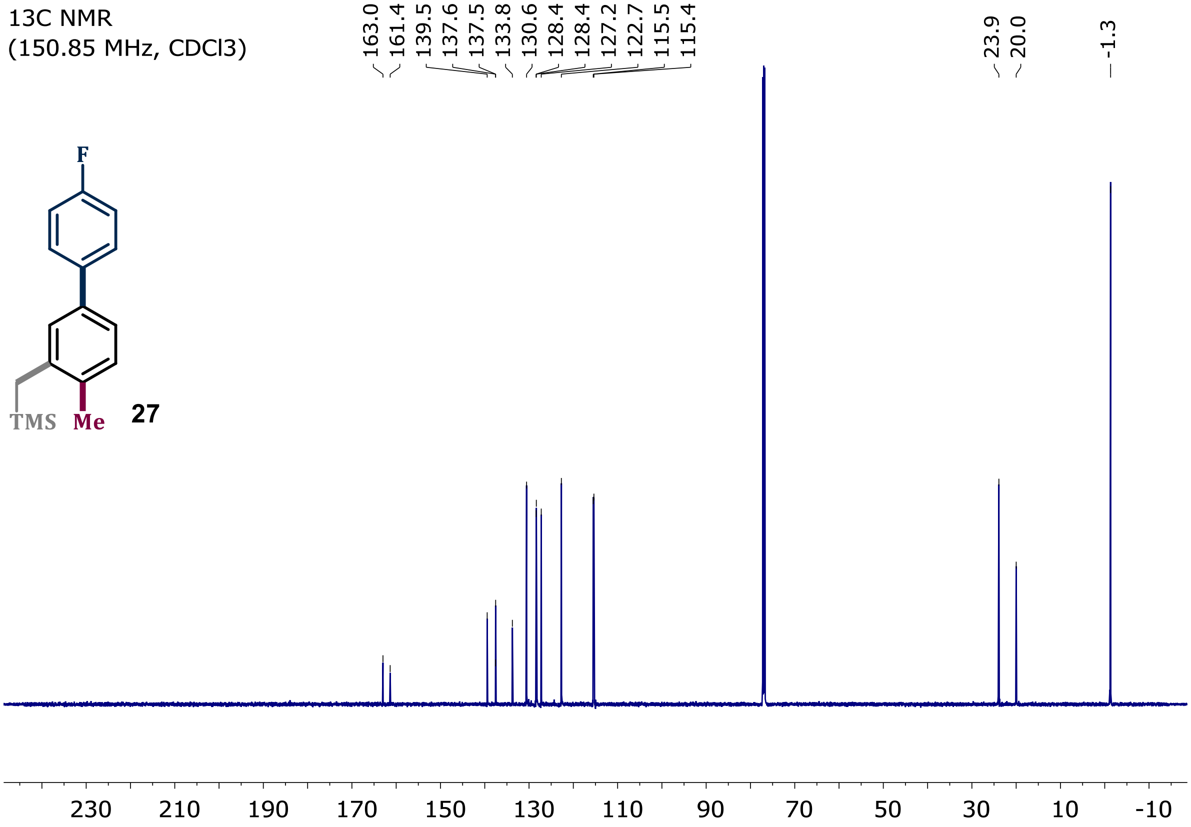

19F NMR
(564.40 MHz, CDCl3)

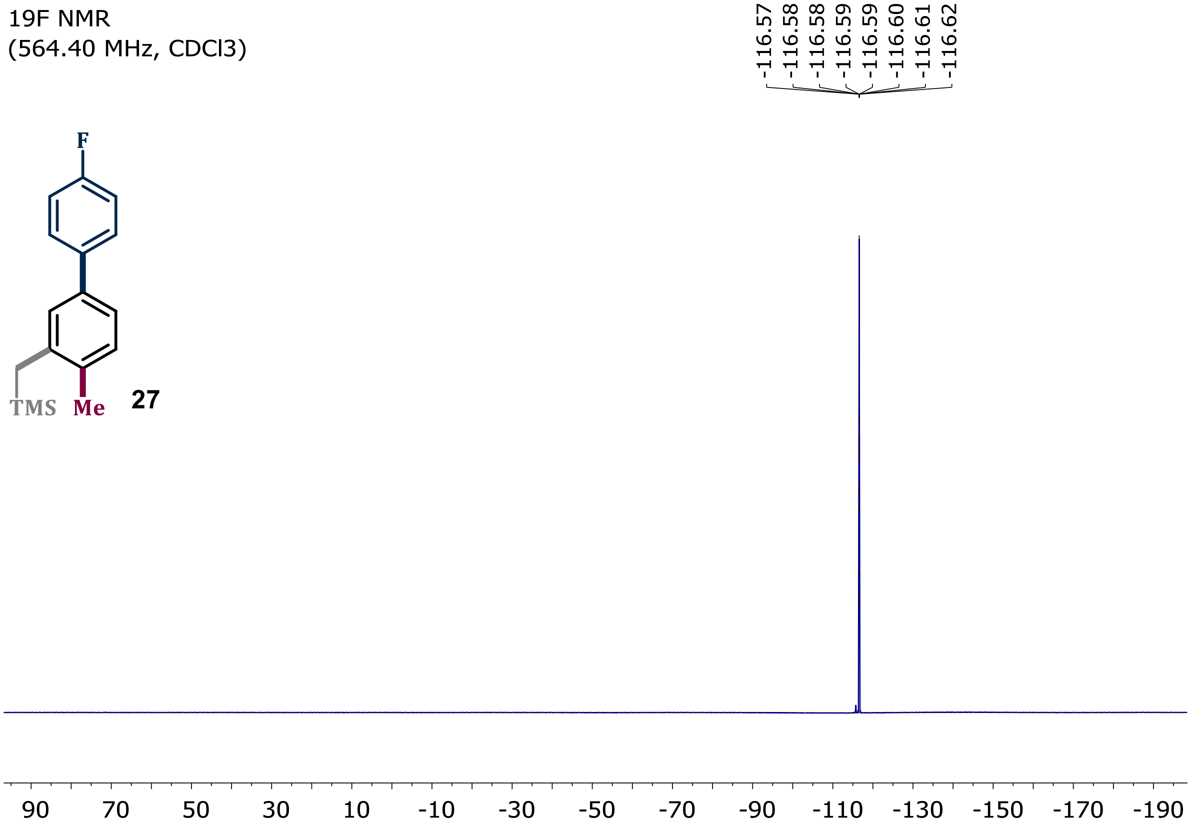

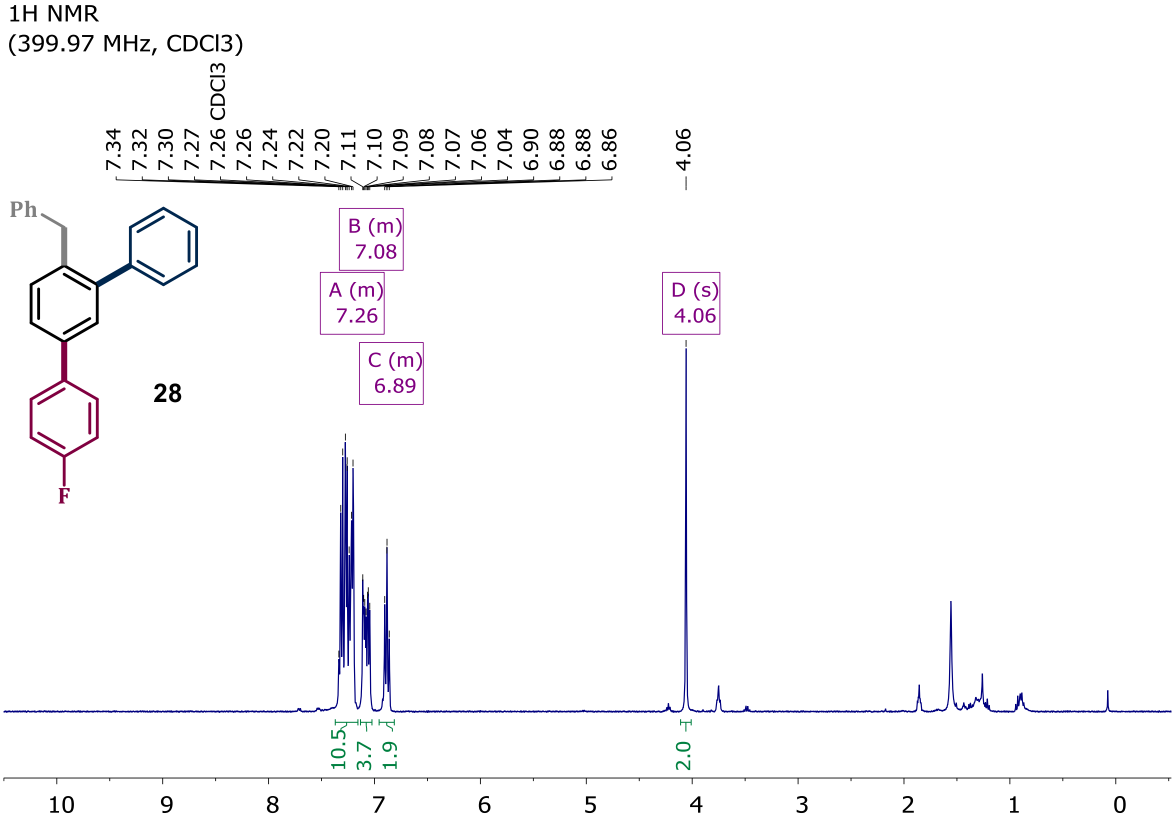
1H NMR
(399.97 MHz, CDCl3)
7.34 7.32 7.30 7.27 7.26 CDCl3 7.26 7.24 7.22 7.20 7.11 7.10 7.09 7.08 7.07 7.06 7.04 6.90 6.88 6.88 6.86
4.06
Ph
F
28
B (m) 7.08
A (m) 7.26
D (s) 4.06
C (m) 6.89
10.5 3.7 1.9
2.0
10 9 8 7 6 5 4 3 2 1 0

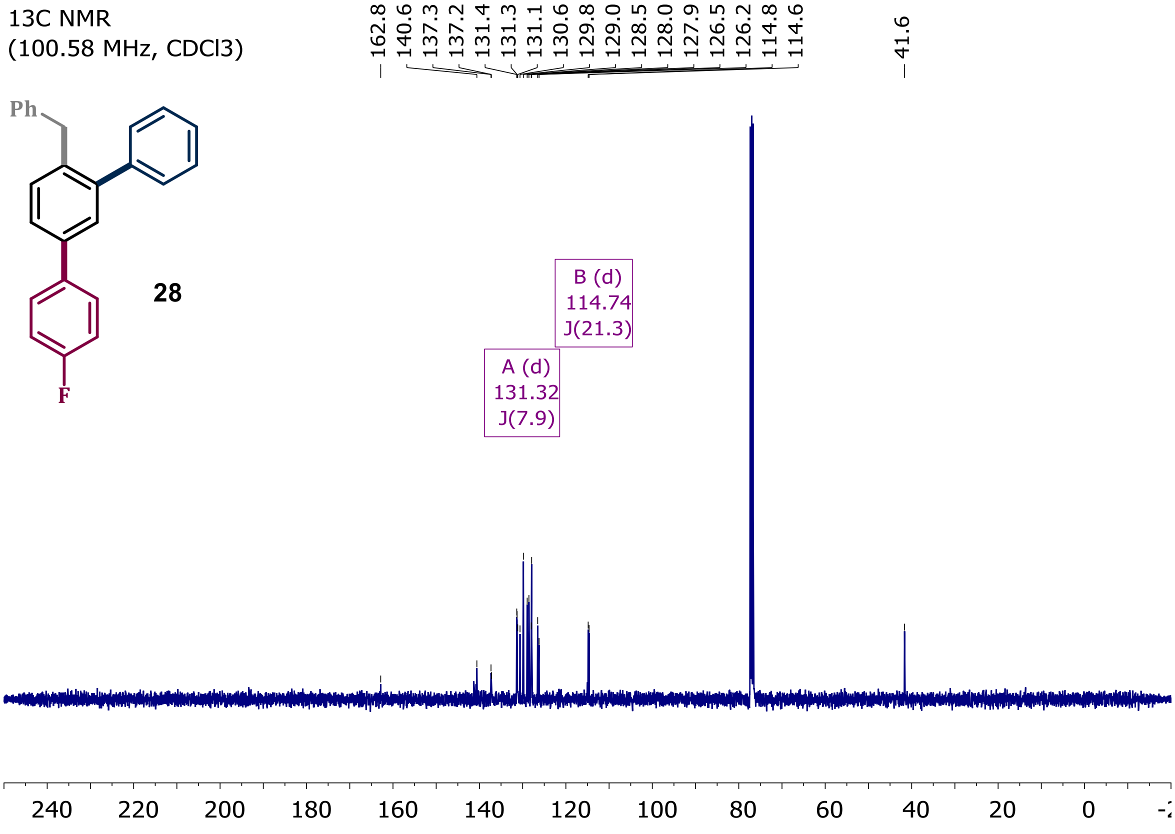
13C NMR
(100.58 MHz, CDCl3)
162.8 140.6 137.3 137.2 131.4 131.3 131.1 130.6 129.8 129.0 128.5 128.0 127.9 126.5 126.2 114.8 114.6
41.6
Ph
F
28
B (d) 114.74 J(21.3)
A (d) 131.32 J(7.9)
240 220 200 180 160 140 120 100 80 60 40 20 0

19F NMR
(376.33 MHz, CDCl3)

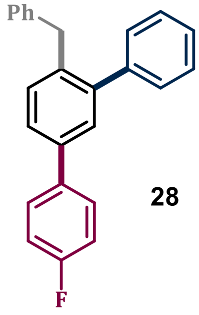

28

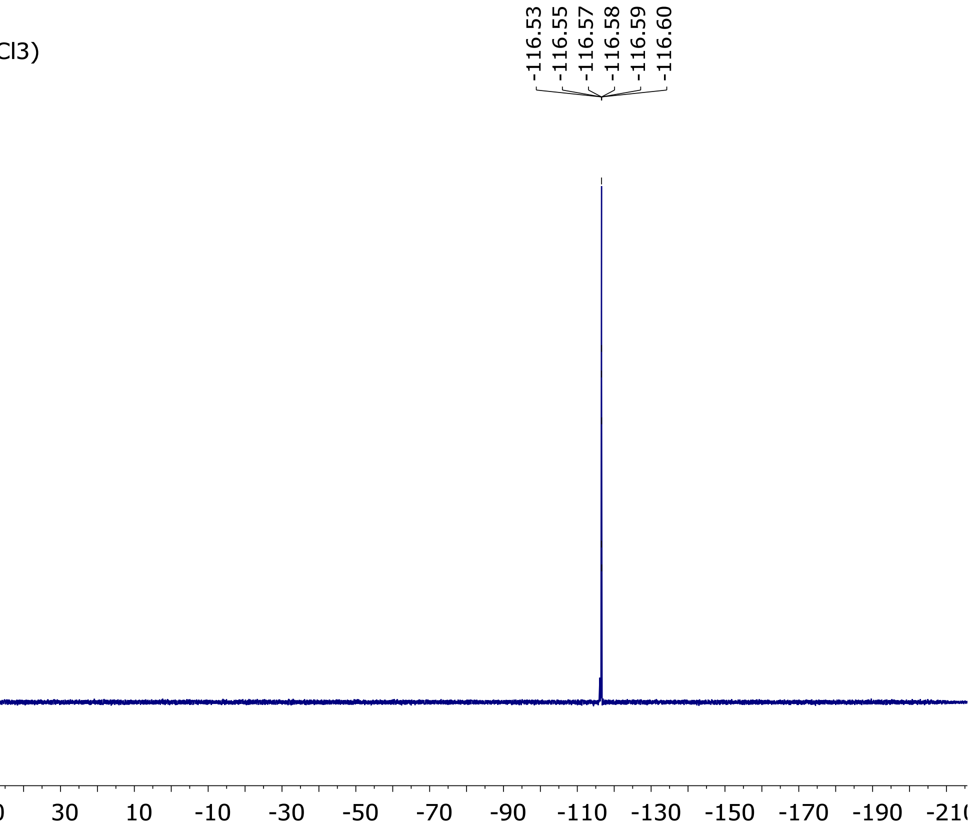

Supplement: Supplementary file 1 — Supplementary [file ANIE-59-2115-s001.pdf]
